# Structure-Based Design of Potent and Selective MerTK Inhibitors by Modulating the Conformation of αC Helix

**DOI:** 10.1021/acs.jmedchem.4c03092

**Published:** 2025-05-20

**Authors:** Yi-Hui Peng, Mu-Chun Li, Wan-Ching Yen, Teng-Kuang Yeh, Ching-Cheng Hsueh, Fu-Ming Kuo, You-Liang Lai, Ling Chang, Lung-Chun Lee, Pei-Yi Chen, Kuei-Jung Yen, Teng-Yuan Chang, Hsu-Yi Sun, Chun-Yu Chang, Su-Huei Hsieh, Chen-Ming Yang, Hsing-Pang Hsieh, Su-Ying Wu

**Affiliations:** † Institute of Biotechnology and Pharmaceutical Research, 50115 National Health Research Institutes, 35, Keyan Road, Miaoli County, Zhunan Town 350, Taiwan, R.O.C.; ‡ Department of Chemistry, National Tsing Hua University, Hsinchu City 300044, Taiwan, R.O.C

## Abstract

Tumor-associated macrophages play an important role in cancer progression and immunosuppression, making their receptors promising therapeutic targets. MerTK, a TAM receptor, regulates macrophage efferocytosis and polarization, and its inhibition holds potential for tumor growth suppression and immune modulation. However, Tyro3, another TAM receptor, is involved in neurogenesis, highlighting the need to selectively target MerTK while avoiding Tyro3 inhibition to prevent neurotoxicity. In this study, we present a novel strategy for designing MerTK-selective inhibitors by modulating the conformational dynamics of its αC helix. By integrating structural biology, medicinal chemistry, protein stabilization assays, and molecular docking studies, we identified compound **11**, which demonstrates potent inhibition and selectivity for MerTK. Pharmacokinetic evaluations and *in vivo* studies further reveal compound **11** as a promising candidate for further development. Our findings not only advance the understanding of the MerTK-specific mechanism but also propose a strategy for designing selective kinase inhibitors targeting the αC helix conformation.

## Graphical abstract

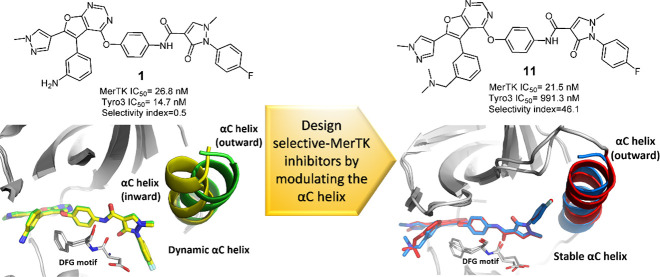

## Introduction

Tumor-associated macrophages, pivotal cellular components of the tumor microenvironment, act as critical mediators of cancer progression and immune evasion.

Tyro3, Axl, and MerTK, collectively known as TAM receptors, are potential therapeutic targets on macrophages and are key regulators of immune homeostasis, the innate immune response, and macrophage efferocytosis. TAM receptor signaling has been shown to influence macrophage polarization, a process in which macrophages differentiate into distinct phenotypes and functional states in response to the signals they receive from their environment. This process leads to the differentiation of macrophages into two primary functional states: the M1 macrophages, which are pro-inflammatory and play a critical role in immune defense, and the M2 macrophages, which are anti-inflammatory and contribute to tissue repair, suppression of T-cell infiltration, and resolution of inflammation.^1,2^

MerTK has been shown to reduce M1 polarization while promoting the differentiation of macrophages into the M2 phenotype.^1^ This shift in macrophage polarization favors an immunosuppressive environment that can support tumor progression. Additionally, MerTK plays a prominent role in regulating efferocytosis, the process by which macrophages engulf and clear apoptotic cells. Efferocytosis is crucial for maintaining tissue homeostasis and preventing excessive inflammation.^3^ MerTK is activated upon binding to its ligands, Protein S or growth arrest-specific 6 (GAS6), which recognize phosphatidylserine (PtdSer) exposed on the surface of apoptotic cell debris. Macrophages with activated MerTK initiate the engulfment of apoptotic cells.^4^ MerTK expression is higher in M2-like macrophages than in unstimulated or M1-like macrophages. This elevated MerTK expression is evident in various cancer types, including acute leukemia (ALL or AML),^5,6^ melanoma,^7,8^ lung cancer,^9^ and prostate cancer.^10^ Inhibiting MerTK has been shown to induce tumor cell death and activate the innate immune response, suggesting its potential as a therapeutic target.^11^

Unlike MerTK, Tyro3′s role in macrophage polarization is less understood, as it is predominantly expressed in the nervous system.^1,12^ Tyro3 is essential for prolonging the lifespan and survival of neuropeptide Y (Npy) neurons in the mouse anorexia (anx) mutation model.^13^ It is involved in regulating hemostasis and thrombosis.^14^ In Tyro3-deficient mouse models, cortical and hippocampal synapses fail to complete end-stage differentiation, remaining electrophysiologically and ultrastructurally immature.^15^ Tyro’3 expression in neurons and its role in regulating neurogenesis and differentiation emphasize the need for careful consideration of its involvement in cancer therapies targeting TAM receptors. Therefore, the development of inhibitors targeting MerTK while avoiding inhibition of Tyro3 holds promise to suppress tumor growth and alleviate immunosuppression within the tumor microenvironment, without inducing neurotoxicity.

To date, only a limited number of MerTK inhibitors have been designed and developed as immunomodulatory agents. MRX-2843 (UNC2371)^16–18^ is a MerTK inhibitor and immune modulator currently undergoing Phase 1 clinical trials for cancer therapy. Preclinical studies have demonstrated its promising efficacy in targeting MerTK-mediated tumor growth and immune suppression. MRX-2843 is being evaluated in clinical trials for its safety, tolerability, and therapeutic potential, both as a monotherapy and in combination with other therapeutic agents, in the treatment of solid tumors, hematologic malignancies, and nonsmall cell lung cancer (NSCLC). Preliminary data suggest that MRX-2843 may help overcome resistance mechanisms, enhance immune responses, and improve clinical outcomes in patients. ONO-7475,^19–21^ targeting Axl and MerTK, is currently undergoing phase 1 clinical trials for its potential use in cancer therapy. Preclinical and early clinical studies suggest its potential both as a monotherapy and in combination with other agents for treating solid tumors and acute myeloid leukemia (AML). Despite these promising findings, most MerTK inhibitors remain in the early stages of clinical trials or preclinical development, and further investigation is required to fully assess their therapeutic potential. Therefore, the continued design of novel inhibitors that selectively target MerTK while minimizing off-target inhibition of Tyro3 remains important for advancing the development of targeted immunotherapies.

Protein kinases are important targets for many diseases. Members of the same kinase family often share similar biological functions and protein structures. To reduce unexpected side effects and off-target interactions, the selectivity issue of a kinase inhibitor is therefore a major concern. Previous studies have suggested that designing selective kinase inhibitors can be achieved through allosteric modulation by displacing the αC helix.^22^ The αC helix plays a crucial role in the structure of protein kinases, with its position and dynamic behavior being essential for kinase activity. By designing inhibitors that specifically bind to the αC helix, these inhibitors can induce displacement of the αC helix, thereby altering the kinase conformation and modulating its activity.^23–25^ For example, the binding of two 8-anilino-1-naphthalenesulfonate molecules to the binding pocket of CDK2 induces a conformational change, specifically the displacement of the αC helix. This conformational change results in the inhibition of kinase activity and enhances the selectivity of the inhibitor within the CDK kinase family.^24^ Additionally, lapatinib, a clinically used anticancer drug targeting EGFR and HER2, has been shown to reposition the αC helix from an active to an inactive conformation. This displacement impedes the formation of the active kinase complex, thereby disrupting downstream signaling.^26^ Moreover, the difference in the position and flexibility of αC helix provides the structural rationale for the selectivity of protein kinase D1 inhibitor over NF-κB inducing kinase.^27^ Furthermore, the analogues of dasatinib, which incorporate an additional phenoxy group, induce the outward displacement of the αC-helix. This modification significantly enhances the selectivity of dasatinib, an inhibitor targeting multiple kinases.^28^

In this study, the pan-TAM inhibitor **1** (Table 1) served as a starting compound for further development. Our strategy involved modulating the αC helix structure of MerTK upon inhibitor binding to facilitate lead optimization. This approach led to the identification of a selective MerTK inhibitor, **11**, which demonstrates enhanced selectivity over Tyro3. Structural analysis of MerTK in complex with **11** revealed **11** adopts a unique upward binding pose, forming extensive interactions with the αC helix. In contrast, **1** adopts a downward binding pose and lacks significant interactions with the αC helix. Comprehensive structure–activity relationship (SAR) analyses, structural biology investigations, protein stabilization assays and molecular docking studies were performed to elucidate the inhibitory mechanism of this series of compounds against MerTK. Moreover, pharmacokinetic evaluations and *in vivo* studies reveals **11** functions as an effective immunomodulatory agent, with its antitumor efficacy attributed to the remodeling of the tumor immune microenvironment. These findings provide insights for improving selectivity by stabilizing the highly mobile αC helix with a unique kinase conformation and hold promise for advancing cancer research by targeting TAM family.

**Table 1:**
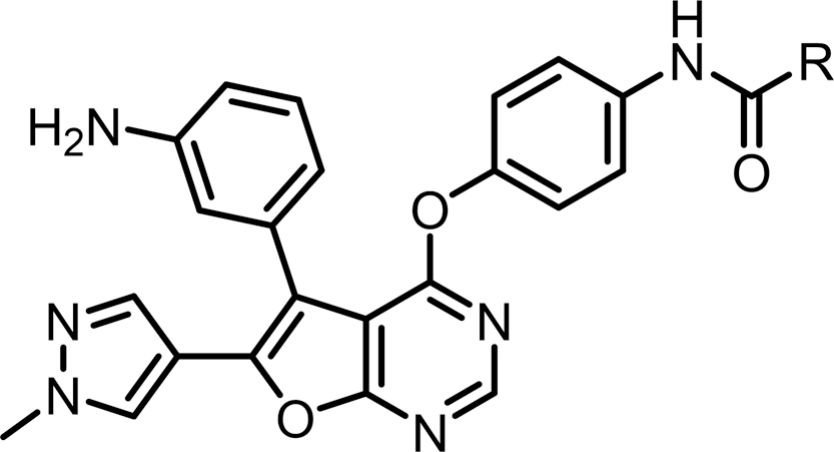

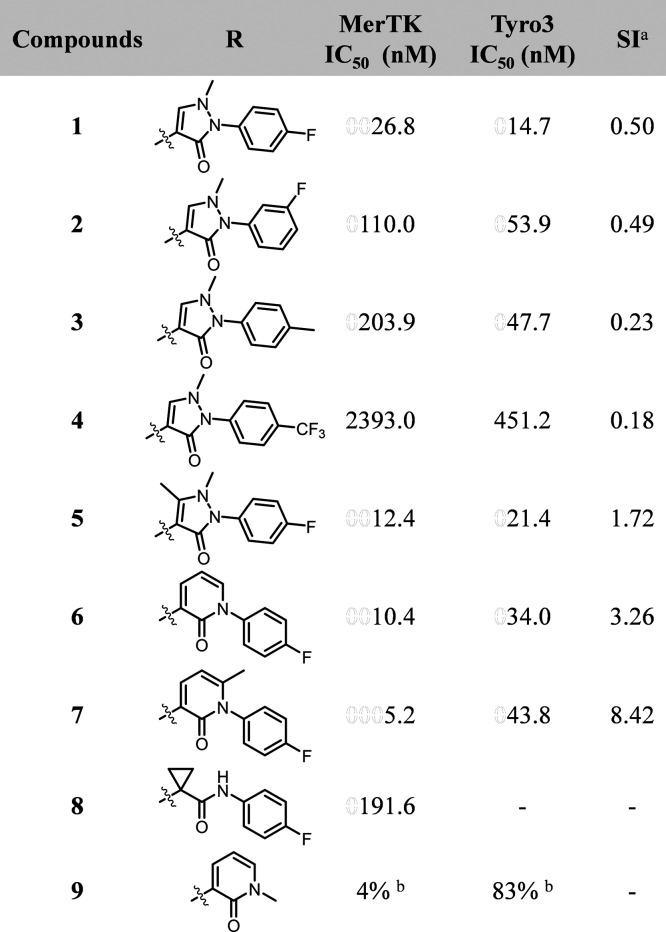
Inhibition of MerTK and Tyro3 with Furo­[2,3-*d*]­pyrimidine-Based Derivatives^a^

a ^a^SI: Selectivity index, representing the selectivity of MerTK over Tyro3. SI is calculated as the ratio of IC_50_ for Tyro3 to IC_50_ for MerTK. All data are reported as the means of at least two independent experiments, with most values having error margins within 15%. ^b^ Inhibition assays were conducted using 1 μM compound.

## Results and Discussion

### Chemistry

The synthesis of inhibitors **1**–**21** is presented in Schemes 1 and 2, with **6**, **10**, **15**, and **18** previously described in our earlier study.^29^ Starting with aniline **22**, treatment with various carboxylic acids (**27a**–**27e**), either commercially available or presynthesized, yielded carboxamides **23a**–**23e** in moderate yields. There carboxamides **23a**–**23e** were subsequently subjected to Suzuki coupling with (3-aminophenyl)­boronic acid to produce **1**–**5**. Similarly, **11**–**14** and **19** were synthesized from carboxamides **23a**–**23d** via Suzuki coupling with the boronic ester featuring an *N*,*N*-(dimethylamino)­methyl functional group at either the *meta*- or *para*-position. **16** was derived from **1** through a reaction with acetyl chloride under basic conditions. For the preparation of **17** and **21**, the intermediate **23a** was initially coupled with (3-formylphenyl)­boronic acid and (4-formylphenyl)­boronic acid, respectively, followed by reductive amination of **24a** and **24b** utilizing 1-methylpiperazine and sodium triacetoxyborohydride.

**Scheme 1:**
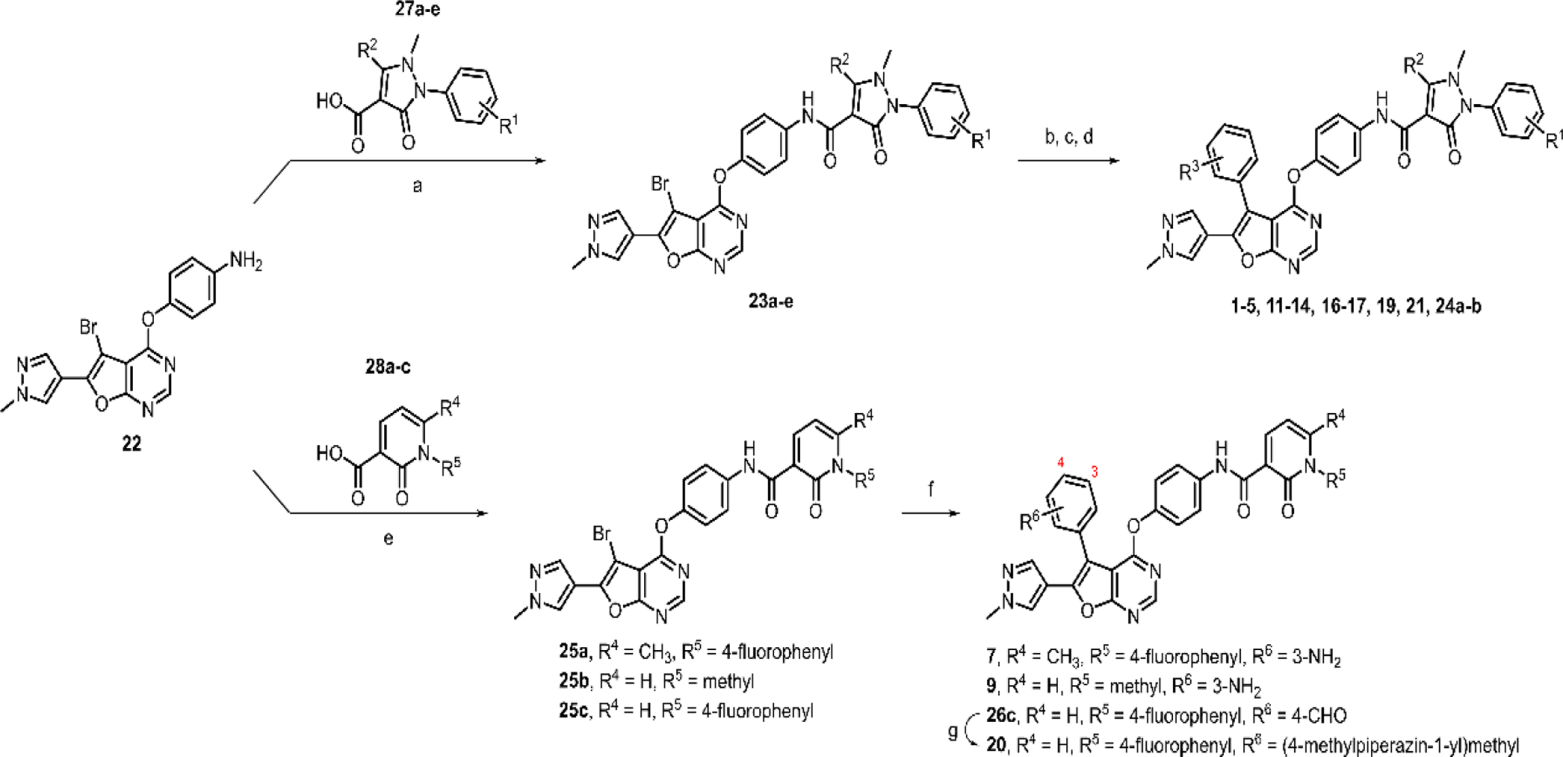
Synthetic Route for Inhibitors **1**–**7** and **9**–**21** ^a^

a Reagents and conditions: (a) carboxylic acids **27a**–**27e**, DIPEA, TBTU, DMF, rt, 12–16 h, 13–92%; (b) appropriate boronic acids or boronic esters, Pd­(dppf)­Cl_2_, Na_2_CO_3_, appropriate solvents, 80–110 °C, 3–16 h, 15–94%; (c) from **1**, acetyl chloride, Et_3_N, CH_2_Cl_2_, rt, 16 h, 25%; (d) from **24a–24b**, 1-methylpiperazine, NaBH­(OAc)_3_, CH_2_Cl_2_, rt, 16 h, 68–75%; (e) carboxylic acids **28a**–**28b**, DIPEA, TBTU, DMF, rt, 10–16 h, 66–97%; (f) appropriate boronic acids, Pd­(dppf)­Cl_2_, Na_2_CO_3_, appropriate solvents, 80–110 °C, 2–16 h, 44–63%; (g) from **26c**, 1-methylpiperazine, NaBH­(OAc)_3_, CH_2_Cl_2_, rt, 16 h, 48%

**Scheme 2:**
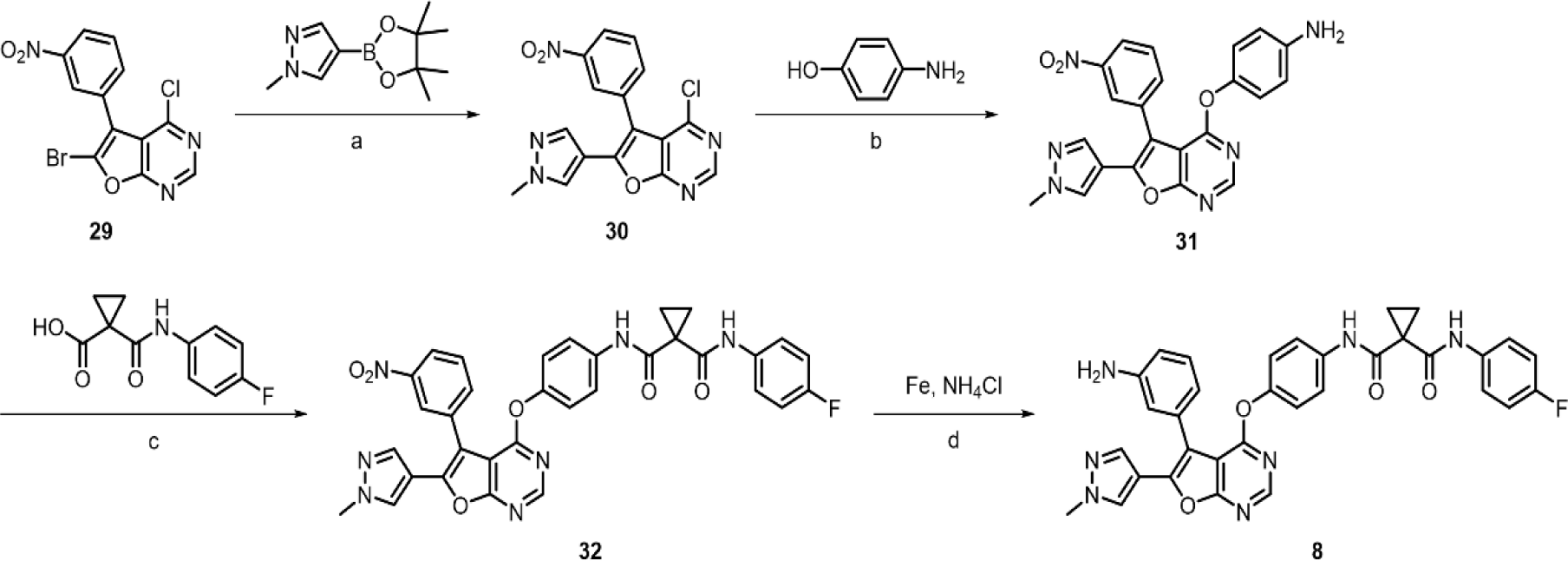
Synthetic Route for Inhibitor **8** ^a^

a Reagents and conditions: (a) 1-methyl-4-(4,4,5,5-tetramethyl-1,3,2-dioxaborolan-2-yl)-1*H*-pyrazole, Pd­(dppf)­Cl_2_, Na_2_CO_3_, 1,4-dioxane, 80 °C, 24 h, 72%; (b) 4-aminophenol, NaH, DMF, rt, 16 h, 99%; (c) 1-[(4-fluorophenyl)­carbamoyl]­cyclopropane-1-carboxylic acid, EDCI, CH_2_Cl_2_, rt, 4.5 h, 73%; (d) iron powder, sat. NH_4_Cl­(aq), ethanol, CH_2_Cl_2_, H_2_O, 80 °C, 10 h, 37%

In a similar manner, aniline **22** was coupled with presynthesized carboxylic acids **28a**–**28c** to afford carboxamides **25a**–**25c**. For **7** and **9**, Suzuki coupling of carboxamides **25a** and **25b** with (3-aminophenyl)­boronic acid proceeded in good yields. Meanwhile, carboxamide **25c** was converted into carboxamide **26c** via Suzuki coupling with (4-formylphenyl)­boronic acid, which was subsequently subjected to reductive amination with 1-methylpiperazine and sodium triacetoxyborohydride, yielding **20** in a moderate yield.

The synthesis of **8** began with the Suzuki coupling of **29** utilizing the boronic ester containing an *N*-methyl pyrazole group in 1,4-dioxane. Subsequently, the 4-chloride of **30** was reacted with 4-aminophenol to produce aniline **31**. The aniline **31** was then coupled with 1-[(4-fluorophenyl)­carbamoyl]­cyclopropane-1-carboxylic acid, yielding **32** in 73% yield. Finally, the nitro group of **32** was transformed into an amino group via an iron-catalyzed reduction, affording the desired **8**.

### Structural Biology Studies of Furo­[2,3-*d*]­pyrimidine-Based Derivatives

**1** is a furo­[2,3-*d*]­pyrimidine-based pan-TAM inhibitor (Table 1). To elucidate the binding mechanisms of compound **1** and guide the design of selective MerTK inhibitors derived from a pan-TAM scaffold by modulating the interactions between the inhibitors and αC helix, the structural biology study was conducted to determine the crystal structure of MerTK in complex with **1** (Table 2 and Figure 1; PDB 9KRY). In MerTK/**1** complex structure, the crystal asymmetric unit contains two protein molecules, monomer A (chain A) and monomer B (chain B). **1** occupies the ATP binding site of each monomer and the binding of **1** induces the DFG out conformation of MerTK. The furo­[2,3-*d*]­pyrimidine core structure (Ring 4 and Ring 5) of **1** forms a hydrogen bond with M674 in the hinge region. The fluorobenzene (Ring 1) of **1** is positioned in the allosteric region (Figure 1A), where it engages in extensive interactions with surroundings. These interactions include hydrophobic interactions with D741 on the DFG motif and F719 in the allosteric region. Additionally, there is a C–F···C short contact between the fluorine atom of the benzene ring and the side chain carbon of L714 in the allosteric region (Figure 1A). The contact angle (θ) for the C–F···C short contact is 133.4° with a contact distance (d) of 3.3 Å in monomer A while the contact angle (θ) is 152.3° with a contact distance (d) of 3.1 Å in monomer B. These measurements are consistent with the polar scatter plot range and align with the most frequently observed values in the histogram distribution for fluorine-involved short contacts, thereby confirming the presence of the C–F···C contact.^30^ The 1-methylpyrazol-3-one (Ring-2) is situated between the DFG motif and the αC helix, where it forms a hydrogen bond with D741. Moreover, the carbonyl group at the amide linker forms a hydrogen bond with K619, thereby disrupting the conserved salt bridge between K619 in β strand and E637 in αC helix. The phenyl (Ring-3) aligns closely with the DFG motif and forms strong hydrophobic interactions with L671, K619, V601 A740 and F742. The aniline (Ring 6) moiety, located near the solvent-accessible region, forms a hydrogen bond with D678 in the hinge through its amine group and makes a π-stacking interaction with F742 as well as hydrophobic interactions with V601 and L593 in the P-loop. The methylpyrazole moiety (Ring-7) extends to the solvent-accessible region and does not form any interactions with MerTK.

**Table 2:** Statistic of X-ray Diffraction and Structure Refinement for MerTK Complex Structures

PDB entry	9KRY	9KS9	9KRZ
Compound	1	6	11
**Resolution**(Å)	30–2.25	45.67–2.80	30–2.62
**Space group**	P2_1_	P2_1_	P2_1_
**Unit cell (α=γ=90°)**			
**a**(Å)	50.994	51.555	50.577
**b**(Å)	91.279	91.351	91.427
**c**(Å)	69.227	69.837	68.937
**β**(°)	100.770	101.000	100.384
**R**_**merge**_^**a**^(%) or R_**meas**_^**b**^(%)	6.8/47.2^a^	12.5/25.3^b^	7.2/44.2^a^
**I/σ (all/outer)**	16.84/2.28	14.39/6.77	14.7/2.26
**Unique reflection**	29677	14628	18871
**Completeness (%)(all/outer)**	98.5/91.8	92.7/94.7	98.8/92.4
*R*_**work**_ **/** *R*_**free**_	23.96/28.44	20.63/26.03	22.13/26.72
**rmsd Bond lengths** (Å)	0.003	0.0022	0.002
**rmsd Bond angles** (°)	0.824	0.620	0.635
**Ramachandran**			
**Favored** (%)	100	100	100
**Outlier** (%)	0	0	0

**Figure 1:**
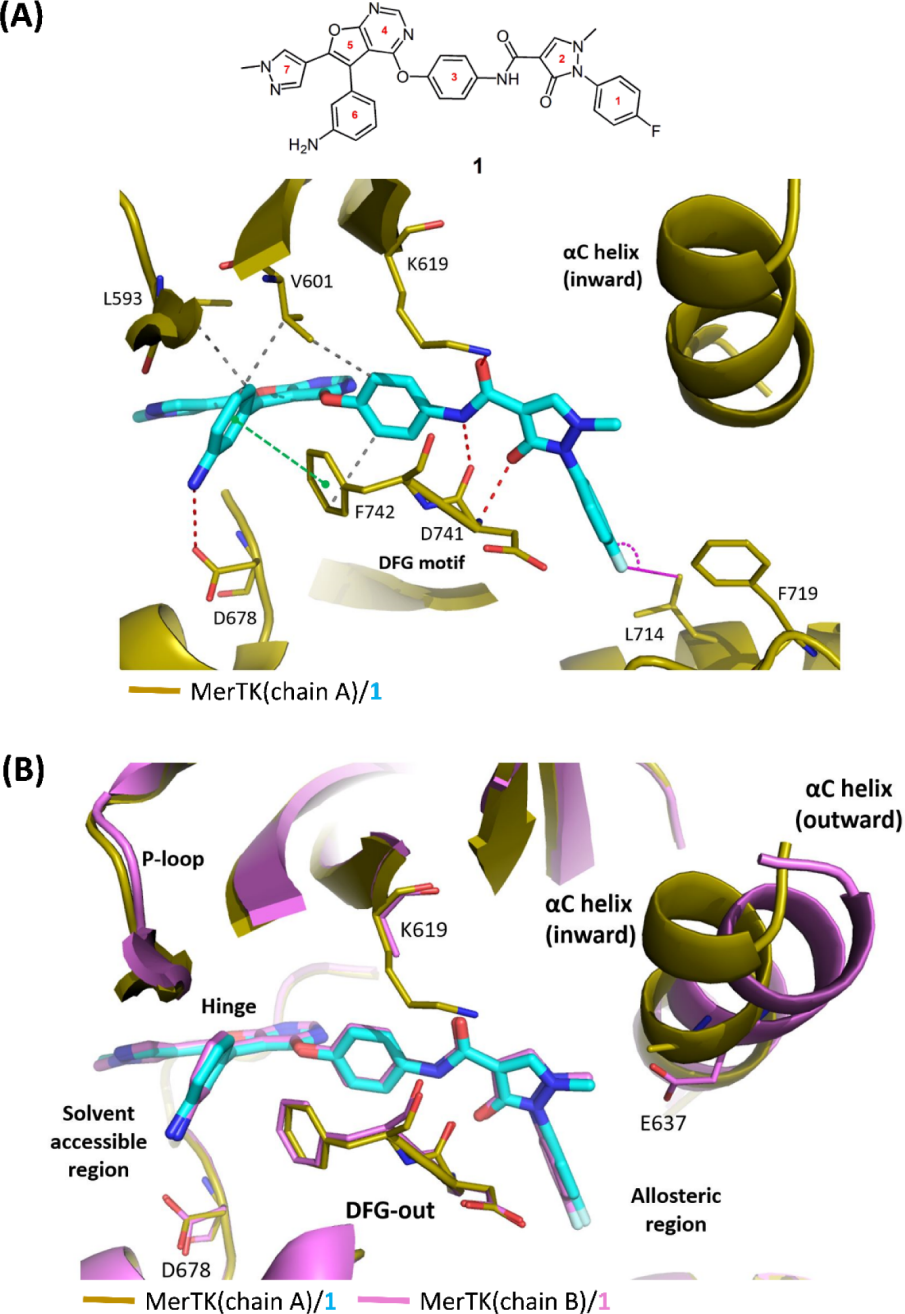
(A) Structure of **1** with labeled ring numbers. The crystal structure of MerTK bound to **1** (PDB 9KRY) highlights residues surrounding the inhibitor in chain A. π-stacking interactions are indicated in green, hydrophobic interactions in gray, and hydrogen bonds in red. C–F···C short contacts between the para-fluorine of the 1-fluorobenzene ring of **1** and L714 in the allosteric region of MerTK are depicted as pink lines. (B) Superimposition of the structures of MerTK­(chain A)/ **1** with MerTK­(chain B)/**1**. Chain A (gold) exhibits an αC-inward conformation while chain B (pink) exhibits an αC-outward conformation. In the chain A of MerTK/**1** complex structure, The electron density maps for the side chain of E637 residue in chain A and K619 in chain B were unclear and therefore excluded from the structure.

It is noteworthy, as revealed in the MerTK/**1** structure, **1** does not interact with the αC helix despite Ring-2 of **1** being in close proximity to the αC helix. Furthermore, the αC helix of MerTK exhibits dynamic behavior, adopting different conformations in each monomer (Figure 1B). In monomer A, the αC helix is dynamic, displaying a poorly defined density map and adopting an inward conformation. In contrast, in monomer B, the αC helix shows a clearer density map and adopts an outward conformation. These observations highlight the structural flexibility of the αC helix.

### Structure-Guided Lead Optimization of Furo­[2,3-*d*]­pyrimidine-Based Derivatives

Lead optimization was initially focused on structural modifications of Ring-1 and Ring-2 of **1** (Table 1). These moieties, located near the αC helix, were modified to enhance their interactions with the αC helix, aiming to improve both potency and selectivity of the compound.

Shifting the fluorine atom from the para (**1**) to the meta position resulted in a 4-fold reduction in potency for **2** against MerTK (Table 1). This decreased activity is likely attributed to the meta-positioned fluorine being farther from L714, thereby weakening the C–F···C interaction. This modification highlights the strong directionality of fluorine-mediated interactions.

Furthermore, replacing the fluorine atom with a methyl group in **3** led to an 8-fold reduction in activity, highlighting the significant role of the C–F···C interaction in the compound’s inhibitory activity. Additionally, substituting the para-fluorine in **1** with a larger trifluoromethyl group in **4** led to a dramatic reduction in potency. The increased steric bulk of the trifluoromethyl group likely introduces steric hindrance with surrounding residues and may impede entry into the allosteric back pocket due to obstruction by the entrance channel.

Subsequent structural modifications were focused on Ring-2. The introduction of bulkier substituents, such as 2,3-dimethylpyrazolone **5** and pyridone **6**, resulted in a 2-fold increase in potency against MerTK compared to **1**. The more substantial substituent, 6-methyl-2-pyridone **7**, demonstrated a 5-fold improvement in MerTK potency. Conversely, substitution with a smaller moiety, such as cyclopropanecarboxamide **8**, led to a 7-fold decrease in MerTK potency relative to **1**. These structure–activity relationship (SAR) results indicate that increasing the size of the Ring-2 substituent correlates with enhanced MerTK potency.

Finally, removing the 1-fluorobenzene group (Ring 1) resulted in a dramatic loss of MerTK activity in **9**, highlighting the critical role of the 1-fluorobenzene moiety in maintaining the compound’s potency.

To investigate the impact of a bulkier substituent in Ring-2 on MerTK potency, we solved the structure of the MerTK complex with **6** (Table 2). The MerTK/**6** structure reveals that the bulkier pyridine moiety (Ring-2) of **6** occupies the same binding site as 1-methylpyrazol-3-one in **1**. However, the bulkier pyridine moiety forms additional close contact interactions with E637 and V669 (Figure 2), which contribute the increased potency of **6** against MerTK.

**Figure 2:**
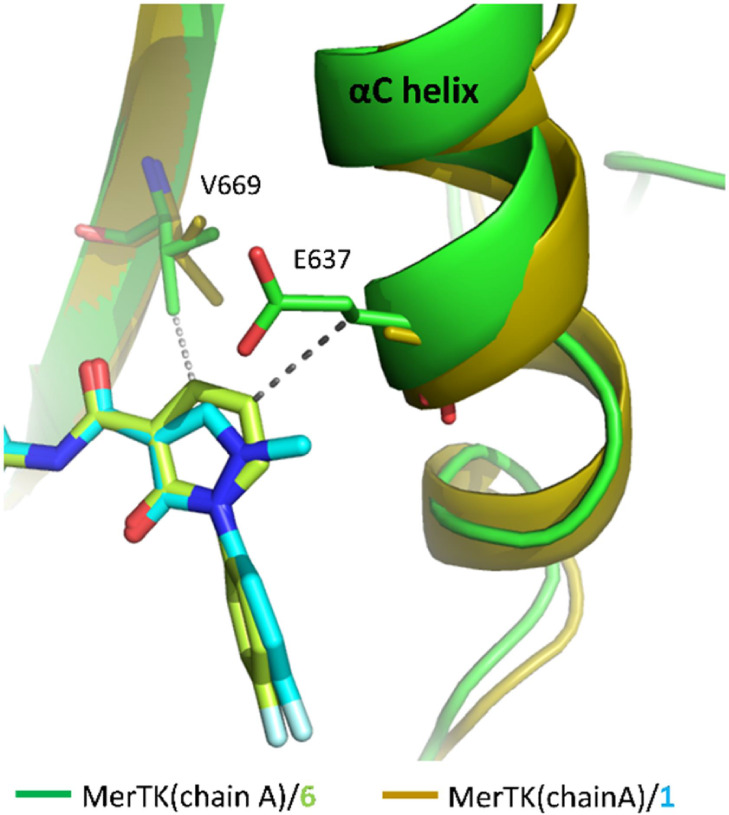
Crystal structures of MerTK bound to compound **6** (PDB: 9KS9) and compound **1** (PDB: 9KRY) were superimposed to analyze structural differences and interactions. Close contacts formed by the MerTK/**6** complex were represented using gray dashed lines. In the crystal structure of MerTK/**1**, the electron density map for the side chain of E637 residue was not well-defined and therefore excluded from the structure.

Although **6** showed increased potency toward MerTK, its selectivity for Tyro 3 remains suboptimal. The selectivity index of **6**, defined as the ratio of the IC_50_ of Tyro 3 to the IC_50_ of MerTK, is relatively low (SI= 3.26). Structural analysis of the MerTK/**6** complex reveals that **6** interacts weakly with the αC helix. Moreover, the αC helix exhibits dynamic behavior, similar to that in MerTK/**1**, adopting two distinct conformations within each monomer. To enhance selectivity and strengthen the interactions between the inhibitors and the αC helix of MerTK, modifications to the tail region were pursued (Table 3). Given that Ring-4 and Ring-5 form the core scaffold structure and Ring-3 interacts well with the DFG motif and surrounding residues, these moieties were retained.

**Table 3:**
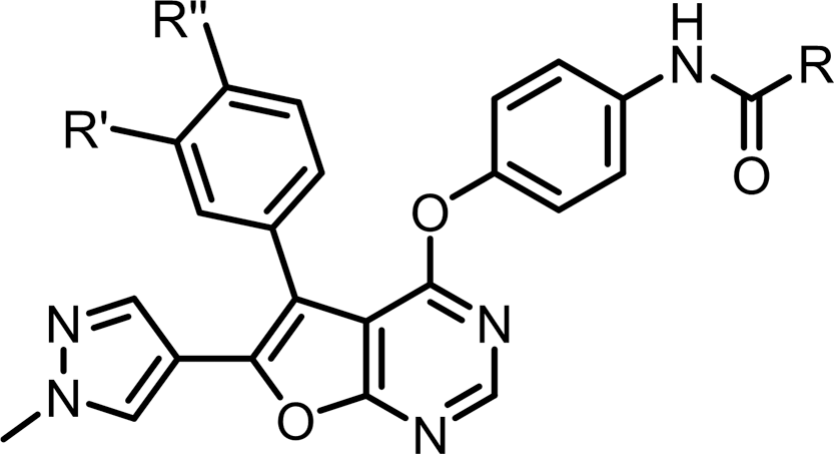

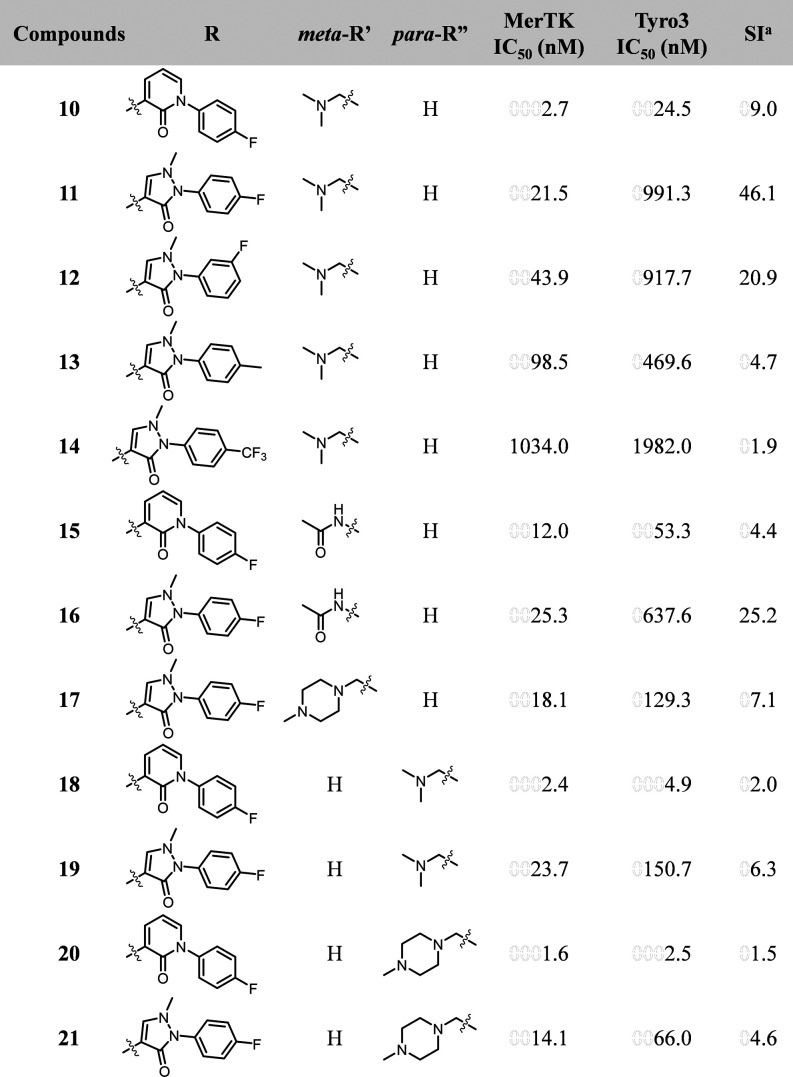
SAR Exploration of Meta and Para Site of Tail Part^a^

a ^a^SI: Selectivity index, representing the selectivity of MerTK over Tyro3. SI is calculated as the ratio of IC_50_ for Tyro3 to IC_50_ for MerTK. All data are reported as the means of at least two independent experiments, with most values having error margins within 15%.

### Structural Modification on the Tail Region of Furo­[2,3-*d*]­pyrimidine-Based Derivatives Improve the Selectivity

A longer *N,N*-dimethylethanamine moiety was introduced at the *meta*-position (R’) of Ring-6 to extend the molecular length and enhance binding to the αC helix, resulting in the derivative **10**. **10** exhibits a 4-fold increase in MerTK inhibition and improved selectivity, with the SI rising to 9.0 as compared to **6**.

Given the increased activity and enhanced selectivity observed with the incorporation of the *N,N*-dimethylethanamine moiety in **10**, further investigations are being conducted to evaluate the effects of integrating this tail moiety into various head groups at the R site (Table 3). When substituted with an *N,N*-dimethylethanamine moiety at the *meta*-R’ position, compounds **11**, **12**, **13** and **14** exhibit more potent MerTK inhibition compared to their counterparts with an amine substitution at the same position (compound **1**, **2**, **3** and **4)**. However, surprisingly, all these derivatives show a dramatic decrease in Tyro3 potency (Table 3). Notably, **11** shows particularly weak Tyro3 activity, with an IC_50_ of 991.3 nM, resulting in a significantly improved selectivity index (SI) of 46.1. Similarly, **12** demonstrates reduced Tyro3 activity, with an IC_50_ of 917.7 nM, and a high SI of 20.9. In comparison, compounds **13** and **14**, while showing reduced activity against Tyro3, exhibit a lower SI.

We further explore the structure–activity relationship (SAR) of different tail moiety, acetamide (Table 3). When the acetamide is substituted at the *meta*-R’ position of **10** (and **11**), the resulting compounds **15** (and **16**) maintain MerTK potency. However, their selectivity indices (SI) are lower than those of the *N,N*-dimethylethanamine counterparts, **10** and **11**. Further modification involving the substitution of the *N,N*-dimethylethanamine moiety in **11** with a ring-shaped 1,4-dimethylpiperazine yielded **17** (Table 3). **17** shows increased activity against Tyro3, leading to a lower SI of 7.1 compared to **11** (SI = 46.1).

Lastly, we investigated the impact of tail positioning on the activity and selectivity for MerTK. Shifting the *N,N*-dimethylethanamine moiety from the *meta*-position as seen in **10** and **11** to the *para*-position yields compounds **18** and **19**, respectively. Both compounds exhibit increased Tyro3 activity, leading to decreased selectivity for MerTK, with SIs of 2.0 and 6.3, respectively. Similarly, the substitution of the 1,4-dimethylpiperazine ring at the *para*-position (**20** and **21**) does not enhance MerTK selectivity over Tyro3 (Table 3).

In conclusion, the incorporation of long *N*,*N*-dimethylethanamine moiety at the *meta*-R’ position in Ring-6 results in a significant decrease in Tyro3 activity, thereby enhancing selectivity for MerTK. Additionally, the positioning of this moiety at the substitution site on Ring-6 also plays an important role in modulating selectivity. These findings highlight the importance of the chemical structure and spatial configuration of the compound in optimizing both potency and selectivity.

### Structure Biology Studies of MerTK in Complex with 11

Since **11** demonstrates highest selectivity with SI of 46.1 among these synthesized inhibitors, the cocrystal structure of MerTK in complex with **11** was resolved to investigate its binding mechanism and molecular basis of the selectivity.

The structure of MerTK/**11** was determined to a resolution of 2.62Å (Table 2; PDB 9KRZ) and the density map clearly shows **11** adopts a unique binding mode with Mertk. The headgroup of **11** orients upward to target the αC helix, while the *N*,*N*-dimethylethanamine moiety forms a salt bridge with D678 in the hinge region (Figure 3A). This upward binding of **11** contrasts with the previously resolved structures—**1** and **6**—which exhibit a downward orientation that extends into the allosteric pocket. Furthermore, the upward positioning of **11** allows the 1-fluorobenzene moiety to form hydrophobic interactions with F634 and A638 on the αC helix and V669 on the β-sheet. Additionally, the *para*-fluorine of 1-fluorobenzene forms a C–F···O interaction with F634. The acetamide linker forms a hydrogen bond with the backbone of D741, locking MerTK in a DFG-out conformation. Moreover, upon the binding to **11**, αC helix of MerTK becomes more stable and adopts an outward conformation in both monomers (Figure 3B) whereas αC helix is more dynamic and adopts two different conformations upon the binding to **1** or **6**. The electron density is also clear and more continuous for αC helix, adjacent β3-αC loop and P-loop, indicating **11** stabilizes αC helix and its surrounding areas.

**Figure 3:**
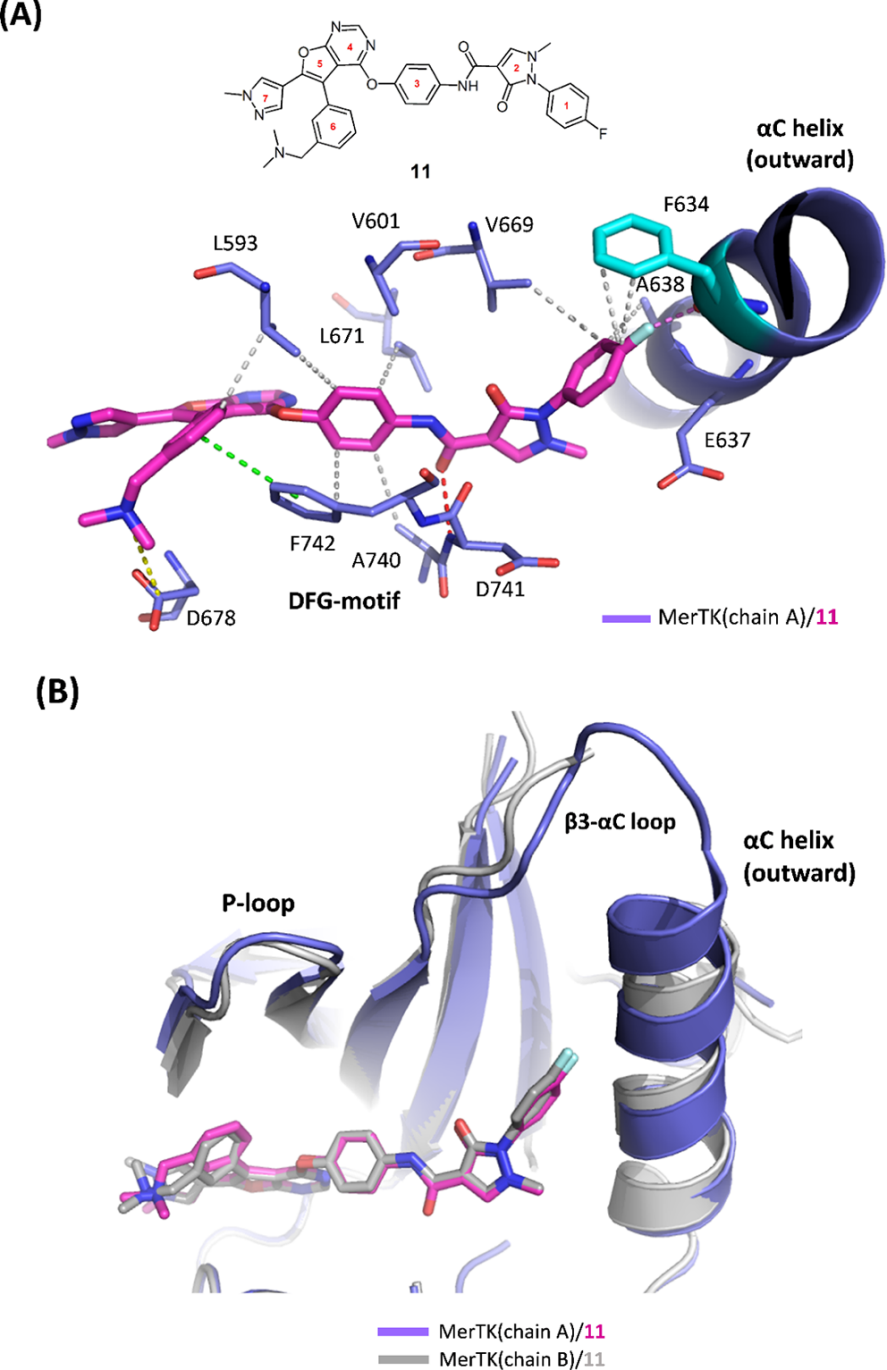
(A) Structure of compound **11** with labeled ring numbers. The crystal structure of the MerTK/**11** complex reveals an upward binding orientation of **11** approaching the αC helix (PDB: 9KRZ). Key interactions are highlighted: hydrogen bonds (red dashed lines), salt bridge (yellow dashed line), C–F···O short contact (magenta dashed lines), hydrophobic interactions (gray dashed lines), and π-stacking interactions (green dashed lines). (B) Superimposition of chain A (purple/magenta) and chain B (gray/gray) of the MerTK/**11** structure.

In summary, **11** exhibits a distinct upward binding mode with MerTK, which stabilizes the αC through extensive interactions with both the αC helix and adjacent regions.

### Comparison of the Structures of MerTK/11 and MerTK/1

Superimposition of the structures of MerTK/**11** and MerTK/**1** (chain A) reveal that these two compounds adopt distinct binding modes, despite their high similarity in chemical structure. The differences include the shift in the DFG motif, the flip of the acetamide linker and 1-methylpyrazol-3-one and the change in the orientation of the 1-fluorobenzene moiety (Figure 4A,D,E).

**Figure 4:**
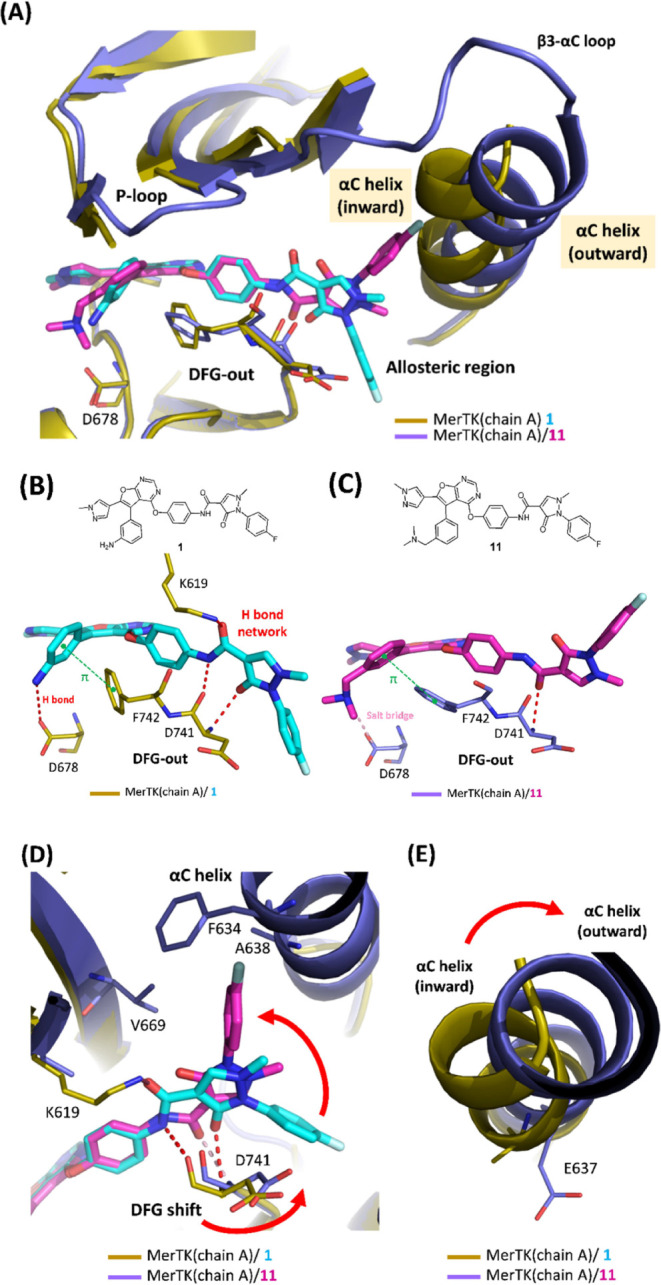
(A) Superimposition of the complex crystal structures of MerTK/**11** (PDB: 9KRZ) and MerTK/**1** (PDB: 9KRY). (B) Hydrogen bond network interactions observed in the MerTK/**1**complex. (C) Loss of hydrogen bond network interactions in the MerTK/**11** complex. Hydrogen bonds are represented as red dashed lines, edge-π interactions as green dashed lines, and salt bridges as yellow dashed lines. (D) DFG motif shift and change in the orientation of 1-fluorobenzene moiety upon binding of **11** to MerTK compared with **1**. Hydrogen bonds between **1** and MerTK are represented by red dashed lines, while the hydrogen bond between **11** and MerTK is represented by pink dashed lines. (E) Outward shift of the αC helix observed when **11** binds to MerTK compared with **1**. The electron density map for the side chains of residues K619 and E637 was not well-defined. Consequently, the side chains for these residues were excluded from the structure.

As revealed in the MerTK/**1** structure, the Ring-6 with methanamine substitution forms the hydrophobic interactions with F742 on the DFG motif and a hydrogen bond with D678 (Figure 4B). The acetamide linker and 1-methylpyrazol-3-one moiety of **1** form the extensive hydrogen bond network with the DFG motif and K619 in β3 sheet (Figure 4B). However, in the MerTK/**11** structure, the introduction of the longer *N*,*N*-dimethylethanamine substitution on Ring-6 results in the shift of adjacent DFG motif to accommodate this longer tail (Figure 4C,D). Additionally, the flip of the acetamide linker and 1-methylpyrazol-3-one have been observed. These alterations result in the loss of the hydrogen bond network seen in MerTK/**1**. Instead, the carbonyl oxygen of the acetamide linker in **11** is oriented downward and forms a hydrogen bond with D741 (Figure 4C). The flip of the acetamide linker and 1-methylpyrazol-3-one consequently causes the headgroup to orient upward, facilitating interactions with the αC helix and β sheets of MerTK (Figure 4D). Moreover, the αC helix adopts an outward conformation in both monomers upon binding to **11** whereas it exhibits an inward conformation in monomer A when bound to **1** (Figure 4E).

### Investigation of Protein Stability upon Binding of Furo­[2,3-*d*]­pyrimidine-Based Derivatives

To further investigate protein stability of MerTK upon binding of **11** and **1**, a thermal shift assay (TSA) was conducted (Figure 5). The thermal shift assay (TSA) is a technique used to assess the stability of protein by measuring the protein’s melting temperature (Tm).^31,32^ When a protein is heated, it gradually unfolds, and the Tm indicates the temperature at which 50% of the protein is unfolded. Binding of a compound to the protein can alter protein’s stability: the Tm increases if the compound stabilizes the protein.

**Figure 5:**
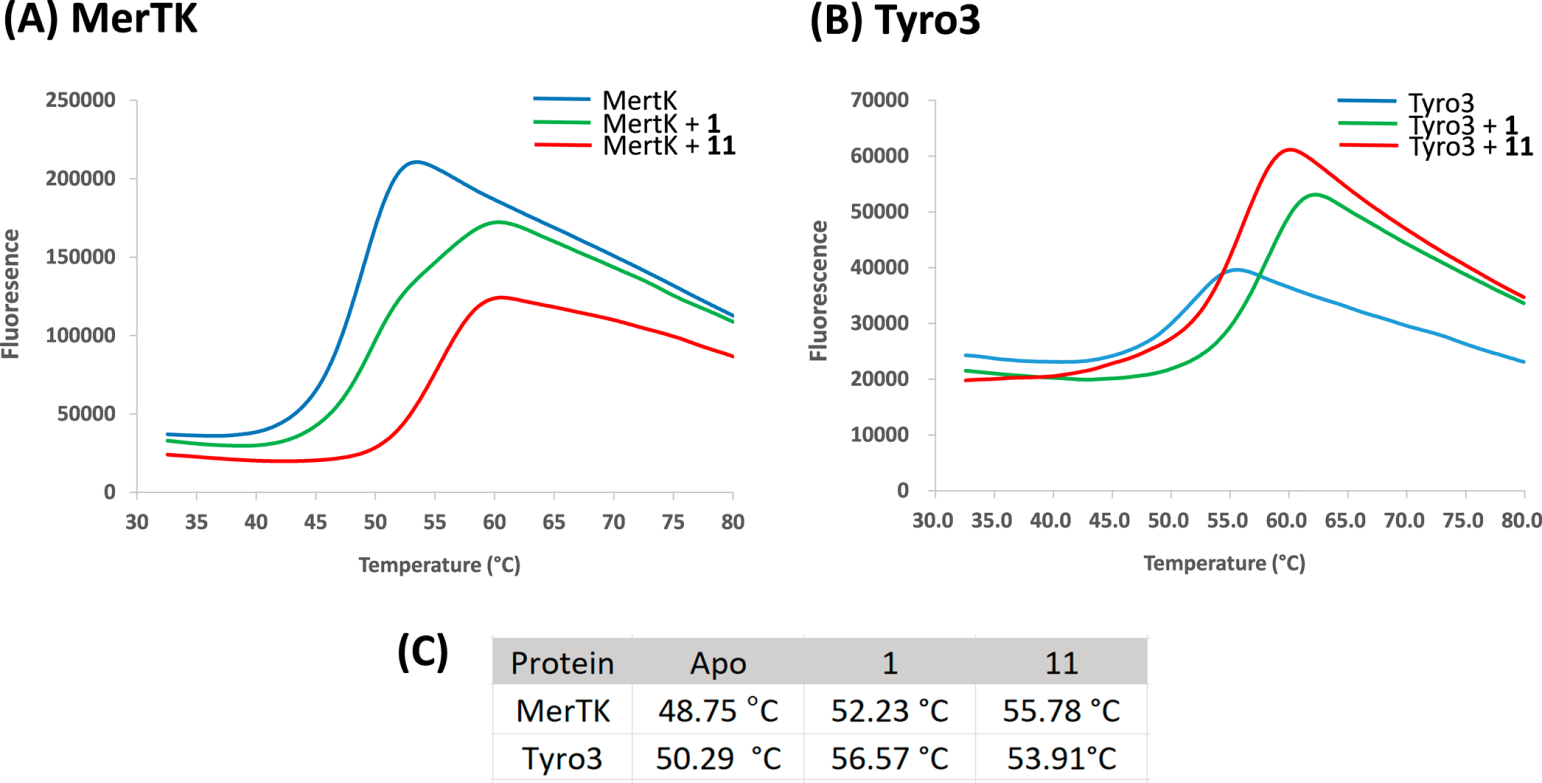
Thermal shift assay results assessing the interaction of compounds **1** and **11** with (A) MerTK and (B)­Tyro3 kinase domains, performed at a compound concentration of 2.5 μM. Fluorescence units (RFU) are plotted against temperature, with protein melting temperatures determined as the midpoint of the thermal transition. (C) Average protein melting temperatures, calculated from triplicate measurements.

In the absence of inhibitors, MerTK shows Tm of 48.75 °C. Upon binding with inhibitors, **11** and **1** at 2.5 uM, the Tm values increase to 55.78 and 52.23 °C, respectively (Figure 5A,C). **11** shows a larger Tm shift (ΔTm = 7.03 °C) compared to **1 (**ΔTm = 3.48 °C), indicating stronger stabilization of MerTK by **11**. This result is consistent with observations from structural biology where the protein structure becomes more rigid, particularly around the αC helix and its surrounding regions, upon binding with **11.**

In addition to MerTK, a thermal shift assay (TSA) was also conducted to assess the stability of Tyro3 upon binding with **11** and **1**. In the absence of inhibitors, Tyro3 shows Tm of 50.29 °C (Figure 5B,C). In the presence of **1** (2.5 μM), both proteins exhibit increased melting temperatures, suggesting **1** enhances stability. Notably, a larger shift in melting temperature (ΔTm = 6.28 °C) was observed for Tyro3 compared to MerTK (ΔTm = 3.48 °C), suggesting that **1** stabilizes Tyro3 more significantly than MerTK. Conversely, **11** (2.5 μM) induces greater ΔTm shifts for MerTK (ΔTm = 7.03 °C) than for Tyro 3 (ΔTm = 3.62 °C), indicating that **11** stabilizes MerTK more effectively than Tyro3.

To further investigate the binding of **11** and **1** to Tyro3, a docking study was performed with Tyro3 (Figure 6). When **1** is docked to the Tyro3 kinase domain, it adopts a downward conformation similar to that observed in the MerTK complex. This conformation aligns with the DFG motif and extends into the allosteric region near the αC helix, forming interactions within the Tyro3 binding pocket that are comparable to those observed in the MerTK complex. These results suggest that **1** exhibits similar binding to both Tyro3 and MerTK (Figure 6A). In contrast, when compound **11** is docked to Tyro3, the 1-methylpyrazol-3-one ring of compound **11** rotates away from the DFG motif, and the fluorobenzene headgroup shifts away from αC helix, failing to properly stabilize it (Figure 6B). Consequently, the head region becomes exposed to a solvent-accessible area, resulting in the loss of important hydrophobic and C–F···O interactions with the αC helix and adjacent residues with Tyro3. This disruption leads to a lower docking score for **11**. These findings are consistent with thermal shift data, where **11** (ΔTm = 3.62 °C) is less effective at stabilizing Tyro3 compared to its effect on MerTK (ΔTm = 7.03 °C).

**Figure 6:**
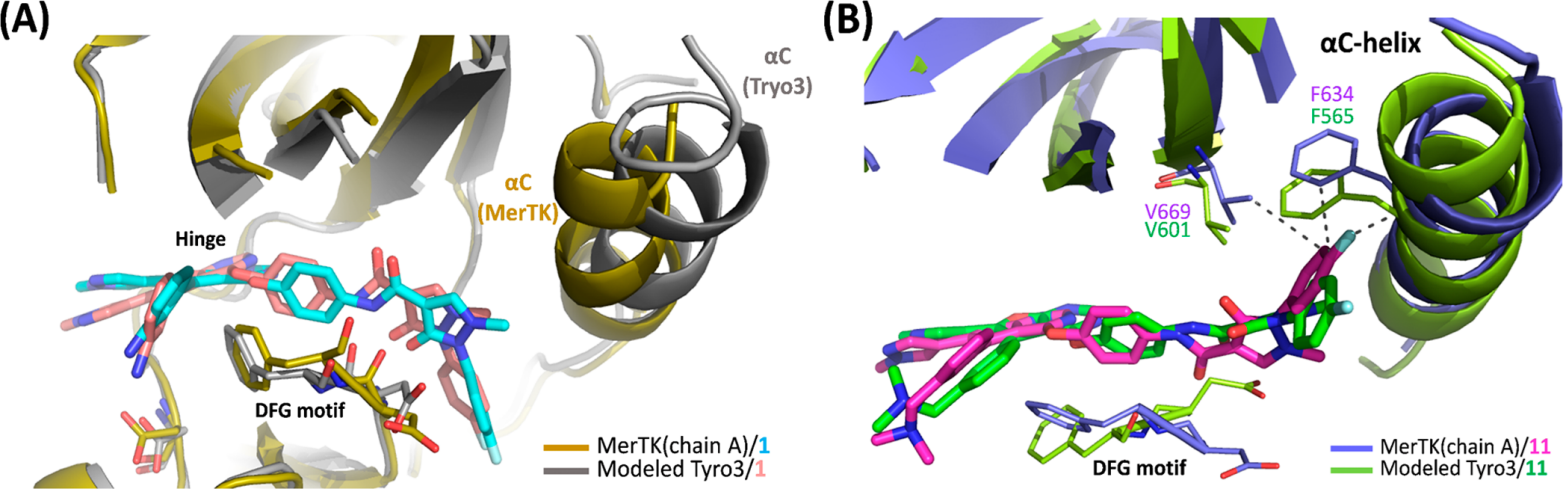
(A) Superimposition of MerTK/**1** (PDB: 9KRY) with the modeled Tyro3/ **1** shows a similar docked pose of **1** in both MerTK and Tyro3. (B) Superimposition of MerTK/**11** (PDB: 9KRZ) with the modeled Tyro3/**11** reveals that the docked pose of **11** in Tyro3 loses interactions with the αC helix and surrounding residues. Residue numbers are shown for MerTK (purple) and Tyro3 (green).

As revealed by structural biology studies, thermal shift experiments, and docking analysis, compound **11** adopts an upward orientation and forms extensive interactions with the αC helix in MerTK. The structural biology data also demonstrate that **11** stabilizes the αC helix in an outward conformation across both monomers of MerTK. This stabilization is supported by the thermal shift data, where **11** significantly enhances MerTK stability, yielding a ΔTm value of 7.03 °C. In contrast, as revealed in the docking study of Tyro3, the headgroup of **11** is distanced from the αC helix of Tyro3, preventing stabilization of the αC helix as observed in MerTK. This is reflected in the thermal shift data, where **11** is less effective at stabilizing Tyro3, with a lower ΔTm value of 3.62 °C.

The differential stabilization of the αC helix by **11** in MerTK versus Tyro3 is consistent with their respective inhibitory effects, with IC_50_ values of 21.5 nM for MerTK and 991.3 nM for Tyro3. These findings suggest that the selectivity of **11** for MerTK over Tyro3 is attributed to its interactions with and stabilization of the αC helix of MerTK.

### Pharmacokinetic Study

Due to its potent inhibition toward MerTK and high selectivity over Tyro 3, **11** emerges as a promising compound for further pharmacokinetic study and in vivo efficacy study.

The pharmacokinetic properties of **11** revealed that after administering **11** intravenously resulted in large volume of distribution (Vss = 4.9 L/kg), moderate half-life (*t*_1/2_ = 5.3h), and low plasma clearance rate (CL = 19.4 mL/min/kg). Oral administration of **11** also showed moderate half-life (t1/2 = 3.1 h), high Cmax (2867 ng/mL) and AUC (4335 ng/mL·h) with 44% oral bioavailability (Table 4). These findings suggest that **11** possesses a favorable pharmacokinetic profile in mice, warranting further investigation for therapeutic potential.

**Table 4:** *In Vivo* Pharmacokinetic Parameters of **11**

I.V. (dose: 2mg/kg)					P.O. (dose: 10mg/kg)					
Compound	*T*_1/2_(hr)	CL(mL/min/kg)	Vss(L/kg)	AUC (0-inf) (ng/mL x hr)	*T*_1/2_(hr)	*C*_max_(ng/mL)	*T*_max_(hr)	AUC (0-inf) (ng/mL x hr)	*F*(%)	Microsomal Stability @ 30 min
**11**	5.3	19.4	4.9	1,951	3.1	2,867	1.0	4,335	44	50.40%

### Anti-Tumor Efficacy and Immunomodulatory Activities of 11

A murine colon tumor MC38 syngeneic model was used to evaluate the antitumor efficacy and the immunomodulatory activity of **11**. Our data showed that oral administration of **11** at 50 mg/kg twice a day, 5 days a week for 3 weeks delayed tumor growth and produced a TGI value of 54.8 ± 8.0% vs vehicle-treated group (Figure 7A) without significant body weight loss and no obvious changes in the level of liver and kidney serum biochemistry markers (Figure 7B). To determine the effect of **11** on tumor immune microenvironment, MC38 tumor-bearing mice were treated with either vehicle control or 50 mg/kg of **11** twice a day, 5 days a week for 2 weeks. As seen in Figure 7C, treatment with **11** did not alter total intratumoral macrophage (CD45^+^Cd11b^+^F4/80^+^) percentage compared with vehicle-treated group. Further analysis revealed that **11** increased intratumoral M1-like antitumor macrophages (CD45^+^ Cd11b^+^F4/80^+^ Cd86^+^) by 30% and reduced intratumoral M2-like pro-tumor-macrophages (CD45^+^ Cd11b^+^F4/80^+^Cd206^+^) by 44%, resulting in a 2-fold increase in the ratio of M1/M2 within the tumor (Figure 7C). This modulation of the tumor microenvironment by **11** was further revealed by a 2.6-fold increase in the ratio of cytotoxic CD8+ T-cells to M2 within the tumor (Figure 7C). In the spleen, treatment with **11** did not change the percentage of Cd3^+^ pan-T cell, Cd4^+^ helper T cell populations, and Cd8^+^ cytotoxic T cell populations; however, the Cd4^+^ MERTK^+^ and Cd8^+^ MERTK^+^ cell populations were significantly reduced by **11** in the spleen (Figure 7C). Collectively, the above findings demonstrated that **11** is a potent immunomodulatory agent, with its antitumor efficacy mediated through the remodeling of the tumor immune microenvironment.

**Figure 7:**
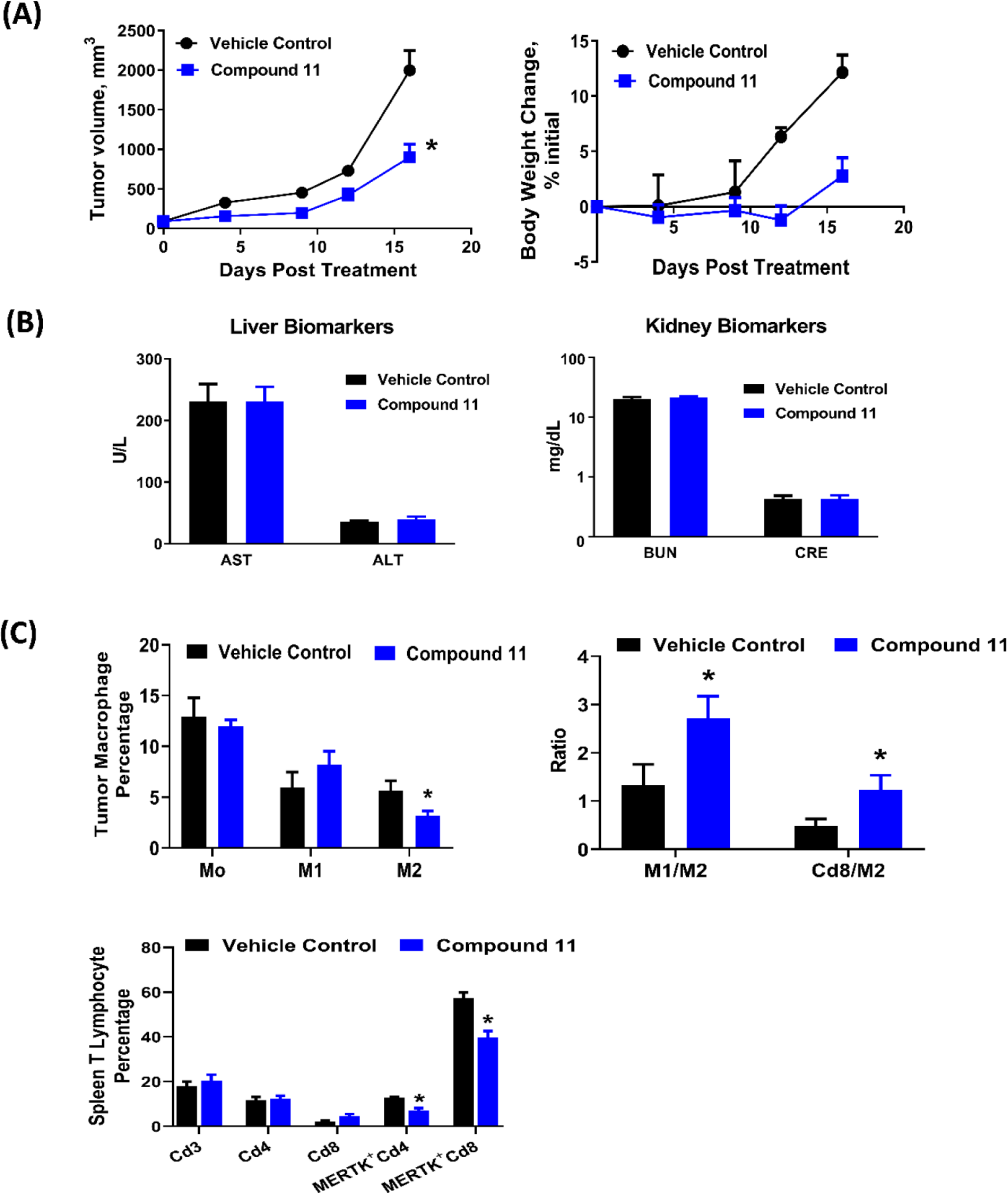
Antitumor efficacy and immunomodulatory activities of **11** *in vivo*. MC38 murine colon tumor-bearing mice were dosed orally with **11** at 50 mg/kg twice a day, 5 days a week for 3 weeks. Blood samples were collected for liver and kidney biomarkers analyses. (A) Tumor volume and body weight changes. (B) Liver biomarkers (alanine transaminase, ALT, and aspartate transaminase, AST) and kidney biomarkers (blood urea nitrogen, BUN, and creatinine, CRE. (C) Immunophenotyping. MC38 tumor–bearing mice were treated with oral 50 mg/kg **11** twice a day, 5 days a week for 2 weeks. Tumor and spleen tissues were then harvested and isolated into single cells. The immune cells were analyzed by flow cytometry analyses. Data were expressed as mean ± SEM, *n* = 8 mice per group for antitumor efficacy study and biomarker analyses; *n* = 5 mice per group for immunophenotyping experiment. *p < 0.05 vs vehicle control. Error bars in some data points are smaller than the symbols.

## Conclusion

Achieving selectivity in kinase inhibition remains a significant challenge due to the high structural conservation and conformational similarities across human kinases. The αC helix plays an important role in regulating kinase activity, and its conformation is closely linked to the kinase’s function. Modulating the αC helix conformation can, therefore, serve as a strategy for regulating kinase function and enhancing the selectivity of inhibitors.

In this study, we present a novel approach for designing MerTK-selective inhibitors by exploiting the conformational dynamics of the αC helix of MerTK and promoting its repositioning. Our findings reveal that the αC helix of MerTK adopts two distinct conformations within the kinase dimer upon binding to a pan-TAM inhibitor: monomer A assumes an αC-in-like conformation, while monomer B adopts an αC-out conformation. This dual conformation within a single kinase dimer represents a unique and rarely observed structural feature in kinase.

By systematically modifying the head and tail moieties of pan-TAM inhibitors to specifically target the αC helix, we identified a potent and selective MerTK inhibitor, **11**, which stabilizes the αC helix in its αC-out conformation, thereby reinforcing the structural integrity of the kinase. Crystallographic analysis confirmed that **11** induces displacement of the αC helix and stabilizes it in the αC-out conformation in both monomers.

In active kinases, the αC helix adopts an “in” conformation where it is tightly packed against the rest of the kinase domain. This conformation is critical for kinase activation, as the conserved Glu in the αC helix forms a salt bridge with a Lys on the β-strand and interacts with the A-loop. Displacement of the αC helix from the’in’ to’out’ conformation disrupts these key interactions, resulting in a loss of kinase activity and a transition to an inactive state. Studies have shown that the displacement of the αC helix is a crucial determinant in the energy barrier for activation and is essential for controlling the transition between active and inactive states.^33^

In the structure of MerTK in complex with compound **1**, the αC helix adopts two distinct conformations within the kinase dimer: monomer A assumes an’αC-in’ conformation, while monomer B adopts an’αC-out’ conformation. In contrast, in the structure of MerTK in complex with compound **11**, compound **11** induces a more significant displacement of the αC helix, stabilizing it in the’αC-out’ conformation in both monomers. This suggests that compound **11** induces a more complete switch to an inactive state as compared with compound **1**.

Thermal shift assays further validated these findings, showing that **11** significantly stabilizes MerTK while exhibiting reduced stabilization of Tyro3. Docking studies revealed that the extended tail moiety of **11** weakens Tyro3 binding, explaining its high selectivity for MerTK.

Moreover, pharmacokinetic studies and *in vivo* experiments demonstrated that **11** exhibits a favorable pharmacokinetic profile and shows potent antitumor efficacy in the MC38 syngeneic mouse model, mediated by remodeling of the tumor immune microenvironment.

In conclusion, this study demonstrates the potential of modulating kinase conformations, particularly through αC helix repositioning within dynamic kinase dimers, to enhance inhibitor selectivity. Type I kinase inhibitors typically bind to the active DFG-in conformation of kinases, competitively inhibiting ATP by interacting with residues in the ATP-binding pocket or hinge region. In contrast, Type II inhibitors bind in the DFG-out conformation, stabilizing the kinase in an inactive state.^34^ While Type I inhibitors often lack selectivity due to the high conservation of the ATP-binding pocket structure among kinases in the active state, Type II inhibitors, although initially thought to offer better selectivity, can encounter similar challenges, as the DFG-out conformation is often observed in many kinases.^28^ Our approach targets the αC helix, whose conformation is more dynamic and less conserved across kinases. By stabilizing the αC-out conformation, our inhibitors exploit the conformational flexibility of the αC helix, providing a more kinase-specific and selective mechanism of inhibition. In our study, compound **1**, a Type II inhibitor, showed pan-activity against the TAM family, while compound **11**, which specifically targets the αC helix in its αC-out conformation, proved to be a potent and selective MerTK inhibitor. Compared to Type I and Type II kinase inhibitors, our approach offers an advantage in enhancing selectivity by exploiting the dynamic αC helix conformational switch. This strategy allows for better targeting of specific kinases and offers a promising alternative for the development of selective kinase inhibitors.

By integrating structural biology, medicinal chemistry, and comprehensive in vitro and *in vivo* studies, compound **11** emerges as a promising candidate for further development. Our findings not only advance the understanding of MerTK-specific inhibition but also offer an effective strategy for designing selective kinase inhibitors targeting the αC helix conformation.

## Experimental Section

### General Methods for Chemistry

Reagent-grade chemicals and solvents obtained from commercial sources were used as received, unless otherwise specified. Reactions were performed under an inert atmosphere of dry nitrogen or argon. Reaction progress was monitored by thin-layer chromatography (TLC) on Merck 60 F254 silica gel plates (glass- or aluminum-backed). Visualization was achieved under UV light (254 nm) or by staining with potassium permanganate solution (Aldrich), followed by heating at 80 °C. Flash column chromatography was conducted using silica gel (Merck grade 9385, 230–400 mesh or Silicycle SiliaFlash P60, R12030B, 230–400 mesh). ^1^H and ^13^C NMR spectra were obtained with a Bruker AVANCE III 400 or 600 MHz spectrometers. Data were processed using Mnova software (Mestrelab Research). Chemical shift (δ) are reported in parts per million (ppm) relative to residual solvent peaks: DMSO-*d*_6_ (δ_H_ = 2.50 ppm, δ_C_ = 39.5 ppm), CDCl_3_ (δ_H_ = 7.26 ppm, δ_C_ = 77.0 ppm). Singal multiplicities are abbreviated as follows: s = singlet, d = doublet, q = quartet, dd = doublet of doublets, ddd = doublet of doublets of doublets, dddd = doublet of doublets of doublets of doublets, *m* = multiplet. Coupling constants (*J*) are reported in Hertz (Hz). Low-resolution mass spectrometry (LRMS) data were obtained using either Agilent MSD-1100 ESI-MS/MS system or Agilent Infinity II 1290 LC/MS system with electrospray ionization (ESI). High-resolution mass spectrometry (HRMS) data were obtained using a Varian 901-MS FT-ICR HPLC/MS-MS instrument. Compound purity was assessed by high-performance liquid chromatography (HPLC) or ultraperformance liquid chromatography (UPLC). HPLC analysis was performed using a Hitachi 2000 series instrument equipped with an Agilent ZORBAX Eclipse XDB-C18 column (5 μm, 4.6 × 150 mm) at 25 °C. The mobile phase consisted of acetonitrile (A) and water containing 0.1% formic acid and 2 mM ammonium acetate (B). The gradient program was as follows: 0.0 min, A/B = 10:90 (0.5 mL/min); 25.0 min, A/B = 90:10 (0.5 mL/min); 30.5 min, A/B = 10:90 (1.0 mL/min); 34.5 min, A/B = 10:90 (0.5 mL/min); 37.0 min, A/B = 10:90 (0.5 mL/min). The injection volume was 20 μL. UPLC analysis was carried out using a Waters Acquity UPLC/BSM syntem equipped with a Waters BEH-C18 column (1.7 μm, 2.1 × 50 mm) at 25 °C. The same mobile phases were used as in HPLC. The gradient was as follows: 0.00 min, A/B = 10:90; 4.15 min, A/B = 90:10; 5.00 min, A/B = 10:90; 6.50 min, A/B = 10:90. The flow rate was 0.6 mL/min, and the injection volume was 5 μL. Detection was performed at 254 nm. All compounds are >95% by HPLC or UPLC analysis except for compounds **3** (89.9%), **7** (93.0%), **8** (92.3%), **13** (94.3%), **14** (94.5%), **16** (94.1%). IUPAC nomenclature of all compounds was assigned using Mnova software (Mestrelab Research).

#### *N*-(4-{[5-(3-Aminophenyl)-6-(1-methyl-1*H*-pyrazol-4-yl)­furo­[2,3-*d*]­pyrimidin-4-yl]­oxy}­phenyl)-2-(4-fluorophenyl)-1-methyl-3-oxo-2,3-dihydro-1*H*-pyrazole-4-carboxamide *(* **1** *)*

To a stirred solution of compound **23a** (115 mg, 0.19 mmol, 1.0 equiv) in 1,4-dioxane (1.2 mL) were added (3-aminophenyl)­boronic acid (34 mg, 0.25 mmol, 1.3 equiv), Pd­(dppf)­Cl_2_ (42 mg, 0.06 mmol, 30 mol %), and aqueous Na_2_CO_3_ (2M, 121 mg, 1.14 mmol, 6.0 equiv). The resulting mixture was degassed for 30 min and backfilled with Argon_(g)_. After stirring for 3 h at 100 °C, the reaction mixture was allowed to cool to room temperature. It was then filtered through a pad of Celite, diluted with 10 mL of water, washed with 10 mL of saturated aqueous NaHCO_3_, and extracted with three 10 mL portions of dichloromethane. The combined organic extracts were washed with brine, dried over anhydrous MgSO_4_, filtered, and concentrated *in vacuo*. The crude residue was purified by automated flash column chromatography (Combiflash, 2% methanol in dichloromethane), followed by preparative thin-layer chromatography (6% methanol in dichloromethane) to yield the title compound **1** (18 mg, 0.03 mmol, 15%) as a yellow solid. ^1^H NMR (400 MHz, DMSO-*d*_*6*_) δ 10.34 (s, 1H), 8.59 (s, 1H), 8.45 (s, 1H), 7.63 (d, *J* = 8.8 Hz, 2H), 7.56 (dd, *J* = 8.8, 4.8 Hz, 2H), 7.52 (s, 1H), 7.44 (dd, *J* = 8.8, 8.8 Hz, 2H), 7.17 (d, *J* = 8.8 Hz, 2H), 7.13 (dd, *J* = 8.0, 7.6 Hz, 1H), 6.82 (dd, *J* = 2.2, 2.0 Hz, 1H), 6.75 (d, *J* = 7.6 Hz, 1H), 6.62 (dd, *J* = 8.0, 2.2 Hz, 1H), 5.20 (s, 2H), 3.86 (s, 3H), 3.47 (s, 3H). ^13^C NMR (101 MHz, DMSO-*d*_*6*_) δ 166.69, 162.58, 162.15 (d, *J*_C–F_ = 248.1 Hz), 162.12, 159.92, 151.84, 148.65, 147.50, 144.31, 141.33, 136.63, 136.05, 130.90, 130.47 (d, *J*_C–F_ = 9.1 Hz), 129.40, 128.93, 128.39 (d, *J*_C–F_ = 2.9 Hz), 122.31, 119.94, 117.43, 116.48 (d, *J*_C–F_ = 23.2 Hz), 115.39, 113.80, 112.69, 110.95, 106.21, 99.69, 38.75, 36.56. LRMS (ESI) *m*/*z* 617.3 [M + H]^+^. HRMS (ESI) *m*/*z* for C_33_H_26_FN_8_O_4_ [M + H]^+^, calcd 617.2061, found 617.2061. HPLC purity 100.00% (*t*_R_ = 18.39 min).

#### *N*-(4-{[5-(3-Aminophenyl)-6-(1-methyl-1H-pyrazol-4-yl)­furo­[2,3-*d*]­pyrimidin-4-yl]­oxy}­phenyl)-2-(3-fluorophenyl)-1-methyl-3-oxo-2,3-dihydro-1*H*-pyrazole-4-carboxamide (**2**)

To a stirred solution of compound **23b** (106 mg, 0.18 mmol, 1.0 equiv) in tetrahydrofuran (1.8 mL) and *N*,*N*-dimethylformamide (1.8 mL) were added (3-aminophenyl)­boronic acid (31 mg, 0.23 mmol, 1.3 equiv), Pd­(dppf)­Cl_2_ (42 mg, 0.06 mmol, 30 mol %), and aqueous Na_2_CO_3_ (2M, 112 mg, 1.06 mmol, 6.0 equiv). The resulting mixture was degassed for 30 min and backfilled with Argon_(g)._ After stirring for 16 h at 80 °C, the reaction mixture was allowed to cool to room temperature. It was then filtered through a pad of Celite, diluted with 10 mL of water, washed with 10 mL of saturated aqueous NaHCO_3_, and extracted with three 10 mL portions of dichloromethane. The combined organic extracts were washed with brine, dried over anhydrous MgSO_4_, filtered, and concentrated *in vacuo*. The crude residue was purified by automated flash column chromatography (Combiflash, 1–5% methanol in dichloromethane) followed by preparative thin-layer chromatography (6% methanol in dichloromethane) to yield the title compound **2** (41 mg, 0.07 mmol, 38%) as a yellow solid. ^1^H NMR (600 MHz, DMSO-*d*_*6*_) δ 10.28 (s, 1H), 8.64 (s, 1H), 8.45 (s, 1H), 7.66–7.62 (m, 3H), 7.52 (s, 1H), 7.46 (ddd, *J* = 9.6, 2.4, 1.8 Hz, 1H), 7.40 (dddd, *J* = 9.0, 9.0, 2.4, 0.9 Hz, 1H), 7.35 (ddd, *J* = 7.8, 1.8, 0.9 Hz, 1H), 7.18 (d, *J* = 9.0 Hz, 2H), 7.13 (dd, *J* = 7.8, 7.5 Hz, 1H), 6.82 (dd, *J* = 2.4, 1.8 Hz, 1H), 6.75 (ddd, *J* = 7.5, 2.1, 1.8 Hz, 1H), 6.62 (ddd, *J* = 7.8, 2.4, 2.1 Hz, 1H), 5.20 (s, 2H), 3.87 (s, 3H), 3.51 (s, 3H). ^13^C NMR (151 MHz, DMSO-*d*_*6*_) δ 166.69, 162.58, 162.17, 162.01 (d, *J*_C–F_ = 245.8 Hz), 159.75, 151.83, 148.65, 147.56, 144.32, 142.70, 136.63, 135.97, 133.57 (d, *J*_C–F_ = 10.6 Hz), 131.09 (d, *J*_C–F_ = 8.9 Hz), 130.90, 129.40, 128.93, 123.52 (d, *J*_C–F_ = 3.0 Hz), 122.31, 120.01, 117.43, 116.10 (d, *J*_C–F_ = 20.8 Hz), 115.39, 114.72 (d, *J*_C–F_ = 24.0 Hz), 113.80, 112.69, 110.96, 106.22, 100.04, 38.75, 36.91. LRMS (ESI) *m*/*z* 617.1 [M + H]^+^. HRMS (ESI) *m*/*z* for C_33_H_25_FN_8_NaO_4_ [M + Na]^+^, calcd 639.1881, found 639.1880. UPLC purity 97.82% (*t*_R_ = 2.087 min).

#### *N*-(4-{[5-(3-Aminophenyl)-6-(1-methyl-1*H*-pyrazol-4-yl)­furo­[2,3-*d*]­pyrimidin-4-yl]­oxy}­phenyl)-1-methyl-2-(4-methylphenyl)-3-oxo-2,3-dihydro-1*H*-pyrazole-4-carboxamide (**3**)

To a stirred solution of compound **23c** (120 mg, 0.20 mmol, 1.0 equiv) in tetrahydrofuran (2.0 mL) and *N*,*N*-dimethylformamide (2.0 mL) were added (3-aminophenyl)­boronic acid (36 mg, 0.26 mmol, 1.3 equiv), Pd­(dppf)­Cl_2_ (44 mg, 0.06 mmol, 30 mol %), and aqueous Na_2_CO_3_ (2M,127 mg, 1.20 mmol, 6.0 equiv). The resulting mixture was degassed for 30 min and backfilled with Argon_(g)_. After stirring for 16 h at 80 °C, the reaction mixture was allowed to cool to room temperature. It was then filtered through a pad of Celite, diluted with 10 mL of water, washed with 10 mL of saturated aqueous NaHCO_3_, and extracted with three 10 mL portions of dichloromethane. The combined organic extracts were washed with brine, dried over anhydrous MgSO_4_, filtered, and concentrated *in vacuo*. The crude residue was purified by Combiflash automated flash column chromatography (Combiflash, 0–20% methanol in dichloromethane) to yield the title compound **3** (51 mg, 0.08 mmol, 42%) as a yellow solid. ^1^H NMR (600 MHz, DMSO-*d*_*6*_) δ 10.39 (s, 1H), 8.57 (s, 1H), 8.45 (s, 1H), 8.04 (s, 1H), 7.63 (d, *J* = 9.0 Hz, 2H), 7.52 (s, 1H), 7.39 (d, *J* = 8.7 Hz, 2H), 7.35 (d, *J* = 8.7 Hz, 2H), 7.17 (d, *J* = 9.0 Hz, 2H), 7.13 (dd, *J* = 8.1, 7.5 Hz, 1H), 6.82 (dd, *J* = 2.4, 1.8 Hz, 1H), 6.75 (ddd, *J* = 7.5, 1.8, 0.9 Hz, 1H), 6.62 (ddd, *J* = 8.1, 2.4, 0.9 Hz, 1H), 5.20 (s, 2H), 3.87 (s, 3H), 3.45 (s, 3H), 2.40 (s, 3H). ^13^C NMR (151 MHz, DMSO-*d*_*6*_) δ 166.69, 162.58, 162.02, 160.00, 151.84, 148.65, 147.47, 144.31, 140.98, 139.23, 136.62, 136.08, 130.89, 129.96, 129.53, 129.40, 128.92, 127.81, 122.30, 119.92, 117.41, 115.38, 113.79, 112.69, 110.95, 106.21, 99.73, 38.75, 36.54, 20.75. LRMS (ESI) *m*/*z* 613.2 [M + H]^+^. HRMS (ESI) *m*/*z* for C_34_H_28_N_8_NaO_4_ [M + Na]^+^, calcd 635.2131, found 635.2130. UPLC purity 89.85% (*t*_R_ = 2.143 min).

#### *N*-(4-{[5-(3-Aminophenyl)-6-(1-methyl-1*H*-pyrazol-4-yl)­furo­[5,4-*d*]­pyrimidin-4-yl]­oxy}­phenyl)-1-methyl-3-oxo-2-[4-(trifluoromethyl)­phenyl]-2,3-dihydro-1*H*-pyrazole-4-carboxamide (**4**)

To a stirred solution of compound **23d** (31 mg, 0.05 mmol, 1.0 equiv) in *N*,*N*-dimethylformamide (1.0 mL) were added (3-aminophenyl)­boronic acid (8 mg, 0.06 mmol, 1.2 equiv), Pd­(dppf)­Cl_2_ (3 mg, 0.004 mmol, 9 mol %), and aqueous Na_2_CO_3_(2M, 15 mg, 0.14 mmol, 3.0 equiv). The resulting mixture was degassed for 30 min and backfilled with Argon_(g)_. After stirring for 2 h at 80 °C, the reaction mixture was allowed to cool to room temperature. It was then filtered through a pad of Celite, diluted with 10 mL of water, washed with 10 mL of saturated aqueous NaHCO_3_, and extracted with three 10 mL portions of dichloromethane. The combined organic extracts were washed with brine, dried over anhydrous MgSO_4_, filtered, and concentrated *in vacuo*. The crude residue was purified by preparative thin-layer chromatography (5% methanol in dichloromethane) to yield the title compound **4** (18 mg, 0.03 mmol, 57%) as a yellow solid. ^1^H NMR (400 MHz, DMSO-*d*_*6*_) δ 10.22 (s, 1H), 8.70 (s, 1H), 8.45 (s, 1H), 8.04 (s, 1H), 7.97 (d, *J* = 8.2 Hz, 2H), 7.73 (d, *J* = 8.2 Hz, 2H), 7.65 (d, *J* = 8.8 Hz, 2H), 7.52 (d, *J* = 0.4 Hz, 1H), 7.18 (d, *J* = 8.8 Hz, 2H), 7.13 (dd, *J* = 8.0, 7.6 Hz, 1H), 6.82 (dd, *J* = 2.2, 1.4 Hz, 1H), 6.75 (ddd, *J* = 7.6, 1.4, 1.0 Hz, 1H), 6.62 (ddd, *J* = 8.0, 2.2, 1.0 Hz, 1H), 5.21 (s, 2H), 3.87 (s, 3H), 3.53 (s, 3H). ^13^C NMR (101 MHz, DMSO-*d*_*6*_) δ 166.69, 162.57, 162.32, 159.59, 151.84, 148.64, 147.61, 144.32, 144.10, 136.63, 135.89, 135.84, 130.89, 129.41, 128.93, 128.70 (q, *J*_C–F_ = 32.32 Hz), 127.30, 126.53 (q, *J*_C–F_ = 3.94 Hz), 123.85 (q, *J*_C–F_ = 273.51 Hz), 122.32, 120.08, 117.42, 115.39, 113.80, 112.68, 110.94, 106.22, 100.44, 38.76, 37.22. LRMS (ESI) *m*/*z* 667.2 [M + H]^+^. HRMS (ESI) *m*/*z* for C_34_H_25_F_3_N_8_NaO_4_ [M + Na]^+^, calcd 689.1849, found 689.1849. UPLC purity 98.70% (*t*_R_ = 2.447 min).

#### *N*-(4-{[5-(3-Aminophenyl)-6-(1-methyl-1*H*-pyrazol-4-yl)­furo­[2,3-*d*]­pyrimidin-4-yl]­oxy}­phenyl)-2-(4-fluorophenyl)-1,5-dimethyl-3-oxo-2,3-dihydro-1*H*-pyrazole-4-carboxamide (**5**)

To a stirred solution of compound **23e** (84 mg, 0.14 mmol, 1.0 equiv) in tetrahydrofuran (2.0 mL) and *N*,*N*-dimethylformamide (1.5 mL) were added (3-aminophenyl)­boronic acid (24 mg, 0.18 mmol, 1.3 equiv), Pd­(dppf)­Cl_2_ (30 mg, 0.04 mmol, 30 mol %), and aqueous Na_2_CO_3_ (2M, 87 mg, 0.82 mmol, 6.0 equiv). The resulting mixture was degassed for 30 min and backfilled with Argon_(g)_. After stirring for 16 h at 90 °C, the reaction mixture was allowed to cool to room temperature. It was then filtered through a pad of Celite, diluted with 10 mL of water, washed with 10 mL of saturated aqueous NaHCO_3_, and extracted with three 10 mL portions of dichloromethane. The combined organic extracts were washed with brine, dried over anhydrous MgSO_4_, filtered, and concentrated *in vacuo*. The crude residue was purified by automated flash column chromatography (Combiflash, 0–10% methanol in dichloromethane) to yield the title compound **5** (46 mg, 0.07 mmol, 54%) as a yellow solid. ^1^H NMR (400 MHz, DMSO-*d*_*6*_) δ 10.76 (s, 1H), 8.45 (s, 1H), 8.04 (s, 1H), 7.62 (d, *J* = 9.2 Hz, 2H), 7.52 (d, *J* = 0.8 Hz, 1H), 7.51 (dd, *J* = 9.0, 5.2 Hz, 2H), 7.43 (dd, *J* = 9.0, 8.8 Hz, 2H), 7.43 (dd, *J* = 9.0, 8.8 Hz, 2H), 7.16 (d, *J* = 9.2 Hz, 2H), 7.13 (dd, *J* = 8.4, 7.6 Hz, 1H), 6.81 (dd, *J* = 2.2, 1.8 Hz, 1H), 6.74 (ddd, *J* = 7.6, 1.8, 1.2 Hz, 1H), 6.62 (ddd, *J* = 8.4, 2.2, 1.2 Hz, 1H), 5.20 (s, 2H), 3.87 (s, 3H), 3.34 (s, 3H), 2.69 (s, 3H). ^13^C NMR (101 MHz, DMSO-*d*_*6*_) δ 166.68, 163.03, 162.60, 161.87 (d, *J*_C–F_ = 247.6 Hz), 161.11, 153.38, 151.85, 148.66, 147.36, 144.30, 136.62, 136.24, 130.89, 129.89 (d, *J*_C–F_ = 13.6 Hz), 129.40, 129.23 (d, *J*_C–F_ = 2.9 Hz), 128.93, 122.27, 119.88, 117.41, 116.44 (d, *J*_C–F_ = 23.1 Hz), 115.38, 113.79, 112.70, 110.95, 106.21, 96.86, 38.76, 33.07, 11.39. LRMS (ESI) *m*/*z* 631.2 [M + H]^+^. HRMS (ESI) *m*/*z* for C_34_H_27_FN_8_NaO_4_ [M + Na]^+^, calcd 653.2037, found 653.2044. HPLC purity 95.16% (*t*_R_ = 20.02 min).

#### *N*-(4-{[5-(3-Aminophenyl)-6-(1-methyl-1*H*-pyrazol-4-yl)­furo­[2,3-*d*]­pyrimidin-4-yl]­oxy}­phenyl)-1-(4-fluorophenyl)-6-methyl-2-oxo-1,2-dihydropyridine-3-carboxamide (**7**)

To a stirred solution of compound **25a** (310 mg, 0.50 mmol, 1.0 equiv) in tetrahydrofuran (5.0 mL) and *N*,*N*-dimethylformamide (5.0 mL) were added (3-aminophenyl)­boronic acid (103 mg, 0.75 mmol, 1.5 equiv), Pd­(dppf)­Cl_2_ (111 mg, 0.15 mmol, 30 mol %), and aqueous Na_2_CO_3_ (2M, 212 mg, 2.00 mmol, 4.0 equiv). The resulting mixture was degassed for 30 min and backfilled with Argon_(g)_. After stirring for 16 h at 110 °C, the reaction mixture was allowed to cool to room temperature. It was then filtered through a pad of Celite, diluted with 10 mL of water, washed with 10 mL of saturated aqueous NaHCO_3_, and extracted with three 10 mL portions of dichloromethane. The combined organic extracts were washed with brine, dried over anhydrous MgSO_4_, filtered, and concentrated *in vacuo*. The crude residue was purified by flash column chromatography (2% methanol in dichloromethane) to yield the title compound **7** (178 mg, 0.28 mmol, 56%) as a white solid. ^1^H NMR (600 MHz, DMSO-*d*_*6*_) δ 11.90 (s, 1H), 8.48 (d, *J* = 7.8 Hz, 1H), 8.44 (s, 1H), 8.03 (s, 1H), 7.72 (d, *J* = 9.0 Hz, 2H), 7.52 (s, 1H), 7.49 (dd, *J* = 9.0, 4.8 Hz, 2H), 7.43 (dd, *J* = 9.0, 8.4 Hz, 2H), 7.18 (d, *J* = 9.0 Hz, 2H), 7.12 (dd, *J* = 8.1, 7.8 Hz, 1H), 6.82 (dd, *J* = 2.4, 1.8 Hz, 1H), 6.74 (ddd, *J* = 7.8, 1.8, 1.2 Hz, 1H), 6.68 (d, *J* = 7.8 Hz, 1H), 6.61 (ddd, *J* = 8.1, 2.4, 1.2 Hz, 1H), 5.20 (s, 2H), 3.86 (s, 3H), 2.06 (s, 3H). ^13^C NMR (151 MHz, DMSO-*d*_*6*_) δ 166.69, 163.00, 162.78, 162.51, 161.96 (d, *J*_C–F_ = 246.1 Hz), 161.41, 153.06, 151.82, 148.65, 147.89, 144.32, 143.93, 136.62, 135.80, 134.17, 130.88, 130.09 (d, *J*_C–F_ = 8.9 Hz), 129.40, 128.92, 122.27, 120.59, 117.41, 117.26, 116.57 (d, *J*_C–F_ = 23.0 Hz), 115.38, 113.79, 112.68, 110.95, 107.72, 106.22, 38.75, 21.70. LRMS (ESI) *m*/*z*: 628.2 [M + H]^+^. HRMS (ESI) *m*/*z* for C_35_H_26_FN_7_NaO_4_ [M + Na]^+^, calcd 650.1928, found 650.1934. HPLC purity 92.97% (*t*_R_ = 22.97 min).

#### *N* ^1^-(4-{[5-(3-Aminophenyl)-6-(1-methyl-1*H*-pyrazol-4-yl)­furo­[2,3-*d*]­pyrimidin-4-yl]­oxy}­phenyl)-*N* ^1^-(4-fluorophenyl)­cyclopropane-1,1-dicarboxamide (**8**)

To a stirred solution of compound **35** (216 mg, 0.34 mmol, 1.0 equiv) in ethanol (8.0 mL) dichloromethane (8.0 mL) and water (1.6 mL) were added iron powder (114 mg, 2.04 mmol, 6.0 equiv) and saturated aqueous NH_4_Cl (820 μL). The resulting mixture was stirred for 10 h at 80 °C. After stirring, the reaction mixture was allowed to cool to room temperature, quenched with a solution of 6% NH_4_OH in methanol (1 mL). The reaction mixture was then filtered through a Celite pad and concentrated *in vacuo*. The crude residue was purified by automated flash column chromatography (Combiflash, 2% methanol in dichloromethane) to yield the title compound **8** (76 mg, 0.13 mmol, 37%) as a beige solid. ^1^H NMR (600 MHz, DMSO-*d*_*6*_) δ 10.10 (s, 1H), 8.44 (s, 1H), 8.04 (s, 1H), 7.66–7.60 (m, 4H), 7.52 (d, *J* = 0.6 Hz, 1H), 7.17–7.10 (m, 5H), 6.81 (dd, *J* = 2.4, 1.5 Hz, 1H), 6.74 (ddd, *J* = 7.8, 1.5, 1.2 Hz, 1H), 6.61 (dd, *J* = 8.4, 2.4, 1.2 Hz, 1H), 5.20 (s, 2H), 3.87 (s, 3H), 1.46 (s, 4H). ^13^C NMR (151 MHz, DMSO-*d*_*6*_) δ 168.12, 168.07, 166.69, 162.61, 158.23 (d, *J*_C–F_ = 239.8 Hz), 151.81, 148.65, 147.88, 144.32, 136.62, 136.22, 135.16, 130.89, 129.40, 128.92, 122.36 (d, *J*_C–F_ = 7.7 Hz), 121.80, 121.49, 117.40, 115.36, 114.99 (d, *J*_C–F_ = 22.3 Hz), 113.78, 112.67, 110.93, 106.20, 38.75, 31.40, 15.41. LRMS (ESI) *m*/*z* 604.3 [M + H]^+^. HRMS (ESI) *m*/*z* for C_33_H_26_FN_7_NaO_4_ [M + Na]^+^, calcd 626.1928, found 626.1924. HPLC purity 92.33% (*t*_R_ = 22.55 min).

#### *N*-(4-{[5-(3-Aminophenyl)-6-(1-methyl-1*H*-pyrazol-4-yl)­furo­[2,3-*d*]­pyrimidin-4-yl]­oxy}­phenyl)-1-methyl-2-oxo-1,2-dihydropyridine-3-carboxamide (**9**)

To a stirred solution of compound **25b** (110 mg, 0.21 mmol, 1.0 equiv) in 1,4-dioxane (1.2 mL) were added (3-aminophenyl)­boronic acid (38 mg, 0.28 mmol, 1.3 equiv), Pd­(dppf)­Cl_2_ (46 mg, 0.06 mmol, 30 mol %), and aqueous Na_2_CO_3_ (2M, 134 mg, 1.26 mmol, 6.1 equiv). The resulting mixture was degassed for 30 min and backfilled with Argon_(g)_. After stirring for 16 h at 80 °C, the reaction mixture was allowed to cool to room temperature. It was then filtered through a pad of Celite, diluted with 10 mL of water, washed with 10 mL of saturated aqueous NaHCO_3_, and extracted with three 10 mL portions of dichloromethane. The combined organic extracts were washed with brine, dried over anhydrous MgSO_4_, filtered, and concentrated *in vacuo*. The crude residue was and purified by automated flash column chromatography (Combiflash, 1–5% methanol in dichloromethane) to yield the title compound **9** (49 mg, 0.099 mmol, 44%) as a milky white solid. ^1^H NMR (600 MHz, DMSO-*d*_*6*_) δ 12.23 (s, 1H), 8.47–8.43 (m, 2H), 8.15 (dd, *J* = 6.6, 2.4 Hz, 1H), 8.04 (d, *J* = 0.6 Hz, 1H), 7.74 (d, *J* = 9.0 Hz, 2H), 7.53 (s, 1H), 7.21 (d, *J* = 9.0 Hz, 2H), 7.13 (dd, *J* = 8.4, 7.8 Hz, 1H), 6.82 (dd, *J* = 2.4, 1.8 Hz, 1H), 6.75 (ddd, *J* = 7.8, 1.8, 1.2 Hz, 1H), 6.64–6.58 (m, 2H), 5.21 (s, 2H), 3.87 (s, 3H), 3.63 (s, 3H). ^13^C NMR (151 MHz, DMSO-*d*_*6*_) δ 166.70, 162.52, 162.02, 161.43, 151.84, 148.66, 147.96, 144.49, 144.33 143.56, 136.63, 135.79, 130.89, 129.40, 128.93, 122.34, 120.64, 119.20, 117.42, 115.39, 113.80, 112.68, 110.95, 106.55, 106.24, 38.75, 37.90. LRMS (ESI) *m*/*z*: 556.2 [M + Na]^+^. HRMS (ESI) *m*/*z* for C_29_H_23_N_7_NaO_4_ [M + Na]^+^, calcd 556.1709, found 556.1716. HPLC purity 98.50% (*t*_R_ = 17.81 min).

#### *N*-{4-[(5-{3-[(Dimethylamino)­methyl]­phenyl}-6-(1-methyl-1*H*-pyrazol-4-yl)­furo­[2,3-*d*]­pyrimidin-4-yl)­oxy]­phenyl}-2-(4-fluorophenyl)-1-methyl-3-oxo-2,3-dihydro-1*H*-pyrazole-4-carboxamide (**11**)

To a stirred solution of compound **23a** (139 mg, 0.23 mmol, 1.0 equiv) in tetrahydrofuran (2.3 mL) and *N*,*N*-dimethylformamide (2.3 mL) were added *N*,*N*-dimethyl­[3-(4,4,5,5-tetramethyl-1,3,2-dioxaborolan-2-yl)­phenyl]­methanamine (90 mg, 0.34 mmol, 1.5 equiv), Pd­(dppf)­Cl_2_ (50 mg, 0.07 mmol, 30 mol %), and aqueous Na_2_CO_3_ (2M, 97 mg, 0.92 mmol, 4.0 equiv). The resulting mixture was degassed for 30 min and backfilled with Argon_(g)_. After stirring for 16 h at 110 °C, the reaction mixture was allowed to cool to room temperature. It was then filtered through a pad of Celite, diluted with 10 mL of water, washed with 10 mL of saturated aqueous NaHCO_3_, and extracted with three 10 mL portions of dichloromethane. The combined organic extracts were washed with brine, dried over anhydrous MgSO_4_, filtered, and concentrated *in vacuo*. The crude residue was purified by flash column chromatography (5–6% methanol in dichloromethane) to yield the title compound **11** (64 mg, 0.10 mmol, 42%) as a yellow solid. ^1^H NMR (600 MHz, DMSO-*d*_*6*_) δ 10.33 (s, 1H), 8.59 (s, 1H), 8.47 (s, 1H), 8.03 (s, 1H), 7.62 (d, *J* = 9.0 Hz, 2H), 7.59–7.52 (m, 4H), 7.50 (d, *J* = 0.6 Hz, 1H), 7.47–7.42 (m, 3H), 7.34 (d, *J* = 7.2 Hz, 1H), 7.15 (d, *J* = 9.0 Hz, 2H), 3.85 (s, 3H), 3.46 (s, 3H), 3.42 (s, 2H), 2.10 (s, 6H). ^13^C NMR (151 MHz, DMSO-*d*_*6*_) δ166.80, 162.72, 162.18 (d, *J*_C–F_ = 245.7 Hz), 162.14, 159.95, 152.03, 147.51, 144.56, 141.32, 136.57, 136.05, 130.69, 130.50 (d, *J*_C–F_ = 9.2 Hz), 130.27, 129.55, 128.83, 128.70, 128.41, 128.33, 122.24, 119.95, 116.52 (d, *J*_C–F_ = 22.8 Hz), 112.04, 110.75, 106.22, 99.68, 63.23, 44.94, 38.76, 36.59. LRMS (ESI) *m*/*z* 659.3 [M + H]^+^. HRMS (ESI) *m*/*z* for C_36_H_32_F_1_N_8_O_4_ [M + H]^+^, calcd 659.2531, found 659.2533. HPLC purity 99.20% (*t*_R_ = 14.50 min).

#### *N*-{4-[(5-{3-[(Dimethylamino)­methyl]­phenyl}-6-(1-methyl-1*H*-pyrazol-4-yl)­furo­[2,3-*d*]­pyrimidin-4-yl)­oxy]­phenyl}-2-(3-fluorophenyl)-1-methyl-3-oxo-2,3-dihydro-1*H*-pyrazole-4-carboxamide (**12**)

To a stirred solution of compound **23b** (158 mg, 0.26 mmol, 1.0 equiv) in tetrahydrofuran (2.6 mL) and *N*,*N*-dimethylformamide (2.6 mL) were added *N*,*N*-dimethyl­[3-(4,4,5,5-tetramethyl-1,3,2-dioxaborolan-2-yl)­phenyl]­methanamine hydrochloride (101 mg, 0.34 mmol, 1.3 equiv), Pd­(dppf)­Cl_2_ (42 mg, 0.06 mmol, 22 mol %), and aqueous Na_2_CO_3_ (2M, 166 mg, 1.57 mmol, 6.0 equiv). The resulting mixture was degassed for 30 min and backfilled with Argon_(g)_. After stirring for 16 h at 80 °C, the reaction mixture was allowed to cool to room temperature. It was then filtered through a pad of Celite, diluted with 10 mL of water, washed with 10 mL of saturated aqueous NaHCO_3_, and extracted with three 10 mL portions of dichloromethane. The combined organic extracts were washed with brine, dried over anhydrous MgSO_4_, filtered, and concentrated *in vacuo*. The crude residue was and purified by automated flash column chromatography (Combiflash, 1–10% methanol in dichloromethane) followed by preparative thin layer chromatography (10% methanol in dichloromethane) to yield the title compound **12** (56 mg, 0.09 mmol, 33%) as a brown solid. ^1^H NMR (600 MHz, DMSO-*d*_*6*_) δ 10.27 (s, 1H), 8.64 (s, 1H), 8.47 (s, 1H), 8.03 (s, 1H), 7.67–7.60 (m, 3H), 7.58 (s, 1H), 7.54 (d, *J* = 7.2 Hz, 1H), 7.51 (d, *J* = 2.4 Hz, 1H), 7.48–7.43 (m, 2H), 7.40 (dd, *J* = 9.6, 9.0 Hz, 1H), 7.37–7.31 (m, 2H), 7.15 (d, *J* = 9.0 Hz, 2H), 3.85 (s, 3H), 3.51 (s, 3H), 3.41 (s, 2H), 2.10 (s, 6H). ^13^C NMR (101 MHz, DMSO-*d*_*6*_) δ 166.77, 162.67, 162.15, 162.00 (d, *J*_C–F_ = 246.4 Hz), 159.74, 152.00, 147.53, 144.53, 142.69, 139.25, 136.54, 135.95, 133.56 (d, *J*_C–F_ = 10.4 Hz), 131.09 (d, *J*_C–F_ = 9.2 Hz), 130.63, 130.23, 129.51, 128.70 (d, *J*_C–F_ = 12.7 Hz), 128.28, 123.52, 122.21, 119.96, 116.10 (d, *J*_C–F_ = 21.1 Hz), 114.71 (d, *J*_C–F_ = 23.9 Hz), 113.23, 112.00, 110.73, 106.19, 100.01, 63.25, 44.95, 38.73, 36.90. LRMS (ESI) *m*/*z* 659.1 [M + H]^+^. HRMS (ESI) *m*/*z* for C_36_H_32_FN_8_O_4_ [M + H]^+^, calcd 659.2531, found 659.2532. UPLC purity 98.25% (*t*_R_ = 1.687 min).

#### *N*-{4-[(5-{3-[(Dimethylamino)­methyl]­phenyl}-6-(1-methyl-1*H*-pyrazol-4-yl)­furo­[2,3-*d*]­pyrimidin-4-yl)­oxy]­phenyl}-1-methyl-2-(4-methylphenyl)-3-oxo-2,3-dihydro-1*H*-pyrazole-4-carboxamide (**13**)

To a stirred solution of compound **23c** (120 mg, 0.20 mmol, 1.0 equiv) in tetrahydrofuran (2.0 mL) and *N*,*N*-dimethylformamide (2.0 mL) were added *N*,*N*-dimethyl­[3-(4,4,5,5-tetramethyl-1,3,2-dioxaborolan-2-yl)­phenyl]­methanamine hydrochloride (89 mg, 0.30 mmol, 1.5 equiv), Pd­(dppf)­Cl_2_ (44 mg, 0.06 mmol, 30 mol %), and aqueous Na_2_CO_3_ (2M, 127 mg, 1.20 mmol, 6.0 equiv). The resulting mixture was degassed for 30 min and backfilled with Argon_(g)_. After stirring for 16 h at 80 °C, the reaction mixture was allowed to cool to room temperature. It was then filtered through a pad of Celite, diluted with 10 mL of water, washed with 10 mL of saturated aqueous NaHCO_3_, and extracted with three 10 mL portions of dichloromethane. The combined organic extracts were washed with brine, dried over anhydrous MgSO_4_, filtered, and concentrated *in vacuo*. The crude residue was purified by automated flash column chromatography (Combiflash, 0–20% methanol in dichloromethane) followed by preparative thin layer chromatography (10% methanol in dichloromethane) to yield the title compound **13** (53 mg, 0.08 mmol, 41%) as a yellow solid. ^1^H NMR (400 MHz, DMSO-*d*_*6*_) δ 10.38 (s, 1H), 8.56 (s, 1H), 8.47 (s, 1H), 8.03 (s, 1H), 7.65–7.57 (m, 3H), 7.56 (ddd, *J* = 7.6, 1.6, 1.2 Hz, 1H), 7.51 (d, *J* = 0.4 Hz, 1H), 7.46 (d, *J* = 7.6, 7.6 Hz, 1H), 7.41–7.32 (m, 5H), 7.14 (d, *J* = 8.8 Hz, 2H), 3.85 (s, 3H), 3.45 (s, 2H), 3.39 (s, 3H), 2.39 (s, 3H), 2.15 (s, 6H). ^13^C NMR (101 MHz, DMSO-*d*_*6*_) δ 166.73, 162.62, 162.02, 159.94, 151.94, 147.42, 144.52, 141.01, 139.16, 138.62, 136.51, 136.03, 130.72, 130.27, 129.91, 129.52, 129.46, 128.85, 128.76, 128.27, 127.72, 122.10, 119.86, 111.93, 110.68, 106.16, 99.74, 62.94, 44.66, 38.68, 36.49, 20.69. LRMS (ESI) *m*/*z* 655.2 [M + H]^+^. HRMS (ESI) *m*/*z* for C_37_H_35_N_8_O_4_ [M + H]^+^, calcd 655.2781, found 655.2788. UPLC purity 94.31% (*t*_R_ = 1.764 min).

#### *N*-{4-[(5-{3-[(Dimethylamino)­methyl]­phenyl}-6-(1-methyl-1*H*-pyrazol-4-yl)­furo­[5,4-*d*]­pyrimidin-4-yl)­oxy]­phenyl}-1-methyl-3-oxo-2-[4-(trifluoromethyl)­phenyl]-2,3-dihydro-1*H*-pyrazole-4-carboxamide (**14**)

To a stirred solution of compound **23d** (23 mg, 0.04 mmol, 1.0 equiv) in *N*,*N*-dimethylformamide (1.0 mL) were added *N*,*N*-dimethyl­[3-(4,4,5,5-tetramethyl-1,3,2-dioxaborolan-2-yl)­phenyl]­methanamine hydrochloride (11 mg, 0.04 mmol, 1.2 equiv), Pd­(dppf)­Cl_2_ (3 mg, 0.004 mmol, 12 mol %), and aqueous Na_2_CO_3_ (2M, 11 mg, 0.10 mmol, 3.0 equiv). The resulting mixture was degassed for 30 min and backfilled with Argon_(g)_. After stirring for 3 h at 80 °C, the reaction mixture was allowed to cool to room temperature. It was then filtered through a pad of Celite, diluted with 10 mL of water, washed with 10 mL of saturated aqueous NaHCO_3_, and extracted with three 10 mL portions of dichloromethane. The combined organic extracts were washed with brine, dried over anhydrous MgSO_4_, filtered, and concentrated *in vacuo*. The crude residue was purified by preparative thin layer chromatography (8% methanol in dichloromethane) to yield the title compound **14** (16 mg, 0.02 mmol, 64%) as a brown solid. ^1^H NMR (600 MHz, DMSO-*d*_*6*_) δ 10.21 (s, 1H), 8.70 (s, 1H), 8.47 (s, 1H), 8.04 (s, 1H), 7.97 (d, *J* = 8.4 Hz, 2H), 7.73 (d, *J* = 8.4 Hz, 2H), 7.63 (d, *J* = 9.0 Hz, 2H), 7.60 (s, 1H), 7.56 (d, *J* = 7.2 Hz, 1H), 7.51 (s, 1H), 7.46 (dd, *J* = 7.8, 7.2 Hz, 1H), 7.36 (d, *J* = 7.8 Hz, 1H), 7.16 (d, *J* = 9.0 Hz, 2H), 3.85 (s, 3H), 3.53 (s, 3H), 3.49 (s, 2H), 2.15 (s, 6H). ^13^C NMR (151 MHz, DMSO-*d*_*6*_) δ 166.77, 162.67, 162.30, 159.58, 152.01, 147.57, 144.56, 144.09, 136.55, 135.88, 135.83, 130.81, 130.31, 129.53, 128.70 (q, *J*_C–F_ = 32.31 Hz), 128.36, 127.29, 126.52 (q, *J*_C–F_ = 4.38 Hz), 123.84 (q, *J*_C–F_ = 272.40 Hz), 122.22, 120.04, 111.93, 110.68, 106.18, 100.42, 73.48, 69.76, 40.04, 38.73, 37.21, 24.93. LRMS (ESI) *m*/*z* 709.2 [M + H]^+^. HRMS (ESI) *m*/*z* for C_37_H_32_F_3_N_8_O_4_ [M + H]^+^, calcd 709.2499, found 709.2480. UPLC purity 94.45% (*t*_R_ = 2.013 min).

#### *N*-[4-({5-[3-(Acetylamino)­phenyl]-6-(1-methyl-1*H*-pyrazol-4-yl)­furo­[2,3-*d*]­pyrimidin-4-yl}­oxy)­phenyl]-2-(4-fluorophenyl)-1-methyl-3-oxo-2,3-dihydro-1*H*-pyrazole-4-carboxamide (**16**)

To a stirred solution of compound **1** (15 mg, 0.02 mmol, 1.0 equiv) in dichloromethane (1.0 mL) were added triethylamine (7 μL, 0.05 mmol, 2.1 equiv) and acetyl chloride (3 μL, 0.04 mmol, 1.7 equiv). The resulting mixture was stirred at room temperature. After stirring for 16 h, the resulting precipitate was collected by filtration, washed with 10 mL of cold dichloromethane, and dried *in vacuo* to yield the title compound **16** (4 mg, 0.01 mmol, 25%) as a yellow solid. ^1^H NMR (600 MHz, DMSO-*d*_*6*_) δ 10.34 (s, 1H), 10.06 (s, 1H), 8.59 (s, 1H), 8.47 (s, 1H), 8.12 (s, 1H), 7.93 (s, 1H), 7.63 (d, *J* = 8.7 Hz, 2H), 7.59–7.54 (m, 4H), 7.47–7.39 (m, 3H), 7.33 (d, *J* = 7.8 Hz, 1H), 7.16 (d, *J* = 8.7 Hz, 2H), 3.86 (s, 3H), 3.47 (s, 3H), 2.05 (s, 3H). ^13^C NMR (151 MHz, DMSO-*d*_*6*_) δ 168.54, 166.72, 162.57, 162.14 (d, *J*_C–F_ = 247.2 Hz), 162.12, 161.33, 159.92, 152.00, 147.41, 144.61, 141.33, 139.28, 136.67, 136.07, 130.83, 130.45 (d, *J*_C–F_ = 9.1 Hz), 129.66, 128.82, 128.40, 124.98, 122.24, 120.67, 129.95, 118.87, 116.47 (d, *J*_C–F_ = 23.1 Hz), 111.83, 110.63, 106.11, 99.69, 38.76, 36.56, 23.99. LRMS (ESI) *m*/*z* 659.3 [M + H]^+^. HRMS (ESI) *m*/*z* for C_35_H_27_FN_8_NaO_5_ [M + Na]^+^, calcd 681.1986, found 681.1981. HPLC purity 94.08% (*t*_R_ = 18.27 min).

#### 2-(4-Fluorophenyl)-1-methyl-*N*-{4-[(5-{3-[(4-methylpiperazin-1-yl)­methyl]­phenyl}-6-(1-methyl-1*H*-pyrazol-4-yl)­furo­[5,4-*d*]­pyrimidin-4-yl)­oxy]­phenyl}-3-oxo-2,3-dihydro-1*H*-pyrazole-4-carboxamide (**17**)

To a stirred solution of compound **24a** (40 mg, 0.06 mmol, 1.0 equiv) in dichloromethane (2.0 mL) at 0 °C were added 1-methylpiperazine (10 mg, 0.10 mmol, 1.6 equiv) and sodium triacetoxyborohydride (27 mg, 0.13 mmol, 2.0 equiv). After stirring for 16 h at room temperature, the reaction mixture was filtered through a pad of Celite, diluted with 10 mL of water, washed with 10 mL of saturated aqueous NaHCO_3_, and extracted into three 10 mL portions of dichloromethane. The combined organic layers were washed with brine, dried over anhydrous MgSO_4_, filtered, and concentrated *in vacuo*. The crude residue was purified by preparative thin layer chromatography (8% methanol in dichloromethane, containing 1% NH_4_OH) to yield the title compound **17** (34 mg, 0.05 mmol, 75%) as a yellow solid. ^1^H NMR (600 MHz, DMSO-*d*_*6*_) δ 10.33 (s, 1H), 8.59 (s, 1H), 8.46 (s, 1H), 8.02 (s, 1H), 7.62 (d, *J* = 9.0 Hz, 2H), 7.58–7.54 (m, 3H), 7.53 (ddd, *J* = 7.2, 1.8, 1.8 Hz, 1H), 7.50 (s, 1H), 7.47–7.41 (m, 3H), 7.33 (ddd, *J* = 7.8, 1.8, 1.2 Hz, 1H), 7.14 (d, *J* = 9.0 Hz, 2H), 3.85 (s, 3H), 3.48–3.44 (m, 5H), 2.40–2.10 (m, 8H), 2.06 (s, 3H). ^13^C NMR (151 MHz, DMSO-*d*_*6*_) δ 166.76, 162.69, 162.14 (d, *J*_C–F_ = 247.5 Hz), 162.12, 159.90, 151.99, 144.49, 144.54, 141.34, 138.46, 136.57, 136.06, 130.84, 130.45 (d, *J*_C–F_ = 9.2 Hz), 130.21, 129.54, 129.03, 128.67, 128.39, 128.26, 122.23, 119.97, 116.48 (d, *J*_C–F_ = 22.8 Hz), 112.01, 110.71, 106.16, 99.67, 61.96, 54.47, 52.45, 45.59, 38.76, 36.56. LRMS (ESI) *m*/*z* 714.3 [M + H]^+^. HRMS (ESI) *m*/*z* for C_39_H_36_FN_9_O_4_ [M + H]^+^, calcd 714.2953, found 714.2951. HPLC purity 97.01% (*t*_R_ = 15.08 min).

#### *N*-{4-[(5-{4-[(Dimethylamino)­methyl]­phenyl}-6-(1-methyl-1*H*-pyrazol-4-yl)­furo­[2,3-*d*]­pyrimidin-4-yl)­oxy]­phenyl}-2-(4-fluorophenyl)-1-methyl-3-oxo-2,3-dihydro-1*H*-pyrazole-4-carboxamide (**19**)

To a stirred solution of compound **23a** (58 mg, 0.10 mmol, 1.0 equiv) in tetrahydrofuran (1.0 mL), *N*,*N*-dimethylformamide (1.0 mL) and ethanol (0.5 mL) were added *N*,*N*-dimethyl-1-[4-(4,4,5,5-tetramethyl-1,3,2-dioxaborolan-2-yl)­phenyl]­methanamine hydrochloride (34 mg, 0.11 mmol, 1.2 equiv), Pd­(dppf)­Cl_2_ (7 mg, 0.01 mmol, 10 mol %), and aqueous Na_2_CO_3_ (2M, 43 mg, 0.41 mmol, 4.2 equiv). The resulting mixture was degassed for 30 min and backfilled with Argon_(g)_. After stirring for 3 h at 80 °C, the reaction mixture was allowed to cool to room temperature. It was then filtered through a pad of Celite, diluted with 10 mL of water, washed with 10 mL of saturated aqueous NaHCO_3_, and extracted with three 10 mL portions of dichloromethane. The combined organic extracts were washed with brine, dried over anhydrous MgSO_4_, filtered, and concentrated *in vacuo*. The crude residue was purified by preparative thin layer chromatography (10% methanol in dichloromethane) to yield the title compound **19** (52 mg, 0.08 mmol, 82%) as a yellow solid. ^1^H NMR (400 MHz, DMSO-*d*_*6*_) δ 10.34 (s, 1H), 8.59 (s, 1H), 8.46 (s, 1H), 8.05 (s, 1H), 7.62 (d, *J* = 9.0 Hz, 2H), 7.59 (d, *J* = 7.8 Hz, 2H), 7.56 (dd, *J* = 9.0, 5.2 Hz, 2H), 7.443 (s, 1H), 7.438 (dd, *J* = 9.0, 8.4 Hz, 2H), 7.40 (d, *J* = 7.8 Hz, 2H), 7.15 (d, *J* = 9.0 Hz, 2H), 3.86 (s, 3H), 3.46 (s, 3H), 3.45 (s, 2H), 2.17 (s, 6H). ^13^C NMR (101 MHz, DMSO-*d*_*6*_) δ 166.75, 162.64, 162.15 (d, *J*_C–F_ = 248.3 Hz), 162.12, 159.92, 151.96, 147.47, 144.53, 141.34, 138.93, 136.47, 136.07, 130.45 (d, *J*_C–F_ = 9.3 Hz), 129.93, 129.53, 128.97, 128.74, 128.39 (d, *J*_C–F_ = 2.9 Hz), 122.19, 120.00, 116.48 (d, *J*_C–F_ = 23.1 Hz), 111.89, 110.76, 106.20, 99.69, 63.04, 44.97, 38.76, 36.56. LRMS (ESI) *m*/*z* 659.2 [M + H]^+^. HRMS (ESI) *m*/*z* for C_36_H_32_FN_8_O_4_ [M + H]^+^, calcd 659.2531, found 659.2533. UPLC purity 96.84% (*t*_R_ = 1.669 min).

#### 1-(4-Fluorophenyl)-*N*-{4-[(5-{4-[(4-methylpiperazin-1-yl)­methyl]­phenyl}-6-(1-methyl-1*H*-pyrazol-4-yl)­furo­[2,3-*d*]­pyrimidin-4-yl)­oxy]­phenyl}-2-oxo-1,2-dihydropyridine-3-carboxamide (**20**)

To a stirred solution of compound **26c** (87 mg, 0.14 mmol, 1.0 equiv) in dichloromethane (3.5 mL) at 0 °C were added 1-methylpiperazine (21 mg, 0.21 mmol, 1.5 equiv) and sodium triacetoxyborohydride (73 mg, 0.34 mmol, 2.5 equiv. After stirring for 16 h at room temperature, the reaction mixture was filtered through a pad of Celite, diluted with 10 mL of water, washed with 10 mL of saturated aqueous NaHCO_3_, and extracted with three 10 mL portions of dichloromethane. The combined organic extracts were washed with brine, dried over anhydrous MgSO_4_, filtered, and concentrated *in vacuo*. The crude residue was purified by automated flash column chromatography (0–10% methanol in dichloromethane, containing 10% NH_4_OH) to yield the title compound **20** (47 mg, 0.07 mmol, 48%) as a yellow solid. ^1^H NMR (600 MHz, DMSO-*d*_*6*_) δ 11.95 (s, 1H), 8.57 (dd, *J* = 7.2, 2.4 Hz, 1H), 8.47 (s, 1H), 8.10 (dd, *J* = 6.6, 2.4 Hz, 1H), 8.06 (s, 1H), 7.71 (d, *J* = 9.0 Hz, 2H), 7.63–7.57 (m, 4H), 7.46 (s, 1H), 7.44–7.38 (m, 4H), 7.18 (d, *J* = 9.0 Hz, 2H), 6.71 (dd, *J* = 7.2, 6.6 Hz, 1H), 3.86 (s, 3H), 3.51 (s, 2H), 2.47–2.19 (s, 8H), 2.14 (s, 3H). ^13^C NMR (151 MHz, DMSO-*d*_*6*_) δ 166.77, 162.57, 161.86 (d, *J*_C–F_ = 244.5 Hz), 161.82, 161.16, 151.98, 148.03, 144.74, 144.56, 143.89, 138.46, 136.52, 136.29 (d, *J*_C–F_ = 3.2 Hz), 135.68, 129.94, 129.59, 129.31 (d, *J*_C–F_ = 8.9 Hz), 128.89, 128.68, 122.19, 120.77, 120.42, 116.05 (d, *J*_C–F_ = 23.0 Hz), 111.92, 110.73, 106.94, 106.27, 61.68, 54.69, 52.57, 45.69, 38.77. LRMS (ESI) *m*/*z* 711.2 [M + H]^+^. HRMS (ESI) *m*/*z* for C_40_H_36_F_1_N_8_O_4_, calcd 711.2844 [M + H]^+^, found 711.2854. UPLC purity 97.44% (*t*_R_ = 2.030 min).

#### 2-(4-Fluorophenyl)-1-methyl-*N*-{4-[(5-{4-[(4-methylpiperazin-1-yl)­methyl]­phenyl}-6-(1-methyl-1*H*-pyrazol-4-yl)­furo­[2,3-*d*]­pyrimidin-4-yl)­oxy]­phenyl}-3-oxo-2,3-dihydro-1*H*-pyrazole-4-carboxamide (**21**)

To a stirred solution of compound **24b** (83 mg, 0.13 mmol, 1.0 equiv) in dichloromethane (3.5 mL) at 0 °C were added 1-methylpiperazine (20 mg, 0.20 mmol, 1.5 equiv) and sodium triacetoxyborohydride (70 mg, 0.33 mmol, 2.5 equiv). After stirring for 16 h at room temperature, the reaction mixture was filtered through a pad of Celite, diluted with 10 mL of water, washed with 10 mL of saturated aqueous NaHCO_3_, and extracted with three 10 mL portions of dichloromethane. The combined organic extracts were washed with brine, dried over anhydrous MgSO_4_, filtered, and concentrated *in vacuo*. The crude residue was purified by automated flash column chromatography (0–10% methanol in dichloromethane, containing 1% NH_4_OH) to yield the title compound **21** (83 mg, 0.13 mmol, 1.0 equiv) as a yellow solid. ^1^H NMR (600 MHz, DMSO-*d*_*6*_) δ 10.33 (s, 1H), 8.59 (s, 1H), 8.46 (s, 1H), 8.05 (s, 1H), 7.62 (d, *J* = 9.0 Hz, 2H), 7.59 (d, *J* = 8.1 Hz, 2H), 7.56 (dd, *J* = 9.0, 4.8 Hz, 2H), 7.45 (s, 1H), 7.44 (dd, *J* = 9.0, 8.4 Hz, 2H), 7.40 (d, *J* = 8.1 Hz, 2H), 7.15 (d, *J* = 9.0 Hz, 2H), 3.85 (s, 3H), 3.51 (s, 2H), 3.46 (s, 3H), 2.46–2.24 (m, 8H), 2.14 (s, 3H). ^13^C NMR (101 MHz, DMSO-*d*_*6*_) δ 166.77, 162.65, 162.17 (d, *J*_C–F_ = 247.5 Hz), 162.13, 161.35, 159.94, 151.98, 147.51, 144.55, 141.34, 138.44, 136.52, 136.06, 130.47 (d, *J*_C–F_ = 9.4 Hz), 129.96, 129.58, 128.92, 128.70, 128.40, 122.18, 120.02, 116.50 (d, *J*_C–F_ = 22.8 Hz), 111.93, 110.76, 106.24, 99.70, 61.69, 54.67, 52.54, 45.65, 38.78, 36.58. LRMS (ESI) *m*/*z* 714.3 [M + H]^+^. HRMS (ESI) *m*/*z* for C_39_H_37_FN_9_O_4_ [M + H]^+^, calcd 714.2953, found 714.2953. UPLC purity 95.40% (*t*_R_ = 1.694 min).

### X-ray Crystallography

#### Protein Expression and Purification

The MerTK kinase domain (residues 571–864) and the catalytic domain of PTP1B (residues 1–283) were synthesized as a bicistronic construct and cloned into the pET28a vector, with an N-terminal His6 tag and a thrombin cleavage site upstream of MerTK. The intergenic region included a ribosomal binding site. The plasmid was transformed into Rosetta­(DE3)­pLysS competent cells, which were cultured in TB medium at 37 °C in a 5L Sartorius bioreactor. Protein expression was induced at 15 °C with 0.2 mM IPTG, and cell pellets were harvested after 6 h of induction by centrifugation, then stored at −80 °C.

Approximately 10 g of cell pellets were thawed and resuspended in 30 mL of lysis buffer (50 mM Tris-HCl, pH 7.5, 500 mM NaCl, 1x protease inhibitor cocktail (Roche)). Cells were lysed on ice by sonication for a total of 20 min, with 2-s pulses followed by 10 s of rest. The lysate was clarified by centrifugation and loaded onto a HisPrep FF 16/10 column (Cytiva) using an AKTA FPLC (Cytiva). The protein was eluted with a linear gradient to 100% elution buffer (50 mM Tris-HCl, pH 7.5, 500 mM NaCl, 500 mM imidazole) over 20 column volumes. Peak fractions containing MerTK were analyzed by SDS-PAGE, pooled, and concentrated using Amicon Ultra-15 concentrators (10,000 molecular weight cutoff, Millipore).

The concentrated MerTK protein was loaded onto a HiPrep 26/10 Desalting column (Cytiva) and buffer-exchanged into the final buffer (20 mM Tris-HCl, pH 8.0, 500 mM NaCl, 2 mM TCEP) using an AKTA FPLC system. Peak fractions were pooled and further concentrated using Amicon Ultra-15 concentrators to a final concentration of 10–15 mg/mL. For additional purification, the MerTK protein was subjected to size exclusion chromatography on a HiLoad Superdex 200 pg column (16/300, Cytiva). The purity of the protein was assessed by SDS-PAGE, and the purified MerTK was stored at −80 °C for future studies.

#### Crystallization

Crystals of MerTK in complex with **1** and **11** were obtained by vapor diffusion using the hanging drop method at 14 °C. The protein (9.5 mg/mL in crystallization buffer: 20 mM Tris-HCl, pH 8.0, 500 mM NaCl, 2 mM TCEP) was incubated with the inhibitor (dissolved in DMSO) at a final concentration of 8 mM, and the mixture was gently rocked overnight. The protein-inhibitor solution (0.5 μL) was mixed 1:1 with the crystallization solution (0.5 μL), which contained 36% PEG 200, 50 mM calcium chloride dihydrate, and 100 mM MES monohydrate, pH 7.1, and equilibrated. Plate-like crystals appeared after approximately 10 days. Crystals of MerTK in complex with **6** were similarly obtained by vapor diffusion at 14 °C. The size-exclusion chromatography-purified protein (15 mg/mL) in crystallization buffer was incubated with the inhibitor at a final concentration of 2 mM and gently rocked overnight. The protein-inhibitor solution (1 μL) was mixed 1:1 with the crystallization solution (1 μL), which contained 24% PEG 200, 50 mM calcium chloride dihydrate, and 100 mM MES monohydrate, pH 6.4. Prior to diffraction data collection, crystals were mounted on Hampton loops and frozen in liquid nitrogen.

#### Structure Determination

Data were collected at the National Synchrotron Radiation Research Center (NSRRC) TPS05A beamline and processed using the HKL2000 ^35^ software. For **6**, seven individual data sets were processed and merged using XDS^36^ and KAMO.^37^ The structures of the MerTK/inhibitor complexes were determined by molecular replacement with Phaser,^38^ using an inhibitor-bound MerTK structure as the starting model. Refinement was performed using Phenix,^39^ with manual adjustments to the model made in Coot.

#### MerTK and Tyro3 Enzyme Inhibition Assay

Purified MerTK or Tyro3 kinases were mixed with either a test compound or the vehicle control (DMSO). For MerTK assay, the buffer consisted of 10 mM MgCl_2_, 4 mM MnCl_2_, 25 mM Tris (pH 7.4), 2 mM DTT and 0.5 mM Na_3_VO_4_. It also contained protein stabilizers including 0.01% BSA, 0.01% Brij 35 and 0.02% Triton X-100. For Tyro3, the buffer included 20 mM MgCl_2_,40 mM Tris (pH 7.4), 2 mM DTT, 0.5 mM Na_3_VO_4_ and 0.01% BSA. The reactions were initiated by adding the substrate and ATP (12 μM ATP for MerTK and 50 μM ATP for Tyro3) to the mixtures, followed by incubation at 30 °C for 3 h. Luminescence-based assays (Kinase-Glo assay for MerTK and ADP-Glo assay for Tyro3) were used to measure kinase activity.

#### Thermal Shift Assay

The thermal shift assay was conducted to evaluate the binding of compounds **1** and **11** to MerTK_571–864_ and mTyro3_485–800_, respectively. Murine Tyro3_485–800_ (mTyro3) with high sequence identity of 98.0% with human Tyro3_495–810_ (hTyro3) was used for the assay due to the difficulty in the expression and purification of hTyro3. The assay solution contained either 2 μM MerTK or 2.1 μM mTyro3 protein, prepared in respective buffer: MerTK buffer (25 mM Tris-HCl, pH 7.5, 0.5 M NaCl, 2 mM DTT) or mTyro3 buffer (25 mM Tris-HCl, pH 8.0, 0.5 M NaCl, 2 mM TCEP). Each reaction had a final volume of 20 μL and included 8× concentrated Protein Thermal Shift dye.

The assays were performed in triplicate using a Real-Time PCR System. Protein denaturation curves were analyzed using the Boltzmann sigmoid equation in Protein Thermal Shift Software v1.4 to determine the melting temperature (Tm). Each experiment was independently repeated at least twice. The thermal shift (ΔTm) was calculated as the difference between the Tm of the protein in DMSO and the Tm in the presence of each compound.

#### Docking Study

The three-dimensional structure of human Tyro3 was not available; however, the murine Tyro3 structure is accessible in the RCSB PDB database. Therefore, the X-ray crystallographic structure of murine Tyro3 (PDB entry: 3QUP) was used as a template for constructing the human Tyro3 model. To prepare the human Tyro3 protein model, the kinase domain sequence of murine Tyro3 (UniProtKB ID: P55144; amino acids 499–782) was aligned with the human Tyro3 sequence (UniProtKB ID: Q06418; amino acids 509–792) using BLAST,^40^ which revealed 99% identity with four differing residues. These four residues—V623, H702, E750, H782 in the murine structure—were mutated to their corresponding human residues (I633, Q712, D760, Q792) using the COOT software.^41^ The human Tyro3 protein model was then subjected to energy minimization using the CHARMm-based method in BIOVIA Discovery Studio (version 2021) to optimize the structure for docking and to allow movement in the protein structure, including the αC helix and the ATP-binding site. Subsequently, the structures of compounds **1** and **11** were docked into the human Tyro3 model using the flexible docking mode in GOLD (version 2022.3.0, CCDC, Cambridge, UK).^42^

#### In Vivo Pharmacokinetics Study

The animal studies were conducted following the protocols evaluated and approved by the Institutional Animal Care and Use Committee of the National Health Research Institutes (NHRI-IACUC-112084-A). Male ICR mice (weighing approximately 25–28 g) were provided by BioLASCO (Taiwan Co., Ltd.). A single 2 mg/kg (IV: intravenous) and 10 mg/kg (oral) dose of **11**, formulated as a DMSO/Cremophor/95% Glucose water, 10/20/70, v/v/v) for IV and 1% CMC (carboxymethyl cellulose)/Tween 80 (99.5%/0.5%, v/v) for oral administration, was administered to mice. Blood samples were collected from groups of three mice at time points 0 (before dosing), 0.03, 0.08, 0.25 (IV only), and 0.5, 1, 2, 4, 6, 8, 16, and 24 h postdosing by cardiac puncture. All samples were subsequently analyzed by LC-MS/MS. A noncompartmental method was used to analyze plasma concentration data.

#### Animal Studies

Six-to-seven-week-old female C57BL/6 mice were used for the implantation of MC38 murine colon cancer cells. Tumor cells were tested free of Mycoplasma spp. prior to injection into the animals. Mice were subcutaneously injected with 10^5^ cells in 100 μL of culture medium into the left flank using a 25–5/8 gauge needle. Tumor size and animal body weight were measured twice a week following tumor cell inoculation. Animals were randomized when the average tumor volume reached to approximately 50–60 mm^3^, and animals received either vehicle control or 50 mg/kg of **11** twice a day (BID), 5 days a week for 3 weeks. Dosing solution was freshly prepared daily in 10%DMA/40%PEG400/50% (1%CMC). Tumor volumes were calculated as L × W × W/2. Tumor growth inhibition (% TGI) was calculated as follows: % TGI = [1 – (ΔT/ΔC)] × 100, where ΔT represents the difference in average tumor volume between the measured day and day 0 for the treated groups, and ΔC represents the difference in average tumor volume between the measured day and day 0 for the control groups. At the end of 3-week treatment course, animals were euthanized and the whole blood was collected through cardiac puncture at the conclusion of the study. Blood samples were processed and were subjected to liver and kidney biomarkers analyses. The animal studies were conducted following the protocols evaluated and approved by the Institutional Animal Care and Use Committee of the National Health Research Institutes (NHRI-IACUC-110027-A).

#### Flow-Cytometry Analysis

MC38 tumor-bearing animals (average tumor volume around 150 mm^3^) were treated with 50 mg/kg of **11** twice a day, 5 days a week for 2 weeks. Tumor and spleen tissues from the control and treated animals were harvested 2 h after the last dose on day 11. Tissues were processed and digested with 0.1% collagenase III in DMEM medium to obtain single cell suspension. Thereafter, the cells were incubated with the mouse Fc receptor blocker for 10 min. The cells were then stained with an antibody cocktail containing antimouse antibody conjugate (from BioLegend) for Cd45, Cd3, Cd4, Cd8, Cd11b, Cd86, F4/80, and MerTK at 4 °C for 30 min. After staining, the cells were washed twice with a 1% BSA-containing buffer and analyzed by flow cytometry. For CD206 analysis, the cells were fixed and permeabilized using the Cyto X/Cytoperm kit (BD Biosciences) after surface marker staining, and then stained with antimouse CD206 antibody. Live and dead cells were distinguished using eFluo780 viability dye (BioLegend). Cells were analyzed using Attune NxT flow cytometer (ThermoFisher Scientific).

#### Statistical Analysis

Data are presented as mean ± standard error of the mean (SEM). A nonparametric *t* test was used to examine differences in mean values between groups. A *p* value of <0.05 was considered statistically significant. Statistical analysis was conducted using GraphPad Prism 9.

## Supplementary Material









## Notes

Crystallographic data; molecular formula strings­(CSV)
Coordinates of the docking models for Tyro3/1­(PDB)
Coordinates of the docking models for Tyro3/11­(PDB)
Experimental procedures and compound characterization data for **23a–32**; ^1^H and ^13^C NMR spectrum, HPLC trace and HRMS data of **1**, **2**, **3**, **4**, **5**, **7**, **8**, **9**, **11**, **12**, **13**, **14**, **16**, **17**, **19**, **20**, **21**(DOCX)

## Abbreviations

AML: acute myeloid leukemia
ALL: acute Lymphoblastic Leukemia
MerTK: Mer tyrosine kinase
NSCLC: nonsmall cell lung cancer
SI: selectivity index
TAM: Tyro3, Axl and MerTK
