# Supplementary material for: Structure-Based Design of Potent and Selective MerTK Inhibitors by Modulating the Conformation of αC Helix
Source: J Med Chem. 2025 May 20;68(11):10877–96. doi: 10.1021/acs.jmedchem.4c03092 (PMC12169616; doi:10.1021/acs.jmedchem.4c03092)
Supplement: Supplementary file 4 [file jm4c03092_si_004.docx]

**Supporting Information**

Structure-based Design of Potent and Selective MerTK Inhibitors by Modulating the Conformation of αC Helix

*Yi-Hui Peng, ^†^* *Mu-Chun Li,† Wan-Ching Yen,† Teng-Kuang Yeh,† Ching-Cheng Hsueh,† Fu-Ming Kuo,†You-Liang Lai,†Ling Chang,† Lung-Chun Lee,† Pei-Yi Chen,†Kuei-Jung Yen,† Teng-Yuan Chang,† Hsu-Yi Sun,†Chun-Yu Chang,† Su-Huei Hsieh,† Chen-Ming Yang,†Hsing-Pang Hsieh†* and Su-Ying Wu^†*^*

†Institute of Biotechnology and Pharmaceutical Research, National Health Research Institutes, 35, Keyan Road, Zhunan Town, Miaoli County 350, Taiwan, R.O.C.

Corresponding author:

*Su-Ying Wu,* Email:[suying@nhri.org.tw](mailto:suying@nhri.org.tw)

*Hsing-Pang Hsieh,* Email : [hphsieh@nhri.edu.tw](mailto:hphsieh@nhri.edu.tw)

**Table of Contents**

Experimental procedures and compound characterization data for 23a–32……… S3

[Figure S1. NMR spectra, HPLC trace, and HRMS data of compound 1…………...S](#S3)9

[Figure S2. NMR spectra, HPLC trace, and HRMS data of compound 2………….S](#S3)11

[Figure S3. NMR spectra, HPLC trace, and HRMS data of compound 3…..…..….S](#S3)13

[Figure S4. NMR spectra, HPLC trace, and HRMS data of compound 4…..…..….S](#S3)15

[Figure S5. NMR spectra, HPLC trace, and HRMS data of compound 5.…...…….S](#S3)17

[Figure S6. NMR spectra, HPLC trace, and HRMS data of compound 7.…..….….S](#S3)19

[Figure S7. NMR spectra, HPLC trace, and HRMS data of compound 8 ….……..S](#S3)21

[Figure S8. NMR spectra, HPLC trace, and HRMS data of compound 9……….....S](#S3)23

[Figure S9. NMR spectra, HPLC trace, and HRMS data of compound 11..…..…..S](#S3)25

[Figure S10. NMR spectra, HPLC trace, and HRMS data of compound 12……....S](#S3)27

[Figure S11. NMR spectra, HPLC trace, and HRMS data of compound 13……….S29](#_Supplementary_Figure_2.)

[Figure S12. NMR spectra, HPLC trace, and HRMS data of compound 14……….S](#S3)31

[Figure S13. NMR spectra, HPLC trace, and HRMS data of compound 16……….S](#S3)33

[Figure S14. NMR spectra, HPLC trace, and HRMS data of compound 17….…....S](#S3)35

[Figure S15. NMR spectra, HPLC trace, and HRMS data of compound 19……….S](#S3)37

[Figure S16. NMR spectra, HPLC trace, and HRMS data of compound 20.……....S](#S3)39

[Figure S17. NMR spectra, HPLC trace, and HRMS data of compound 21.………S](#S3)41

**Experimental procedures and compound characterization data for 23a–32**

***N*-(4-{[5-Bromo-6-(1-methyl-1*H*-pyrazol-4-yl)furo[2,3-*d*]pyrimidin-4-yl]oxy}phenyl)-2-(4-fluorophenyl)-1-methyl-3-oxo-2,3-dihydro-1*H*-pyrazole-4-carboxamide (23a)**

To a solution of aniline **22** (81 mg, 0.21 mmol, 1.0 equiv) in DMF (1.0 mL) at 0 °C were added carboxylic acid **27a** (54 mg, 0.23 mmol, 1.1 equiv), DIPEA (131 μL, 0.75 mmol, 3.6 equiv) and TBTU (87 mg, 0.27 mmol, 1.3 equiv), and then the reaction mixture was stirred at room temperature. After stirring for 16 h, the reaction mixture was concentrated *in vacuo*, and purified by Combiflash automated flash chromatography (1–3% methanol in dichloromethane) to yield the title compound **23a** (115 mg, 0.19 mmol, 91%) as a yellow solid. LRMS (ESI) *m*/*z* 604.1 [M + H]^+^.

***N*-(4-{[5-Bromo-6-(1-methyl-1*H*-pyrazol-4-yl)furo[2,3-*d*]pyrimidin-4-yl]oxy}phenyl)-2-(3-fluorophenyl)-1-methyl-3-oxo-2,3-dihydro-1*H*-pyrazole-4-carboxamide (23b)**

To a solution of aniline **22** (80 mg, 0.21 mmol, 1.0 equiv) in DMF (1.0 mL) at 0 °C were added carboxylic acid **27b** (54 mg, 0.23 mmol, 1.1 equiv), DIPEA (131 μL, 0.75 mmol, 3.6 equiv) and TBTU (87 mg, 0.27 mmol, 1.3 equiv), and then the reaction mixture was stirred at room temperature. After stirring for 16 h, the reaction mixture was concentrated *in vacuo*, and purified by Combiflash automated flash chromatography (1–3% methanol in dichloromethane) to yield the title compound **23b** (106 mg, 0.18 mmol, 85%) as a yellow solid. LRMS (ESI) *m*/*z* 604.1 [M + H]^+^.

***N*-(4-{[5-Bromo-6-(1-methyl-1*H*-pyrazol-4-yl)furo[2,3-*d*]pyrimidin-4-yl]oxy}phenyl)-1-methyl-2-(4-methylphenyl)-3-oxo-2,3-dihydro-1*H*-pyrazole-4-carboxamide (23c)**

To a solution of aniline **22** (302 mg, 0.78 mmol, 1.0 equiv) in DMF (4.0 mL) at 0 °C were added carboxylic acid **27c** (200 mg, 0.86 mmol, 1.1 equiv), DIPEA (490 μL, 2.81 mmol, 3.6 equiv) and TBTU (327 mg, 1.02 mmol, 1.3 equiv), and then the reaction mixture was stirred at room temperature. After stirring for 16 h, the reaction mixture was concentrated *in vacuo* and added water (50 mL). The resulting precipitate was collected and dried *in vacuo* to yield the title compound **23c** (433 mg, 0.72 mmol, 92%) as a yellow solid. LRMS (ESI) *m*/*z* 600.1 [M + H]^+^.

***N*-(4-{[5-Bromo-6-(1-methyl-1*H*-pyrazol-4-yl)furo[2,3-*d*]pyrimidin-4-yl]oxy}phenyl)-1-methyl-3-oxo-2-[4-(trifluoromethyl)phenyl]-2,3-dihydro-1*H*-pyrazole-4-carboxamide (23d)**

To a solution of aniline **22** (278 mg, 0.72 mmol, 1.0 equiv) in DMF (5.0 mL) at 0 °C were added carboxylic acid **27d** (206 mg, 0.72 mmol, 1.0 equiv), DIPEA (451 μL, 2.59 mmol, 3.6 equiv) and TBTU (300 mg, 0.94 mmol, 1.3 equiv), and then the reaction mixture was stirred at room temperature. After stirring for 16 h, the reaction mixture was concentrated *in vacuo*, and purified by Combiflash automated flash chromatography (0–5% methanol in dichloromethane) to yield the title compound **23d** (62 mg, 0.10 mmol, 13%) as a yellow solid. LRMS (ESI) *m*/*z* 654.0 [M + H]^+^.

***N*-(4-{[5-Bromo-6-(1-methyl-1*H*-pyrazol-4-yl)furo[2,3-*d*]pyrimidin-4-yl]oxy}phenyl)-2-(4-fluorophenyl)-1,5-dimethyl-3-oxo-2,3-dihydro-1*H*-pyrazole-4-carboxamide (23e)**

To a solution of aniline **22** (70 mg, 0.18 mmol, 1.0 equiv) in DMF (3.0 mL) at 0 °C were added carboxylic acid **27e** (50 mg, 0.20 mmol, 1.1 equiv), DIPEA (114 μL, 0.65 mmol, 3.6 equiv) and TBTU (76 mg, 0.24 mmol, 1.3 equiv), and then the reaction mixture was stirred at room temperature. After stirring for 12 h, the reaction mixture was concentrated *in vacuo* and added water (20 mL). The resulting precipitate was collected and dried *in vacuo* to yield the title compound **23e** (42 mg, 0.07 mmol, 37%) as a yellow solid. LRMS (ESI) *m*/*z*: 618.2 [M + H]^+^.

**2-(4-Fluorophenyl)-*N*-(4-{[5-(3-formylphenyl)-6-(1-methyl-1*H*-pyrazol-4-yl)furo[5,4-*d*]pyrimidin-4-yl]oxy}phenyl)-1-methyl-3-oxo-2,3-dihydro-1*H*-pyrazole-4-carboxamide (24a)**

To a solution of compound **23a** (100 mg, 0.17 mmol, 1.0 equiv) in tetrahydrofuran (2.0 mL) and *N*,*N*-dimethylformamide (2.0 mL) were added (3-formylphenyl)boronic acid (30 mg, 0.20 mmol, 1.2 equiv), Pd(dppf)Cl_2_ (12 mg, 0.02 mmol, 10 mol %), and 2.0 M Na_2_CO_3(aq)_ (53 mg, 0.50 mmol, 3.0 equiv). The reaction mixture was degassed for 30 min, refilled with Argon_(g)_, and stirred at 80 °C. After stirring for 3 h, the reaction mixture was cooled down to room temperature, filtered through Celite, added with water (10 mL), washed with sat. NaHCO_3(aq)_ (10 mL), and extracted into dichloromethane (10 mL × 3). The combined organic layers were washed with brine, dried over MgSO_4_, concentrated *in vacuo*, and purified by Combiflash automated flash chromatography (0–4% methanol in dichloromethane) to yield the title compound **24a** (98 mg, 0.16 mmol, 94%) as a beige solid. LRMS (ESI) *m*/*z* 630.1 [M + H]^+^.

**2-(4-Fluorophenyl)-*N*-(4-{[5-(4-formylphenyl)-6-(1-methyl-1*H*-pyrazol-4-yl)furo[2,3-*d*]pyrimidin-4-yl]oxy}phenyl)-1-methyl-3-oxo-2,3-dihydro-1*H*-pyrazole-4-carboxamide (24b)**

To a solution of compound **23a** (111 mg, 0.18 mmol, 1.0 equiv) in tetrahydrofuran (1.3 mL), *N*,*N*-dimethylformamide (1.3 mL) and ethanol (0.7 mL) were added (4-formylphenyl)boronic acid (41 mg, 0.27 mmol, 1.5 equiv), Pd(dppf)Cl_2_ (91 mg, 0.12 mmol, 68 mol %), and 2.0 M Na_2_CO_3(aq)_ (97 mg, 0.92 mmol, 5.0 equiv). The reaction mixture was degassed for 30 min, refilled with Argon_(g)_, and stirred at 80 °C. After stirring for 4 h, the reaction mixture was cooled down to room temperature, filtered through Celite, added with water (10 mL), washed with sat. NaHCO_3(aq)_ (10 mL), and extracted into dichloromethane (10 mL × 3). The combined organic layers were washed with brine, dried over MgSO_4_, concentrated *in vacuo*, and purified by Combiflash automated flash chromatography (0–7% methanol in dichloromethane) to yield the title compound **24b** (83 mg, 0.13 mmol, 72%) as a yellow solid. LRMS (ESI) *m*/*z* 630.1 [M + H]^+^.

***N*-(4-{[5-Bromo-6-(1-methyl-1*H*-pyrazol-4-yl)furo[2,3-*d*]pyrimidin-4-yl]oxy}phenyl)-1-(4-fluorophenyl)-6-methyl-2-oxo-1,2-dihydropyridine-3-carboxamide (25a)**

To a solution of aniline **22** (200 mg, 0.52 mmol, 1.0 equiv) in DMF (8.5 mL) at 0 °C were added carboxylic acid **28a** (154 mg, 0.62 mmol, 1.2 equiv), DIPEA (320 μL, 1.84 mmol, 3.5 equiv) and TBTU (249 mg, 0.78 mmol, 1.5 equiv), and then the reaction mixture was stirred at room temperature. After stirring for 10 h, the reaction mixture was concentrated *in vacuo* and added water (20 mL). The resulting precipitate was collected and dried *in vacuo* to yield the title compound **25a** (310 mg, 0.50 mmol, 97%) as a light-yellow solid. LRMS (ESI) *m*/*z*: 615.1 [M + H]^+^.

***N*-(4-{[5-Bromo-6-(1-methyl-1*H*-pyrazol-4-yl)furo[2,3-*d*]pyrimidin-4-yl]oxy}phenyl)-1-methyl-2-oxo-1,2-dihydropyridine-3-carboxamide (25b)**

To a solution of aniline **22** (150 mg, 0.39 mmol, 1.0 equiv) in DMF (4.5 mL) at 0 °C were added carboxylic acid **28b** (65 mg, 0.42 mmol, 1.1 equiv), DIPEA (244 μL, 1.40 mmol, 3.6 equiv) and TBTU (162 mg, 0.51 mmol, 1.3 equiv), and then the reaction mixture was stirred at room temperature. After stirring for 16 h, the reaction mixture was concentrated *in vacuo* and washed with cold ethyl acetate (20 mL). The resulting precipitate was collected and dried *in vacuo* to yield the title compound **25b** (137 mg, 0.26 mmol, 66%) as a yellow solid. LRMS (ESI) *m*/*z* 521.1 [M + H]^+^.

**1-(4-Fluorophenyl)-*N*-(4-{[5-(4-formylphenyl)-6-(1-methyl-1*H*-pyrazol-4-yl)furo[2,3-*d*]pyrimidin-4-yl]oxy}phenyl)-2-oxo-1,2-dihydropyridine-3-carboxamide (26c)**

To a solution of compound **25c** (132 mg, 0.22 mmol, 1.0 equiv) in tetrahydrofuran (1.6 mL), *N*,*N*-dimethylformamide (1.6 mL) and ethanol (0.8 mL) were added (4-formylphenyl)boronic acid (49 mg, 0.33 mmol, 1.5 equiv), Pd(dppf)Cl_2_ (48 mg, 0.07 mmol, 30 mol %), and 2.0 M Na_2_CO_3(aq)_ (116 mg, 1.09 mmol, 5.0 equiv). The reaction mixture was degassed for 30 min, refilled with Argon_(g)_, and stirred at 100 °C. After stirring for 2 h, the reaction mixture was cooled down to room temperature, filtered through Celite, added with water (10 mL), washed with sat. NaHCO_3(aq)_ (10 mL), and extracted into dichloromethane (10 mL × 3). The combined organic layers were washed with brine, dried over MgSO_4_, concentrated *in vacuo*, and purified by Combiflash automated flash chromatography (0–5% methanol in dichloromethane) to yield the title compound **26c** (87 mg, 0.14 mmol, 63%) as a yellow solid. LRMS (ESI) *m*/*z* 627.1 [M + H]^+^.

**4-Chloro-6-(1-methyl-1*H*-pyrazol-4-yl)-5-(3-nitrophenyl)furo[5,4-*d*]pyrimidine (30)**

To a solution of compound **29** (1.61 g, 4.55 mmol, 1.0 equiv) in 1,4-dioxane (46.0 mL) were added 1-methyl-4-(4,4,5,5-tetramethyl-1,3,2-dioxaborolan-2-yl)-1*H*-pyrazole (1.14 g, 5.48 mmol, 1.2 equiv), Pd(dppf)Cl_2_ (337 mg, 0.46 mmol, 10 mol %), and 2.0 M Na_2_CO_3(aq)_ (64 mg, 0.60 mmol, 6.0 equiv). The reaction mixture was degassed for 30 min, refilled with Argon_(g)_, and stirred at 80 °C. After stirred for 24 h, the reaction mixture was cooled down to room temperature, filtered through Celite, added with water (10 mL), washed with sat. NaHCO_3(aq)_ (10 mL), and extracted into dichloromethane (10 mL × 3). The combined organic layers were washed with brine, dried over MgSO_4_, concentrated *in vacuo*, and purified by flash chromatography (40% ethyl acetate in hexane) to yield the title compound **30** (1.17 g, 3.29 mmol, 72%) as a yellow solid. LRMS (ESI) *m*/*z* 356.1 [M + H]^+^.

**4-{[6-(1-Methyl-1*H*-pyrazol-4-yl)-5-(3-nitrophenyl)furo[2,3-*d*]pyrimidin-4-yl]oxy}aniline (31)**

To a solution of sodium hydride (200 mg, 5.00 mmol, 1.5 equiv) in *N*,*N*-dimethylformamide (66.0 mL) at 0 °C was added a solution of 4-aminophenol (540 mg, 4.95 mmol, 1.5 equiv) and **30** (1.17 g, 3.29 mmol, 1.0 equiv), and then the reaction mixture was stirred at room temperature. After stirring for 16 h, the reaction mixture was quenched with water (10 mL) and stirred for 30 min. Then the resulting precipitate was collected by filtration to yield the title compound **31** (1.40 g, 3.27 mmol, 99%) as a brown solid without further purification. LRMS (ESI) *m*/*z* 429.2 [M + H]^+^.

***N*^1^-(4-Fluorophenyl)-*N*^1^-(4-{[6-(1-methyl-1*H*-pyrazol-4-yl)-5-(3-nitrophenyl)furo[2,3-*d*]pyrimidin-4-yl]oxy}phenyl)cyclopropane-1,1-dicarboxamide (32)**

To a solution of **31** (200 mg, 0.47 mmol, 1.0 equiv) in dichloromethane (1.0 mL) were added carboxylic acid (115 mg, 0.52 mmol, 1.1 equiv) and EDCI (109 mg, 0.57 mmol, 1.2 equiv), and then the reaction mixture was stirred at room temperature. After stirring for 4.5 h, the reaction mixture was added sat. NaHCO_3(aq)_ (10 mL) and extracted into dichloromethane (10 mL × 3). The combined organic layers were washed with brine, dried over MgSO_4_, concentrated *in vacuo*, and purified by flash chromatography (2% methanol in dichloromethane) to yield the title compound **32** (216 mg, 0.34 mmol, 73%) as a yellow solid. LRMS (ESI) *m*/*z* 634.1 [M + H]^+^.


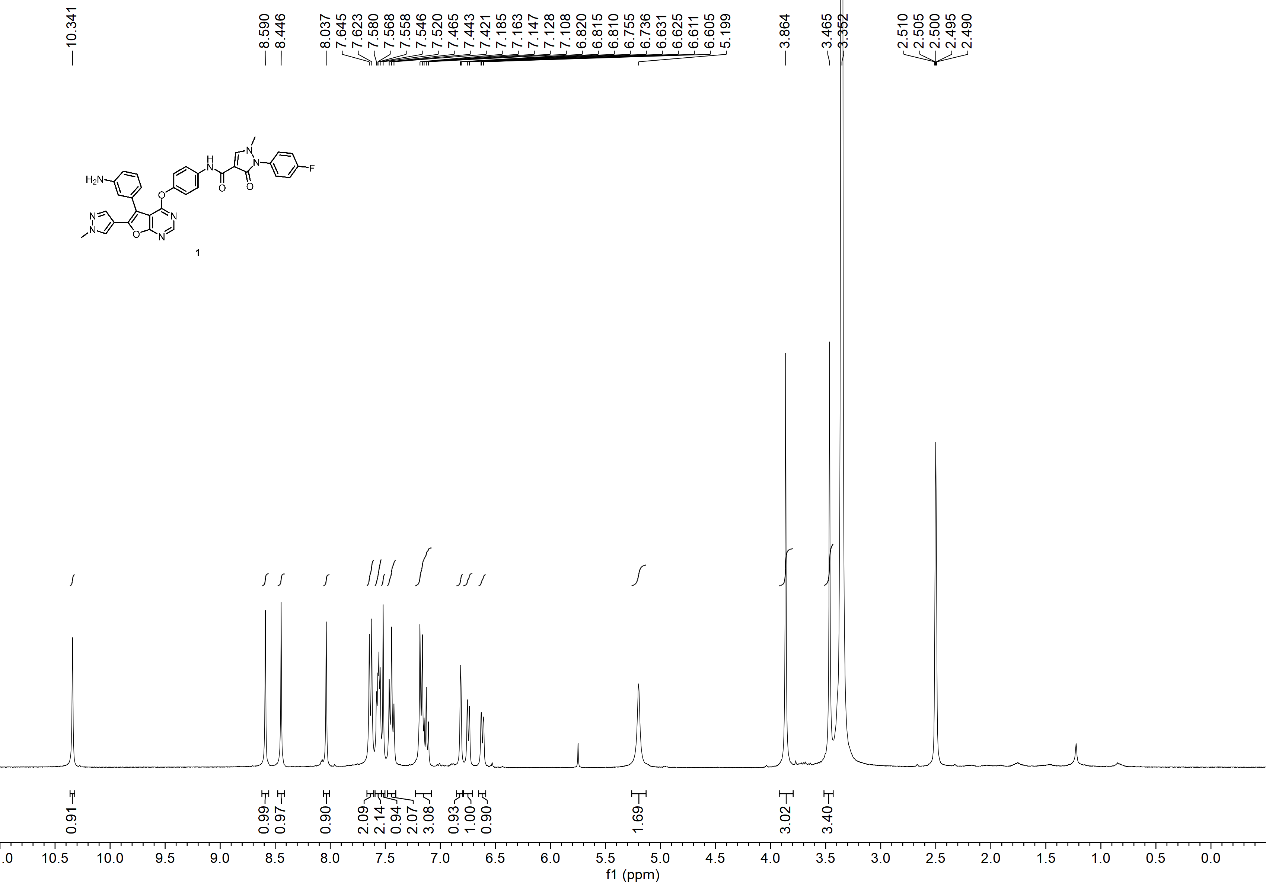


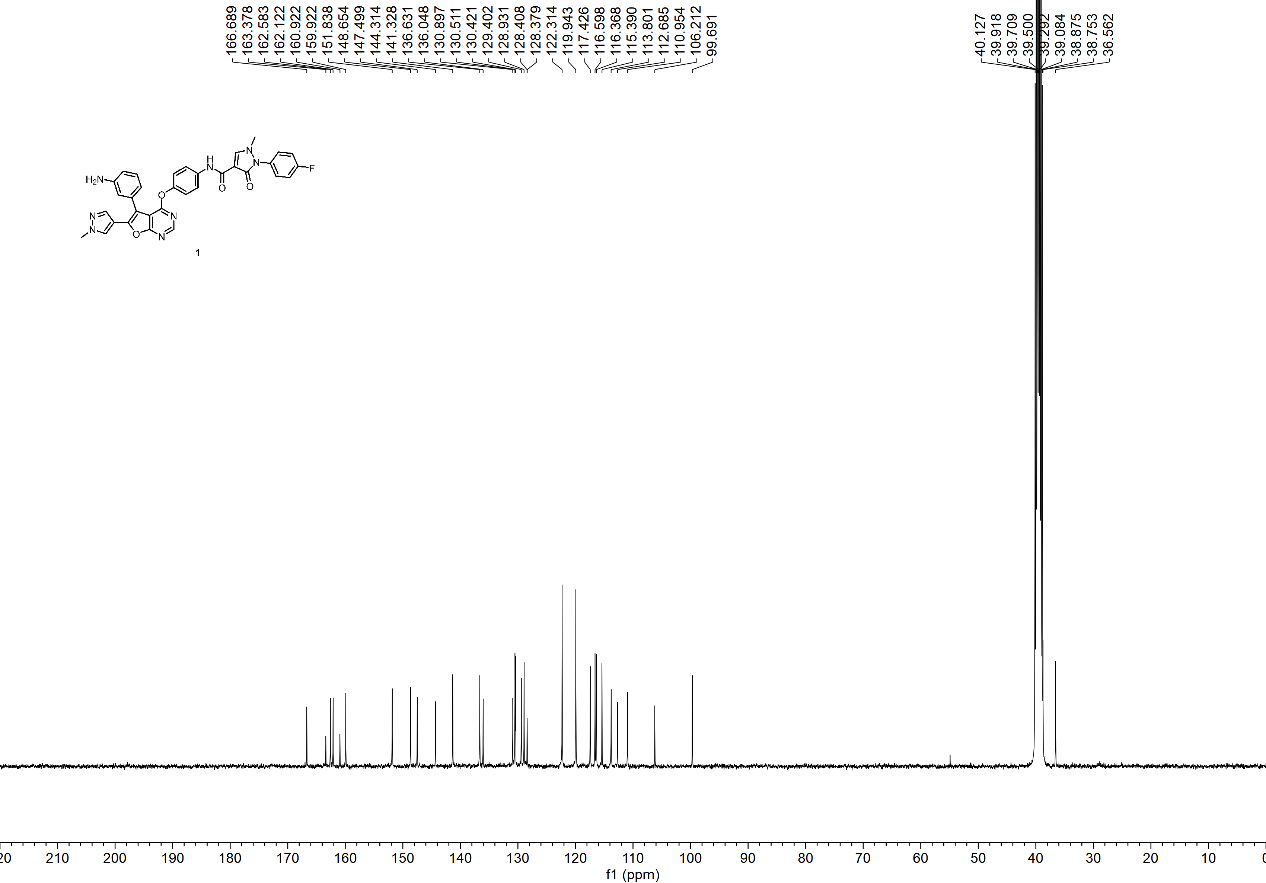


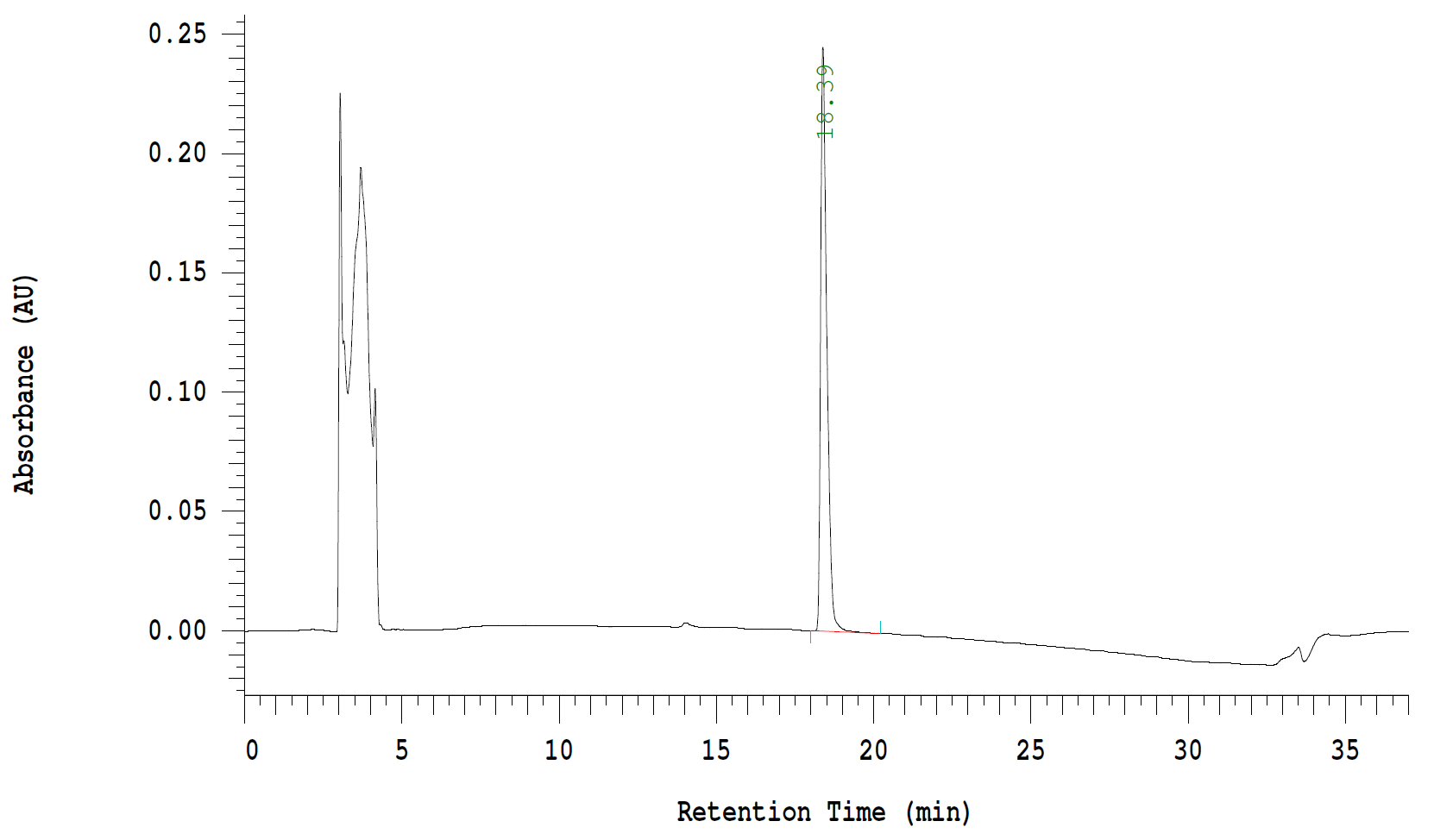


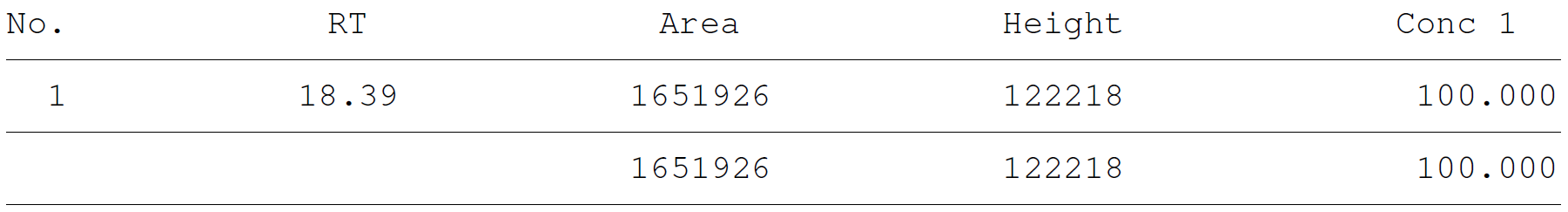


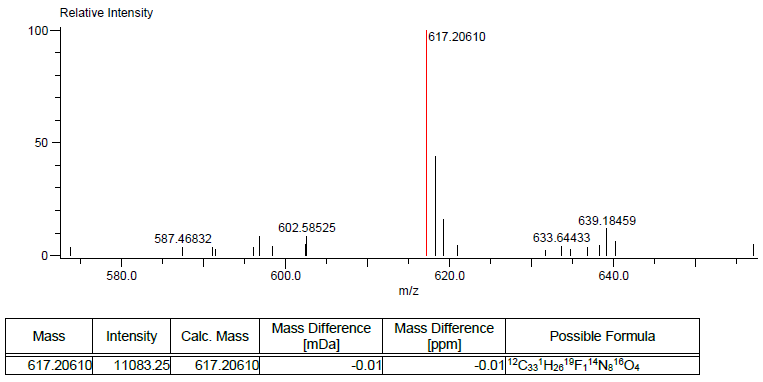


Figure S-1.^1^H and ^13^C NMR spectra in DMSO-*d*_6_, HPLC trace, and HRMS data of compound **1**_._


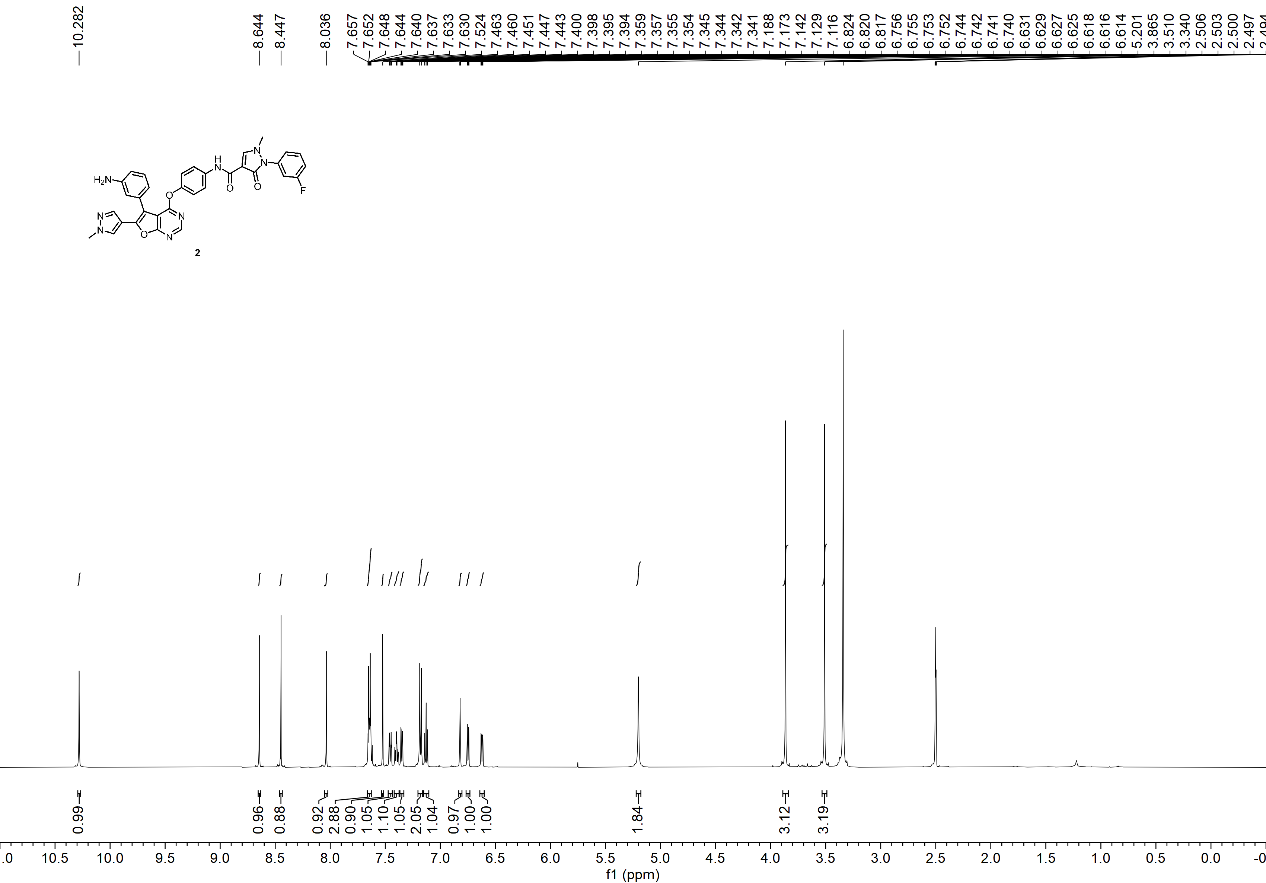


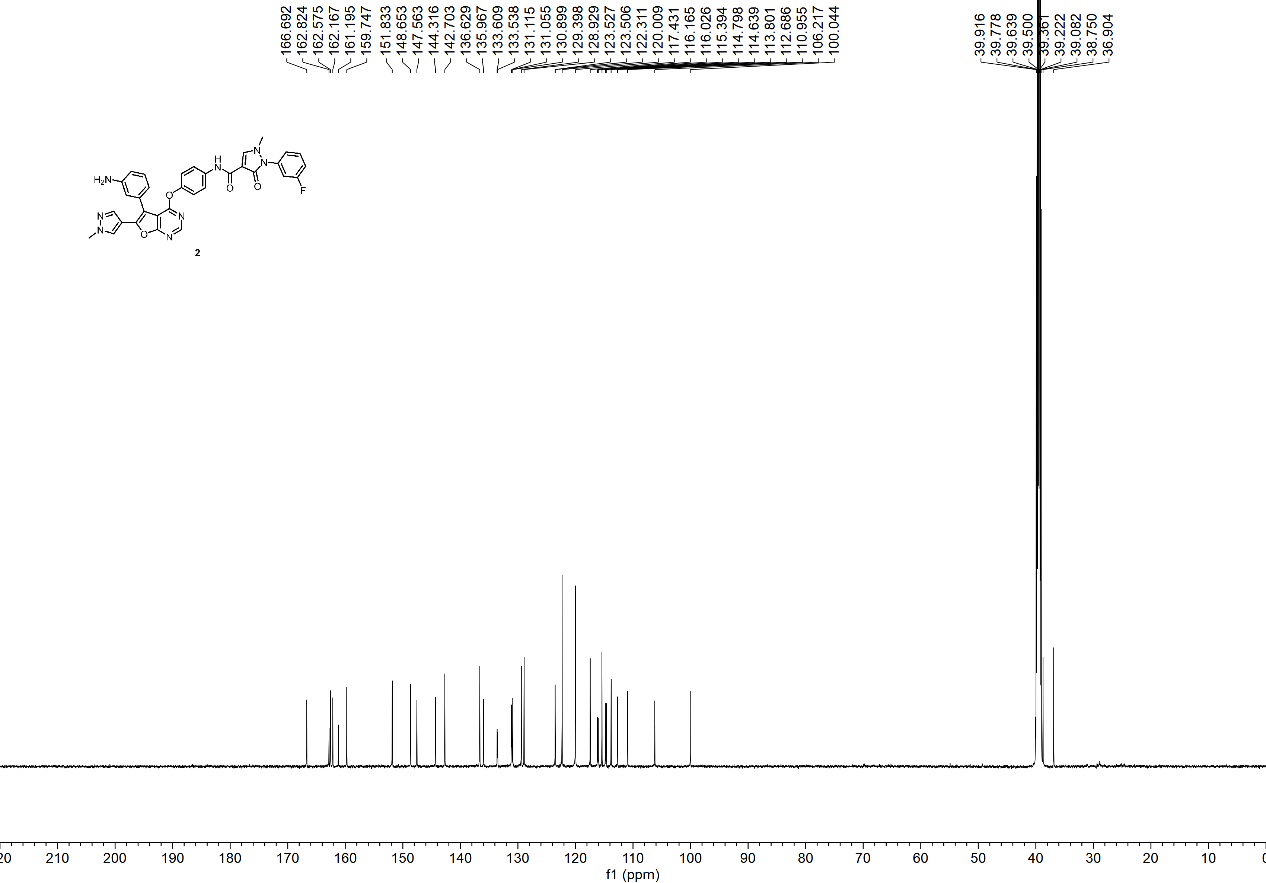


**
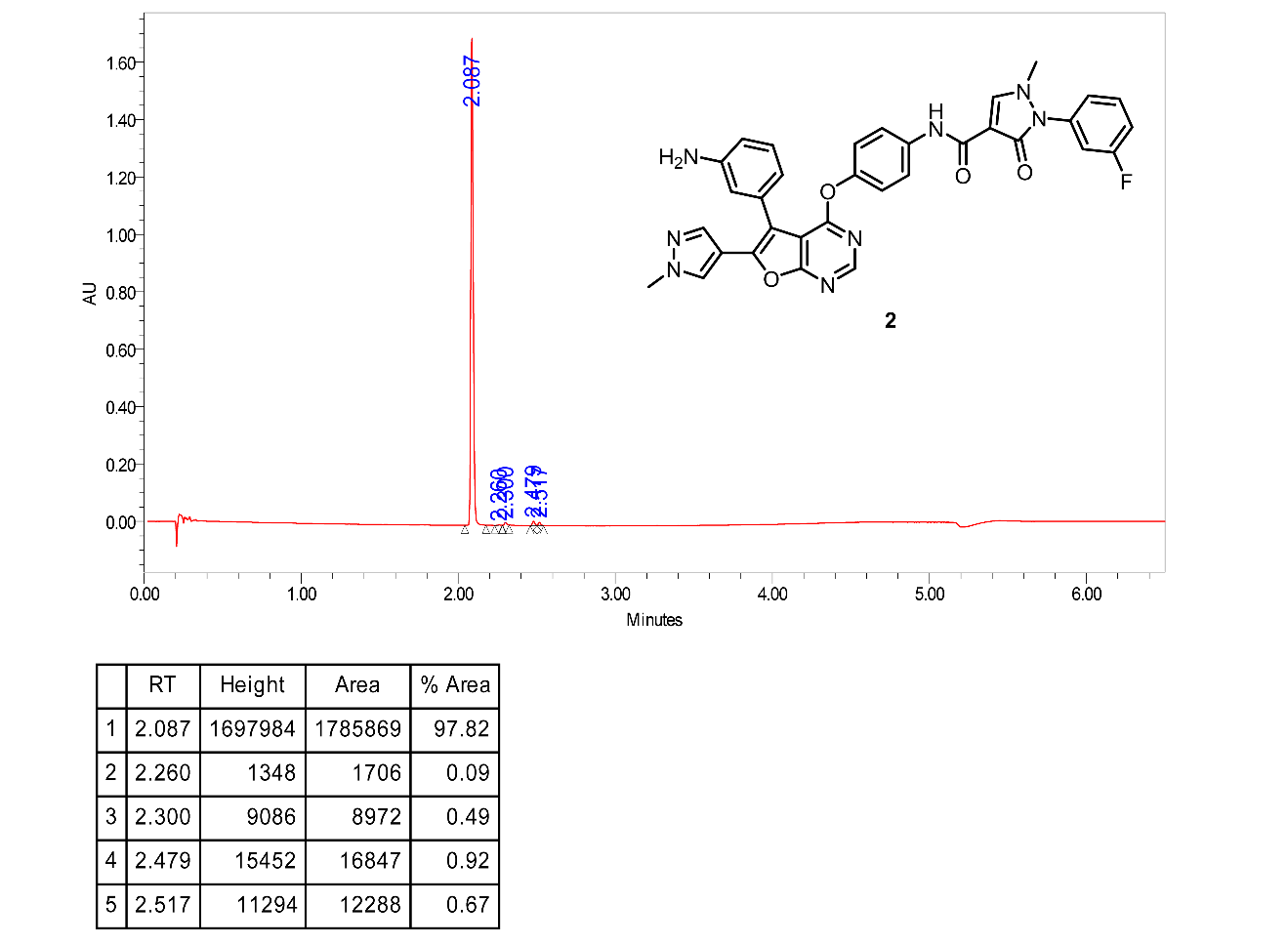
**


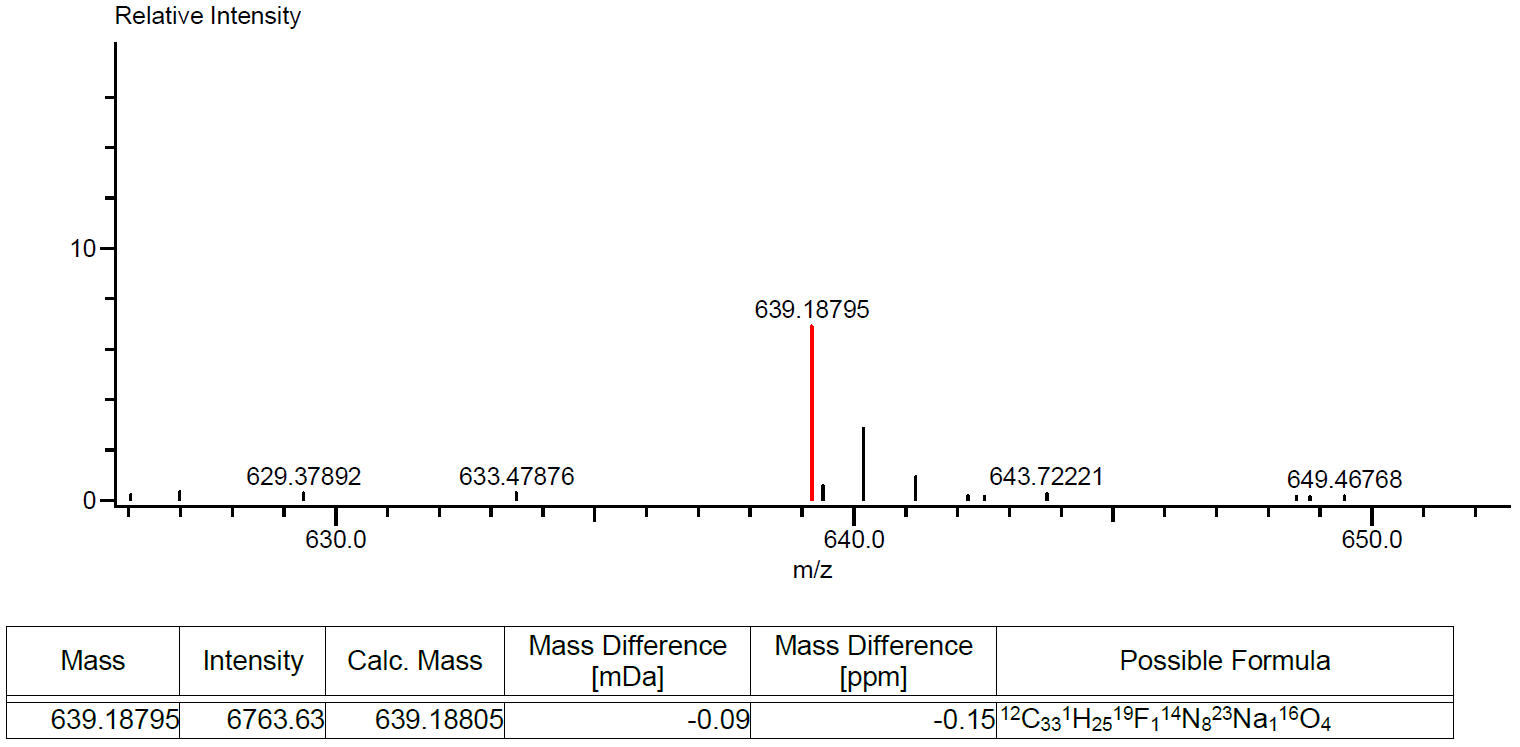


Figure S-2. ^1^H and ^13^C NMR spectra in DMSO-*d*_6_, HPLC trace, and HRMS data of compound **2**_._


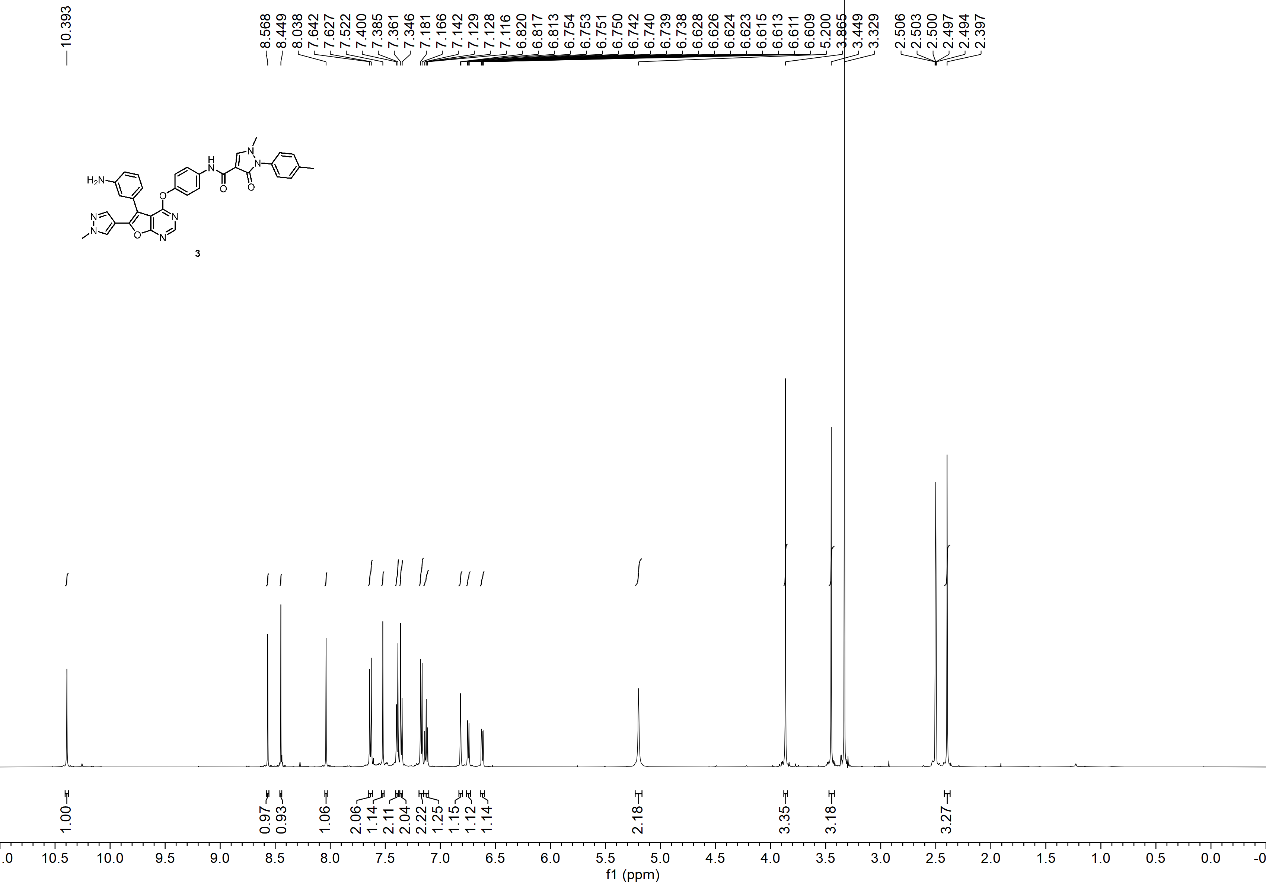


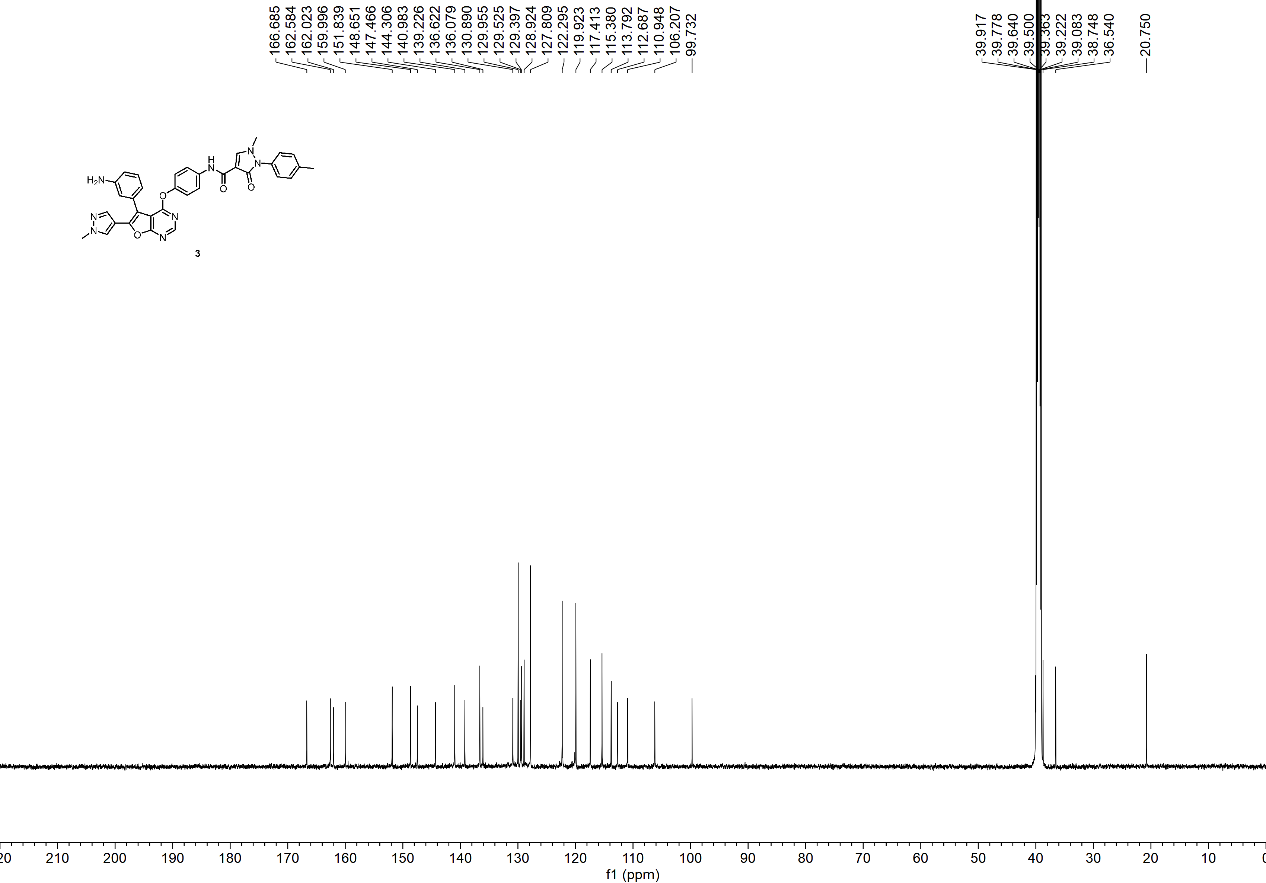


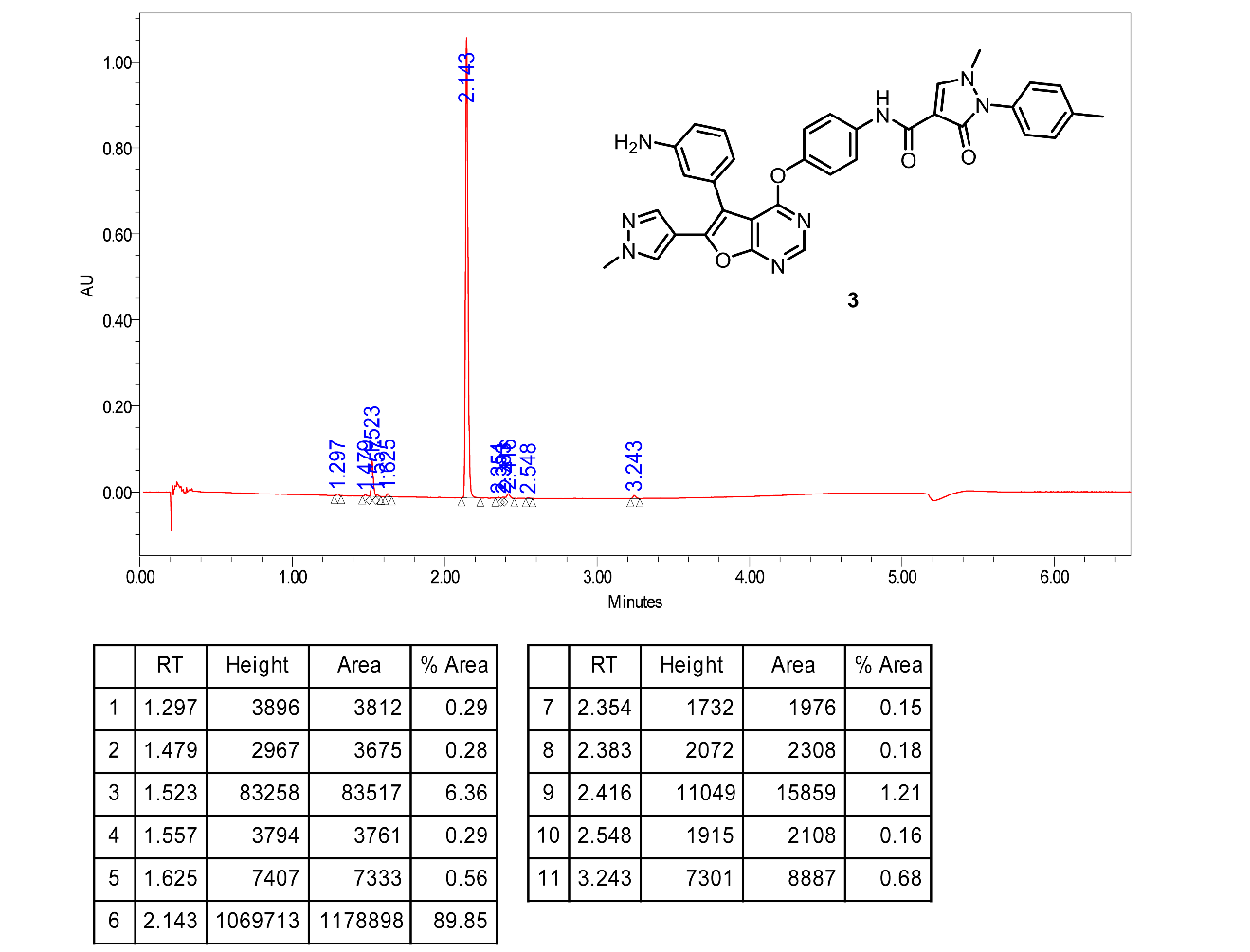


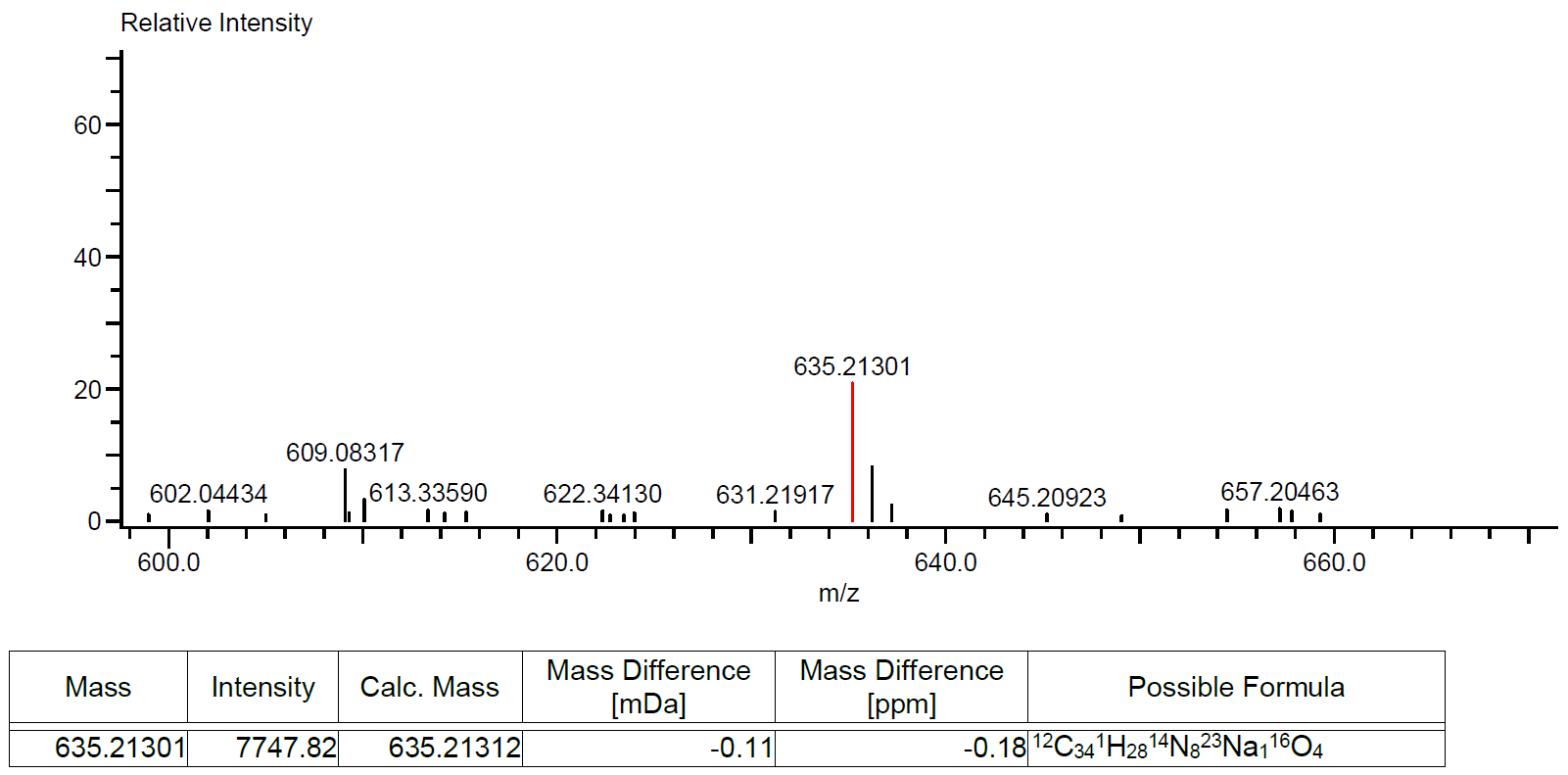


Figure S-3. ^1^H and ^13^C NMR spectra in DMSO-*d*_6_, HPLC trace, and HRMS data of compound **3**_._


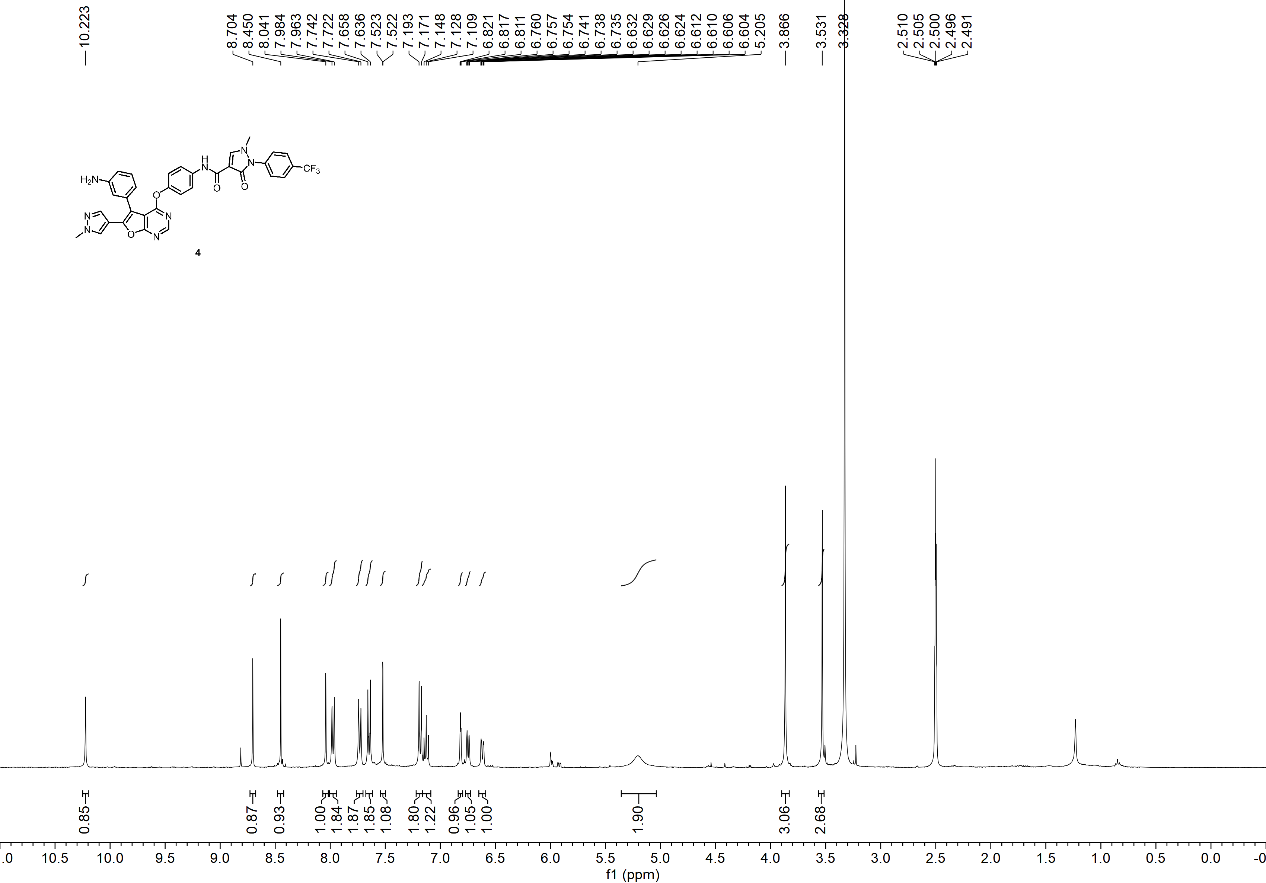


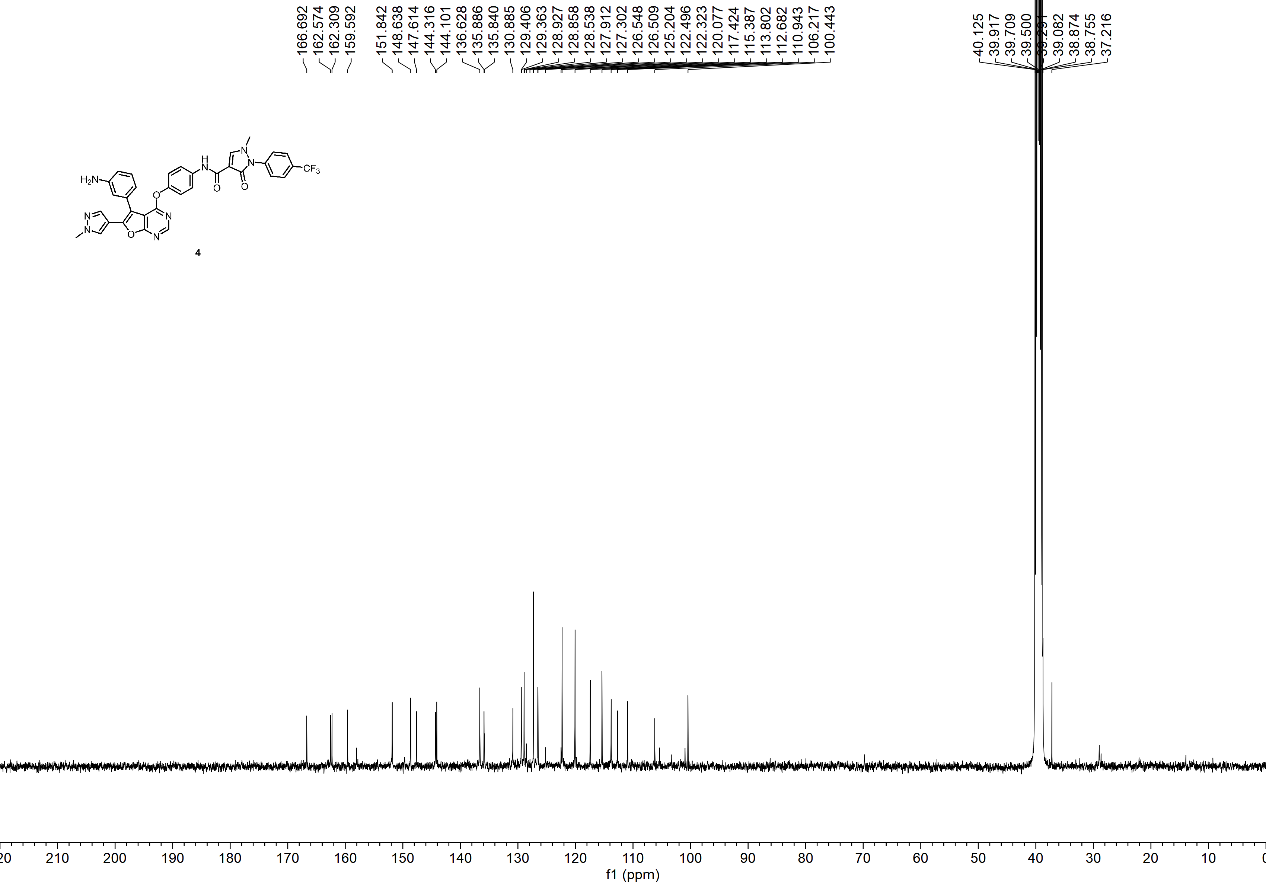


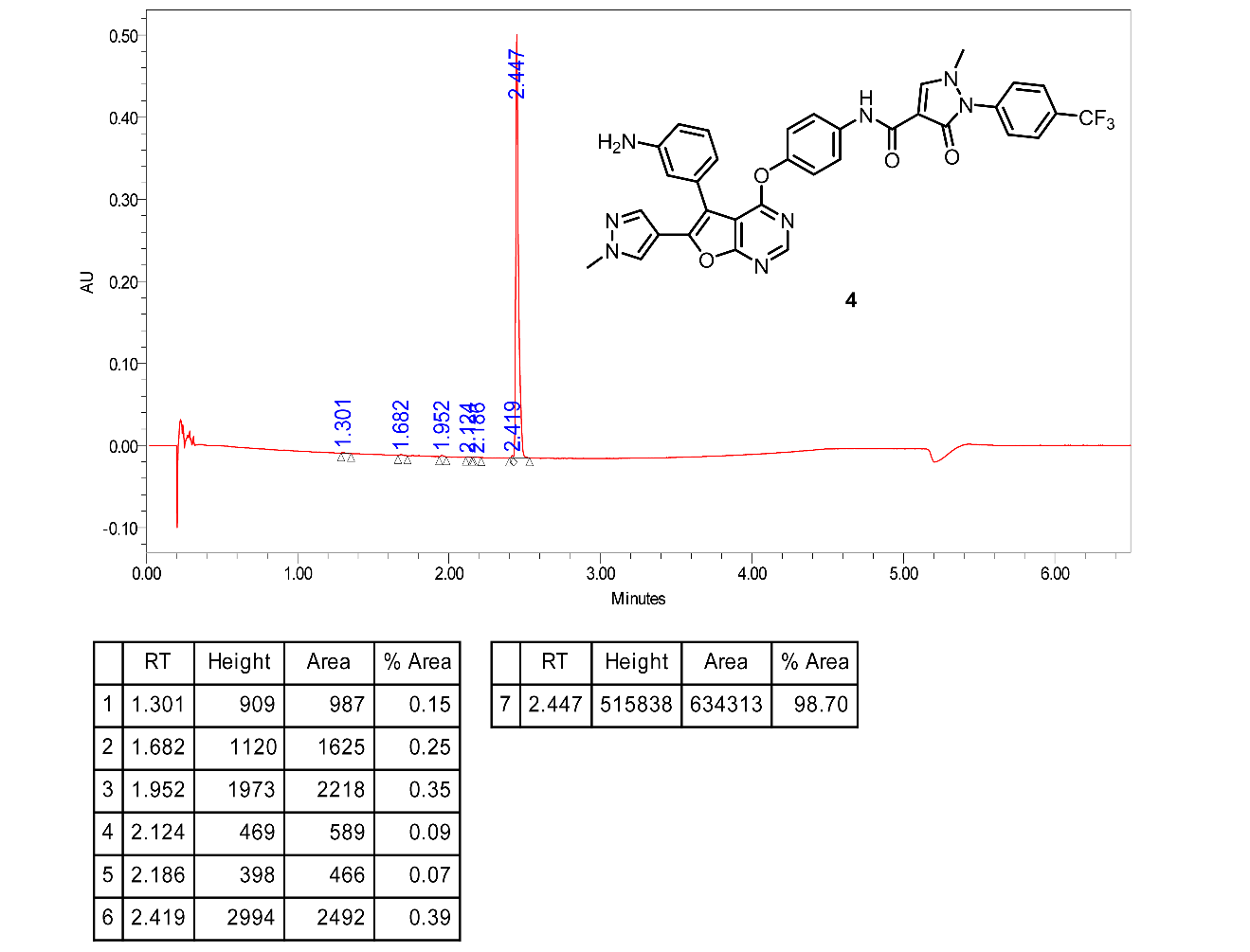


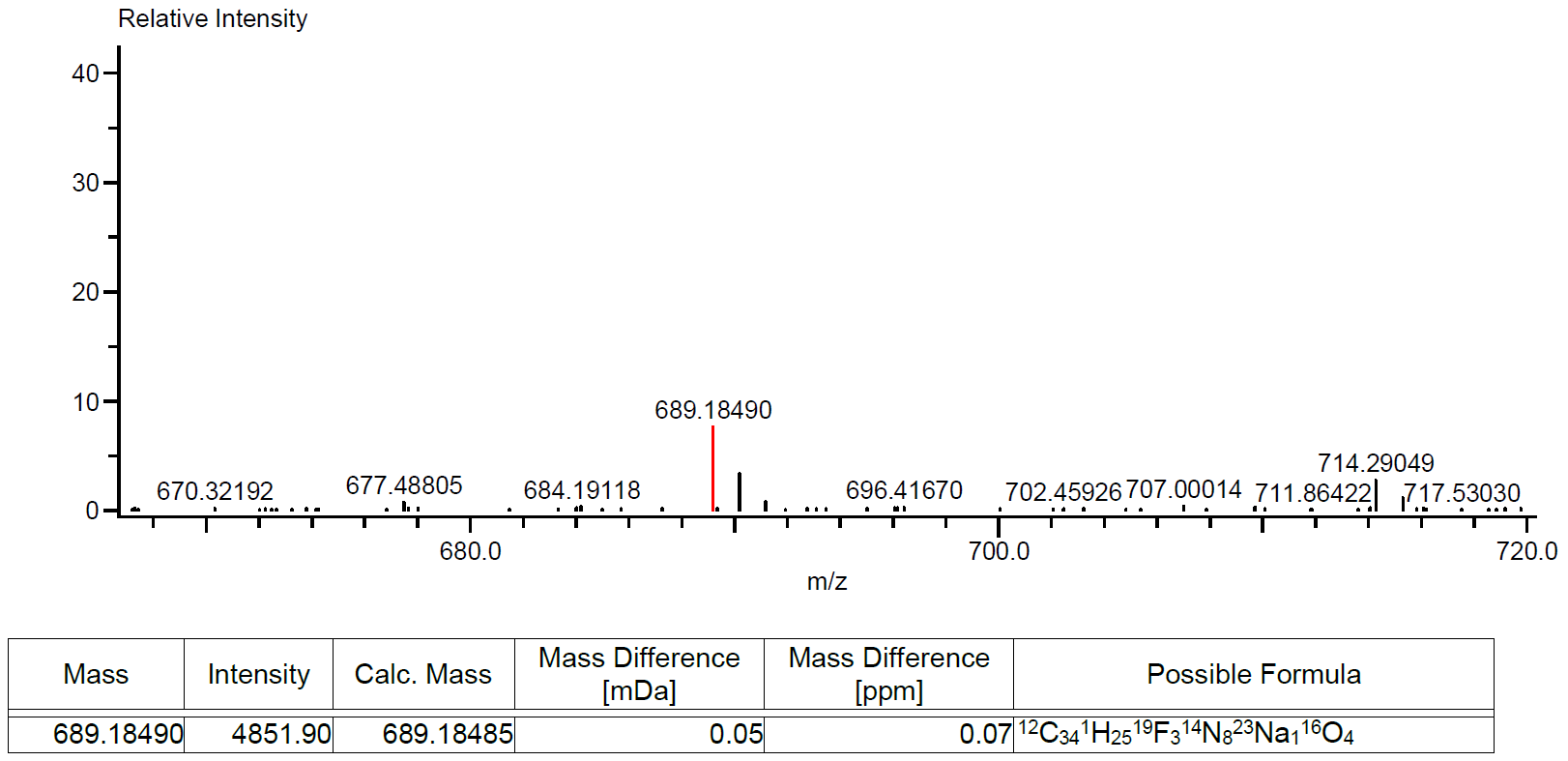


Figure S-4. ^1^H and ^13^C NMR spectra in DMSO-*d*_6_, HPLC trace, and HRMS data of compound **4**_._


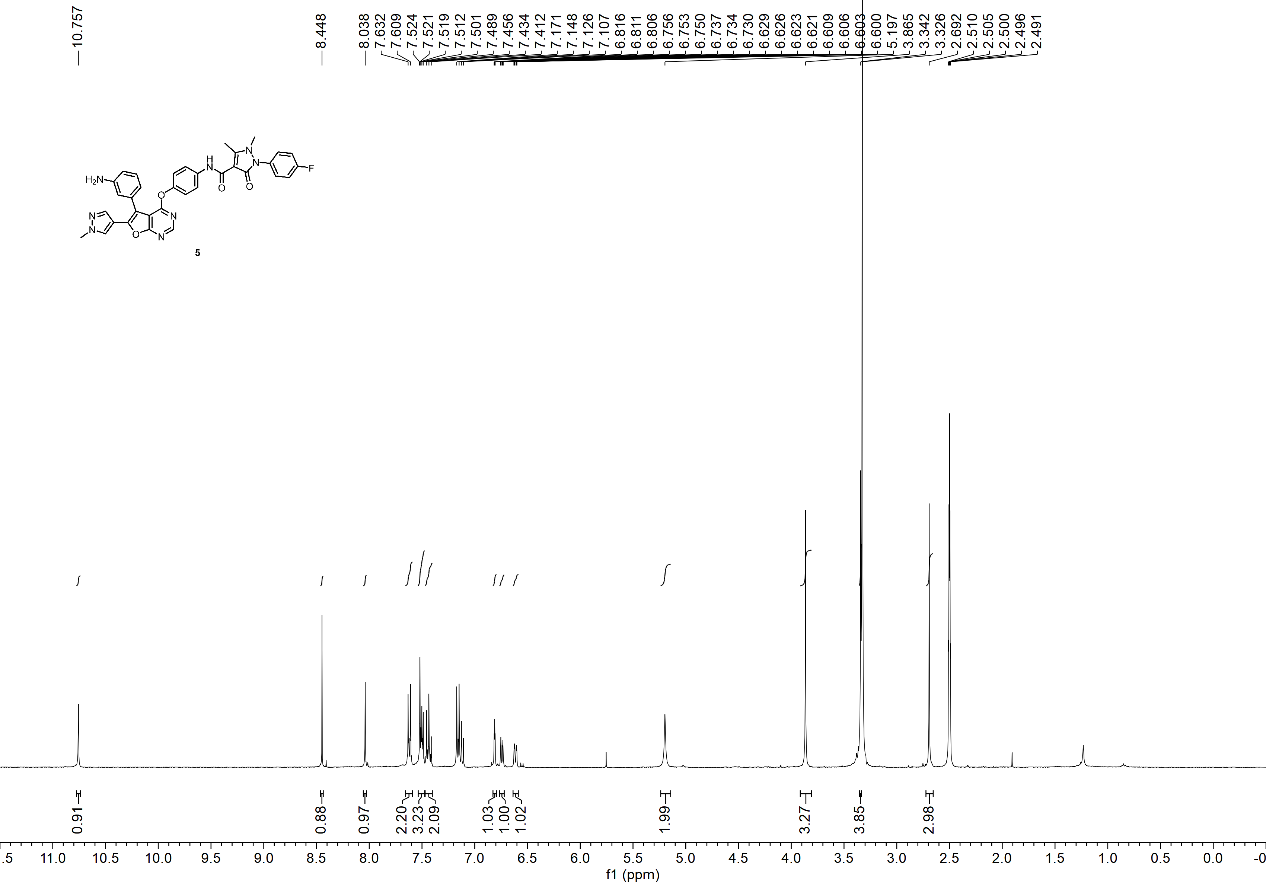


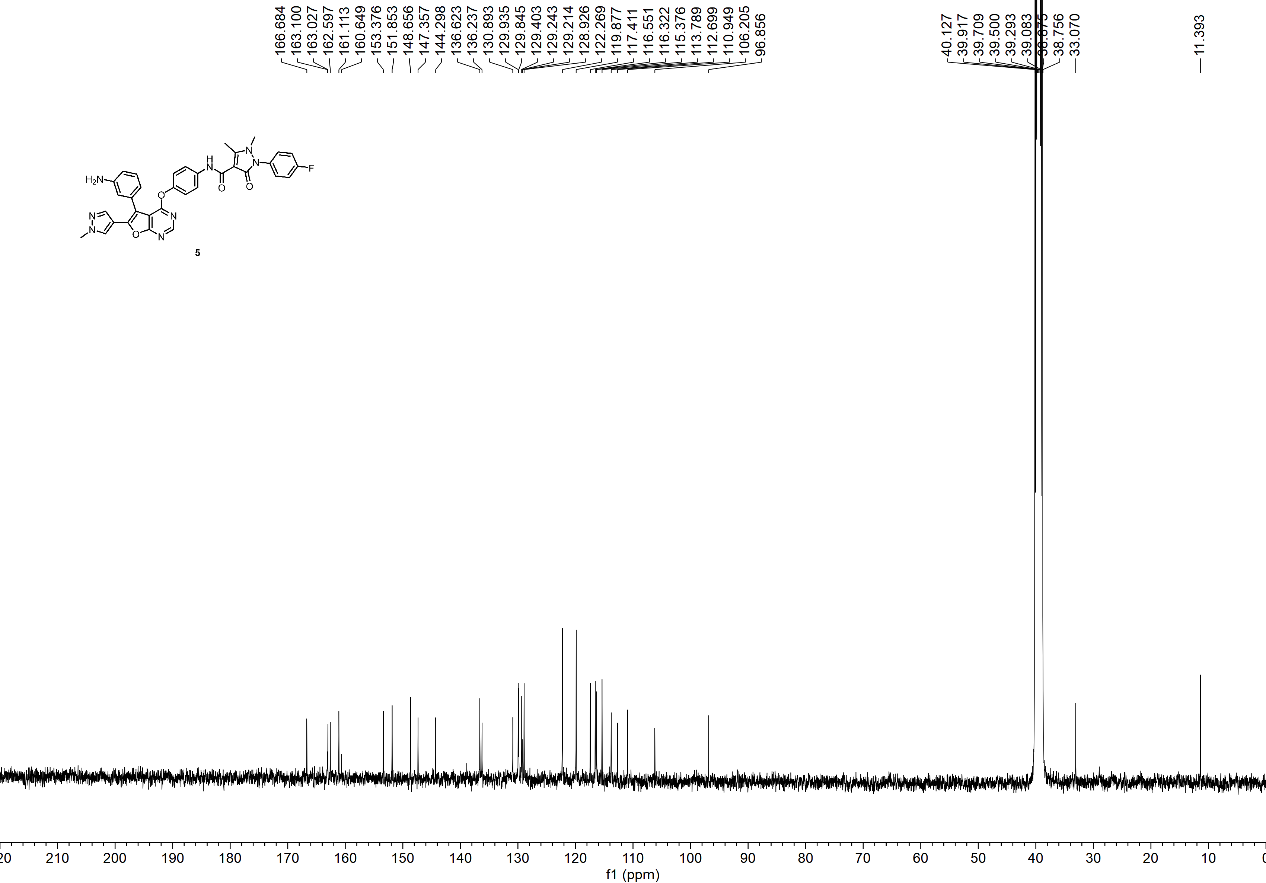


_
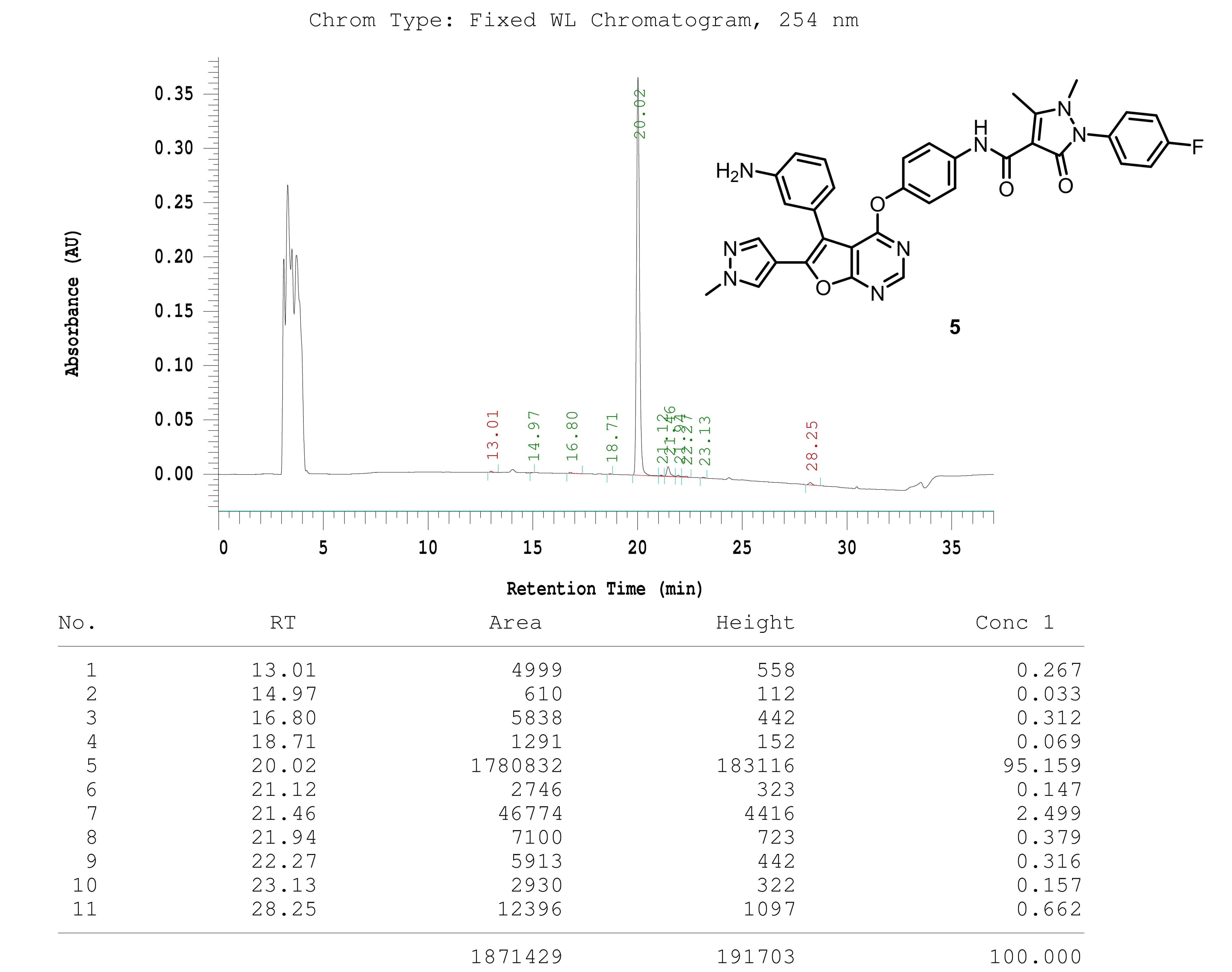
_


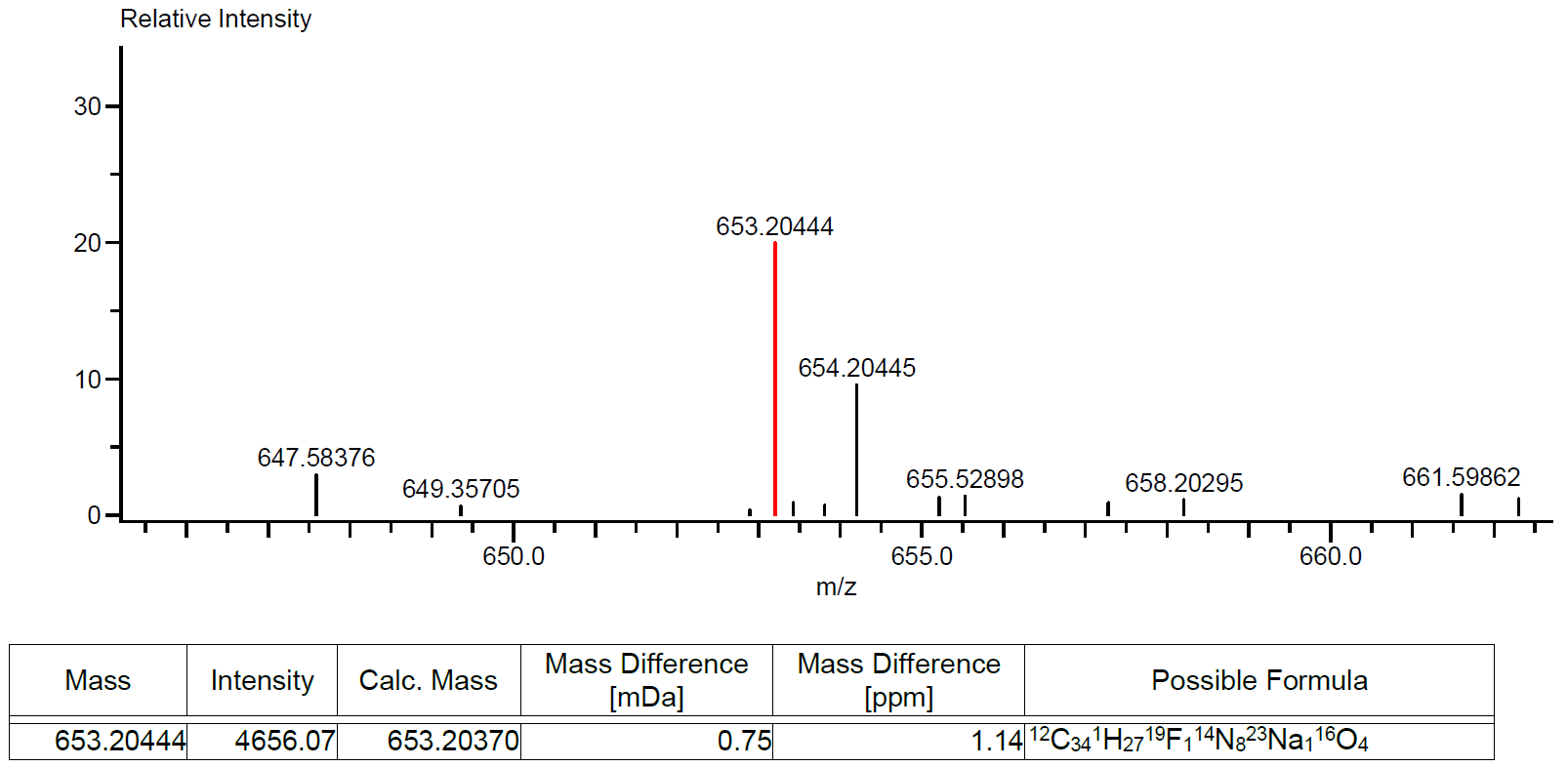


Figure S-5. ^1^H and ^13^C NMR spectra in DMSO-*d*_6_, HPLC trace, and HRMS data of compound **5**_._


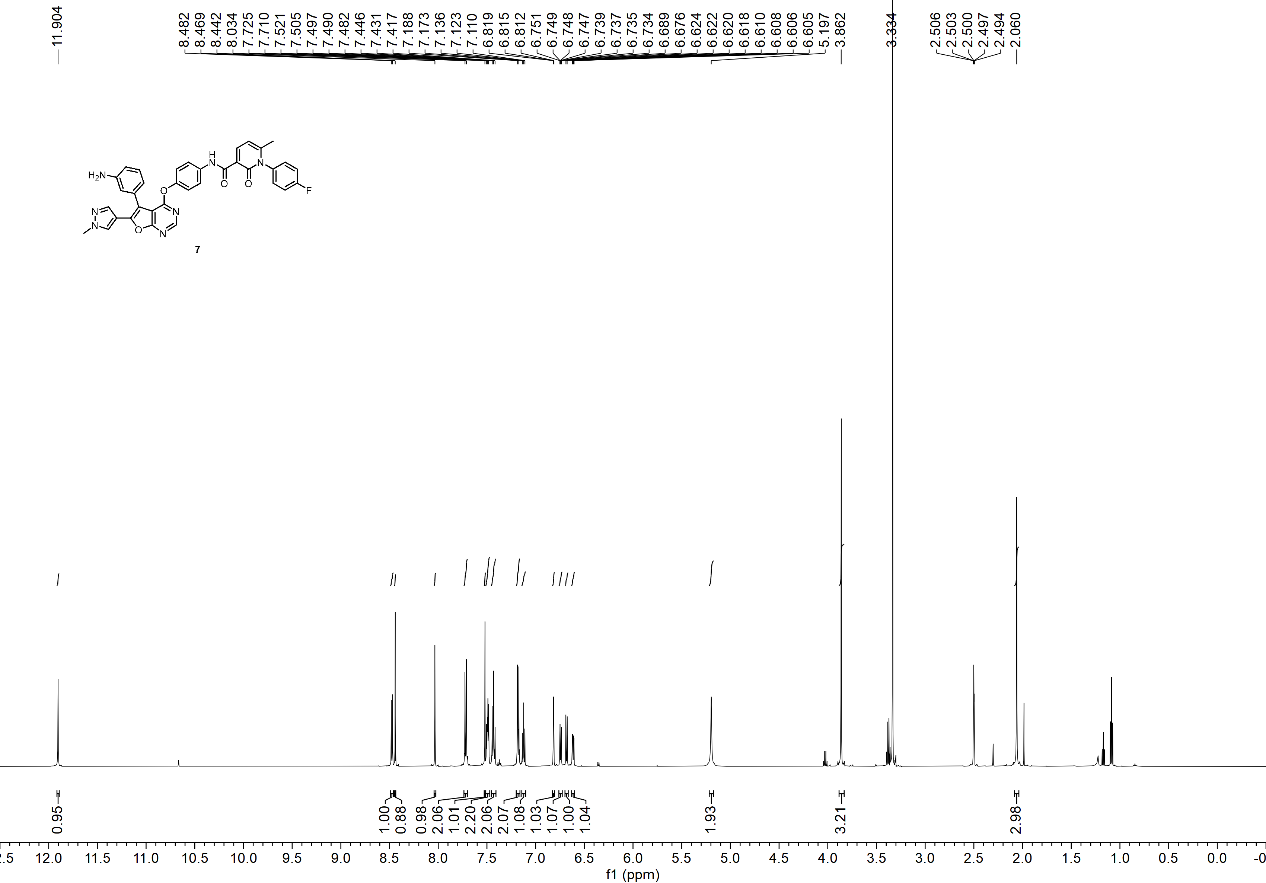


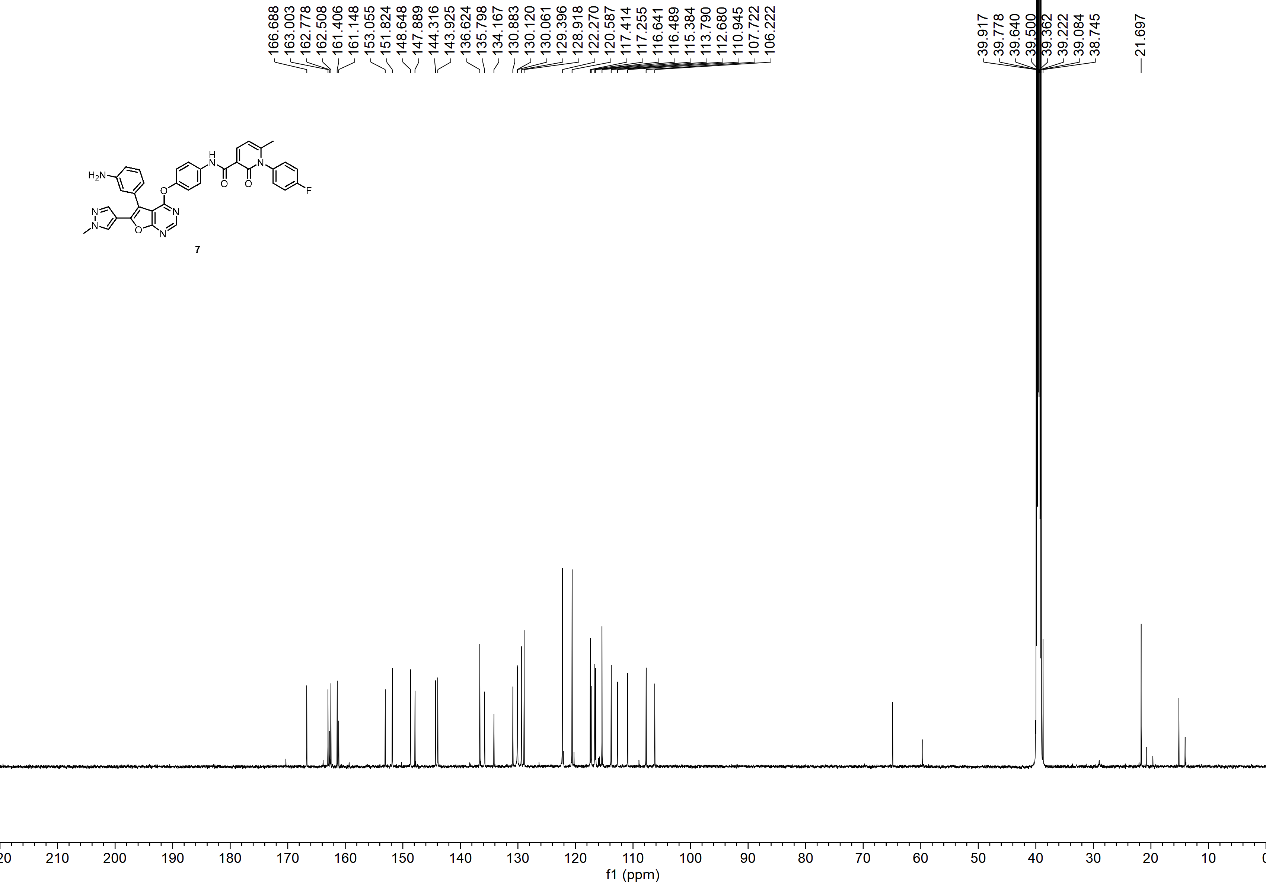


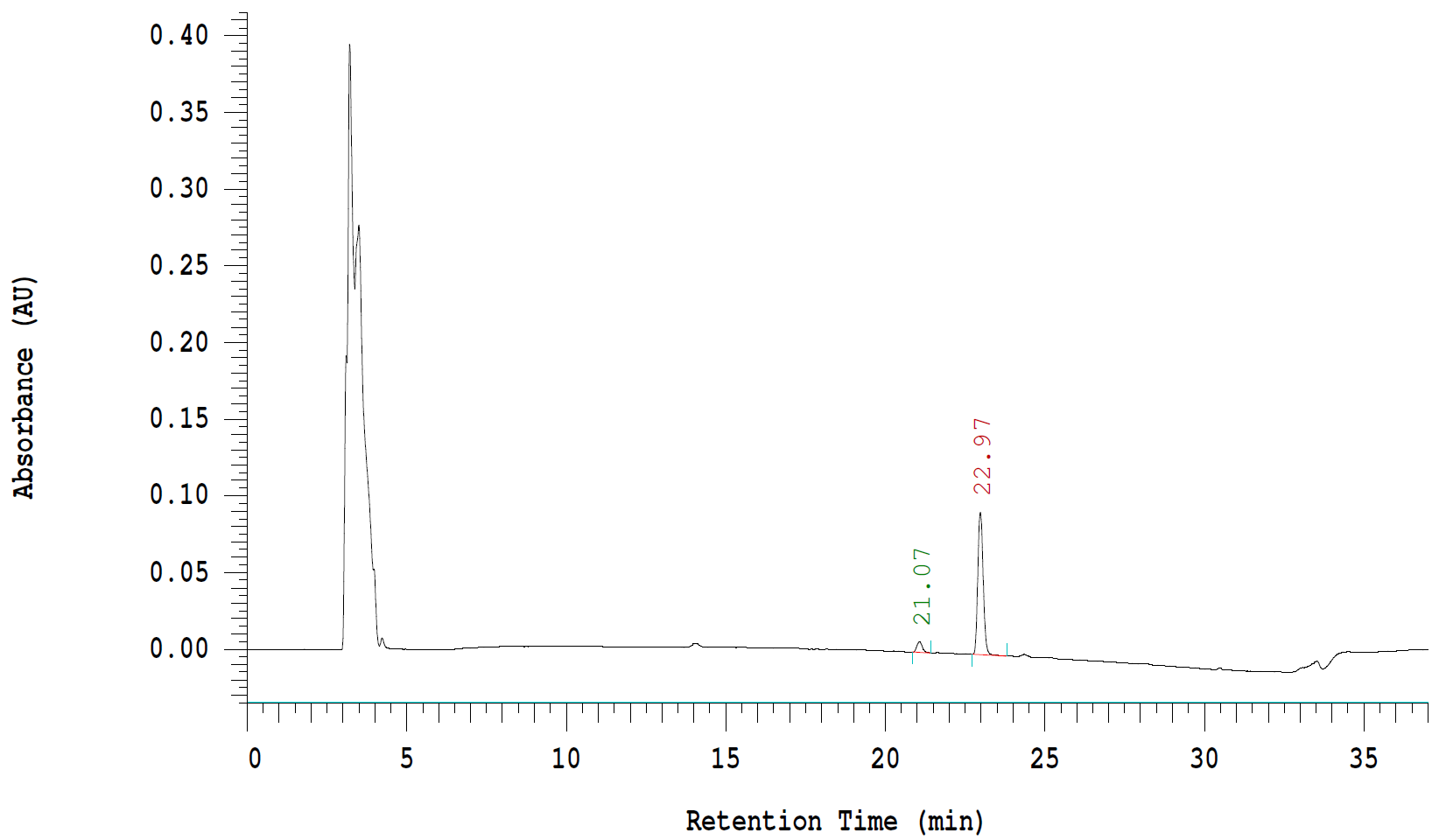


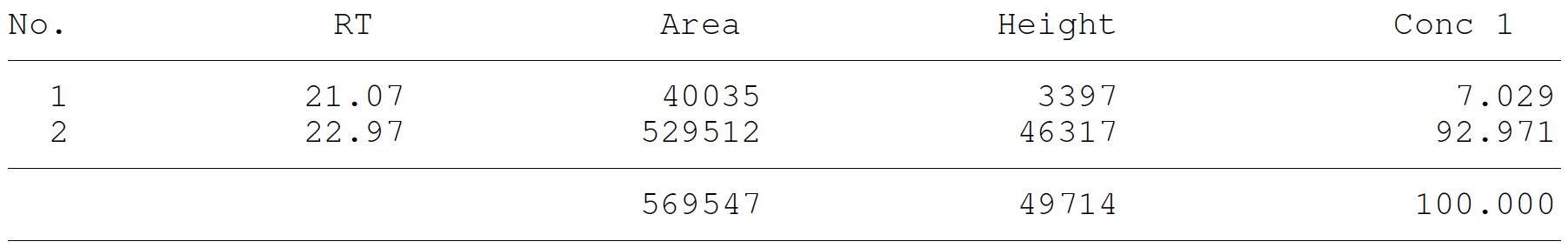


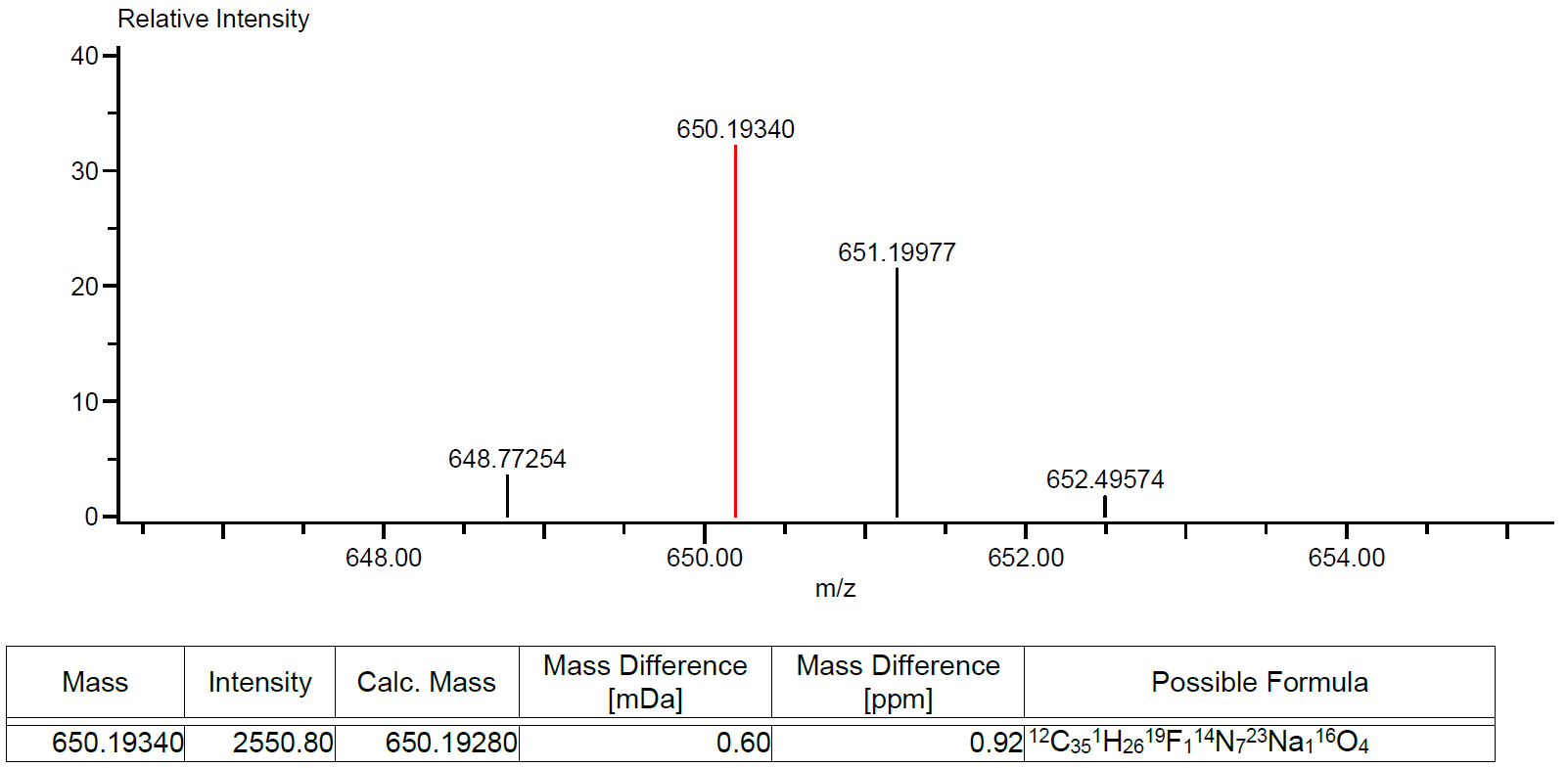


Figure S-6. ^1^H and ^13^C NMR spectra in DMSO-*d*_6_, HPLC trace, and HRMS data of compound **7**_._


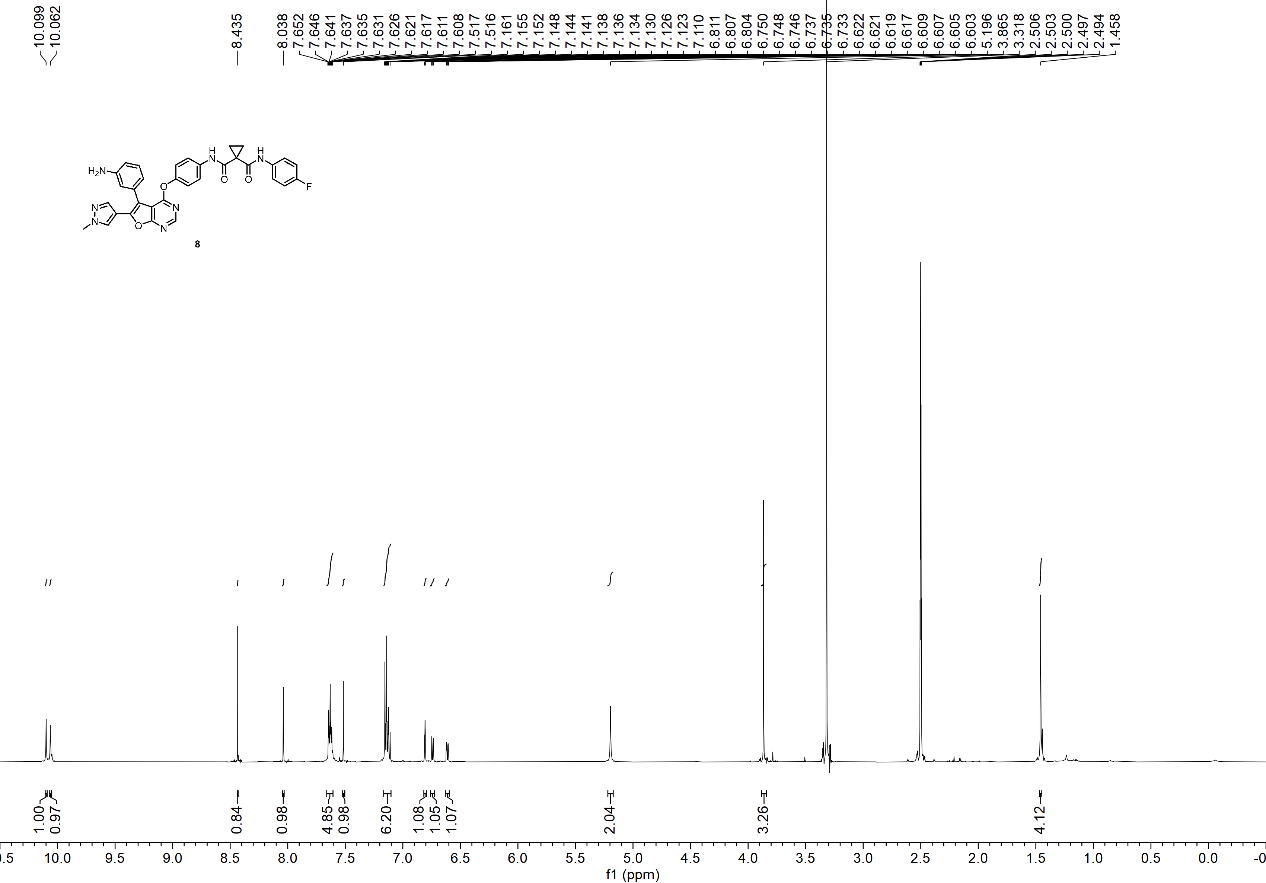


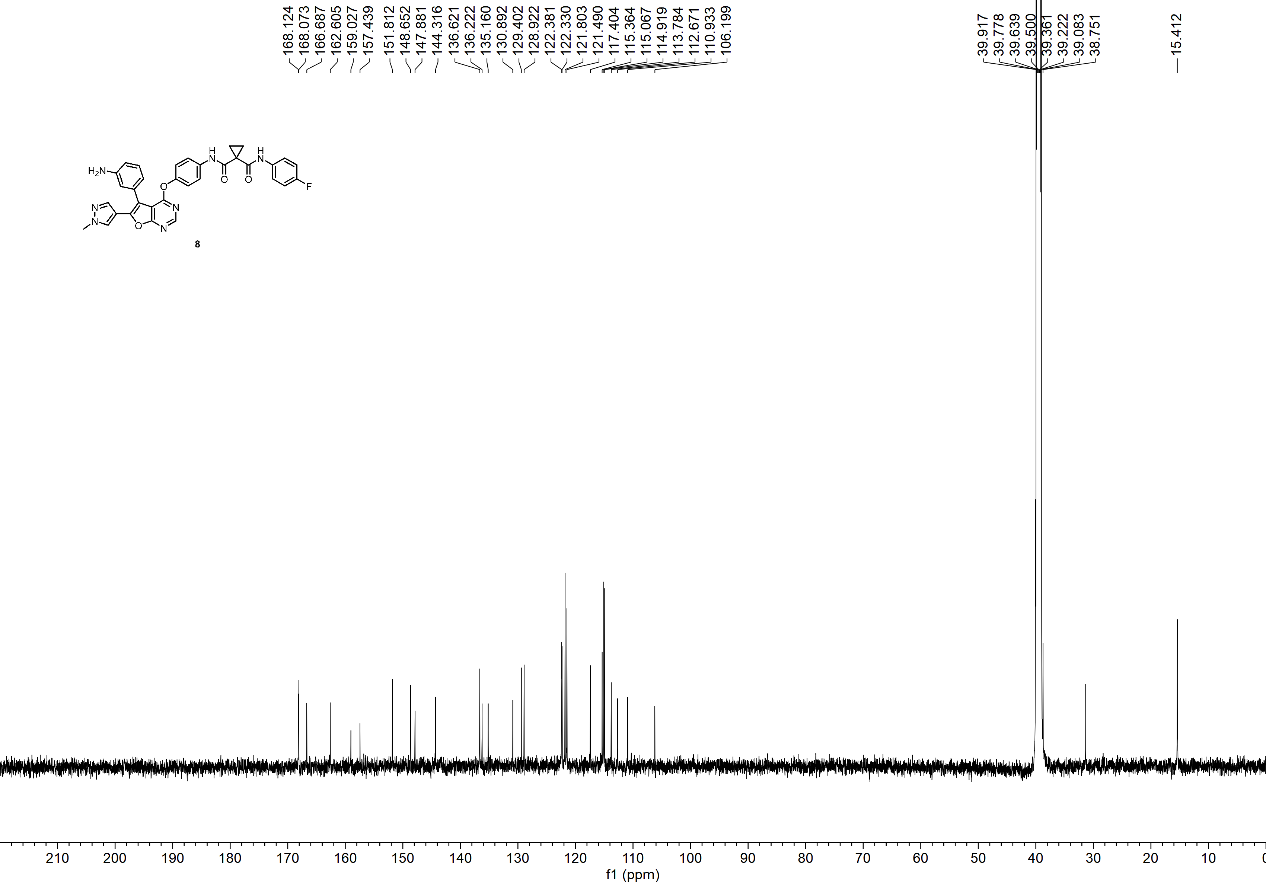


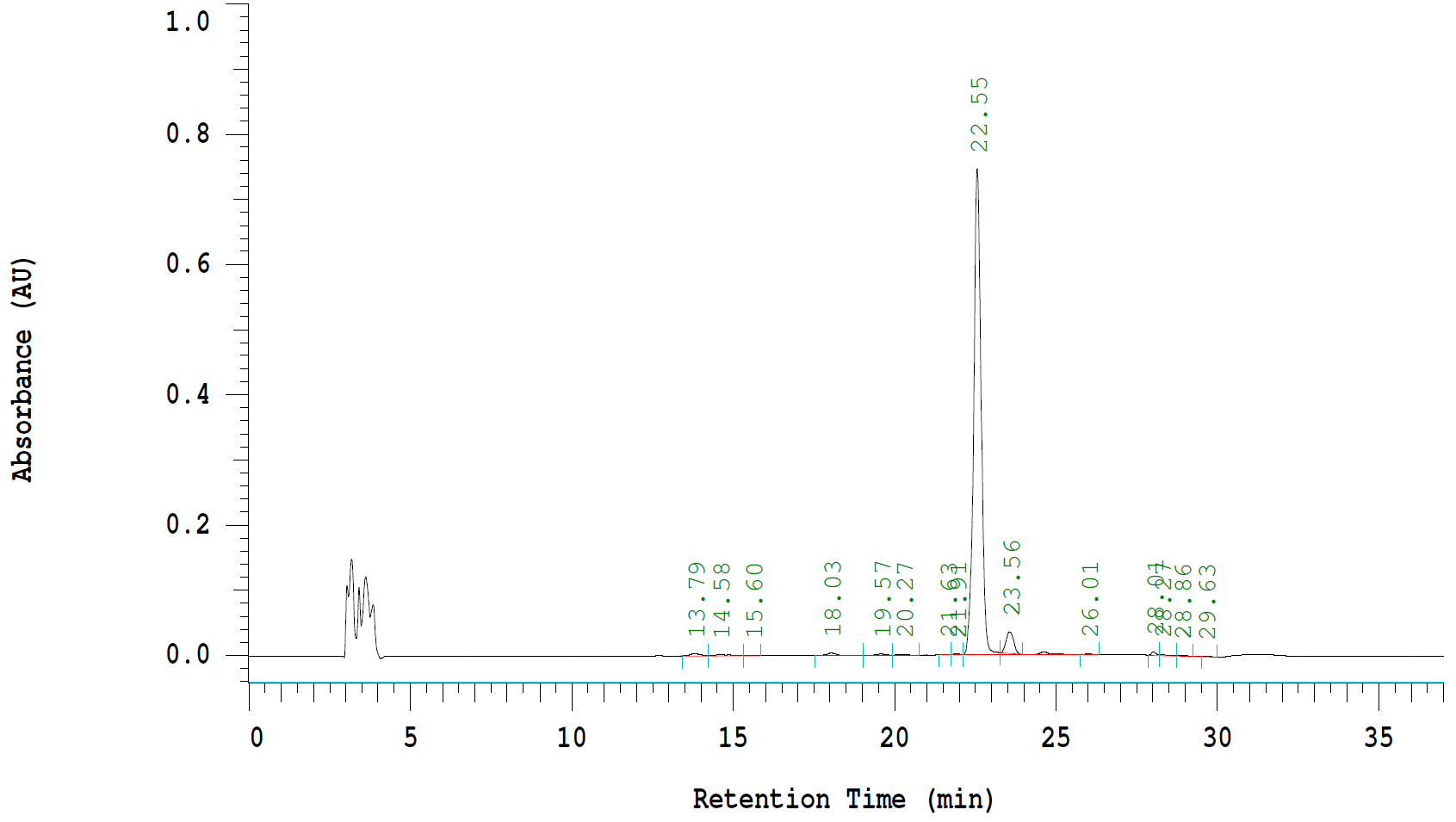

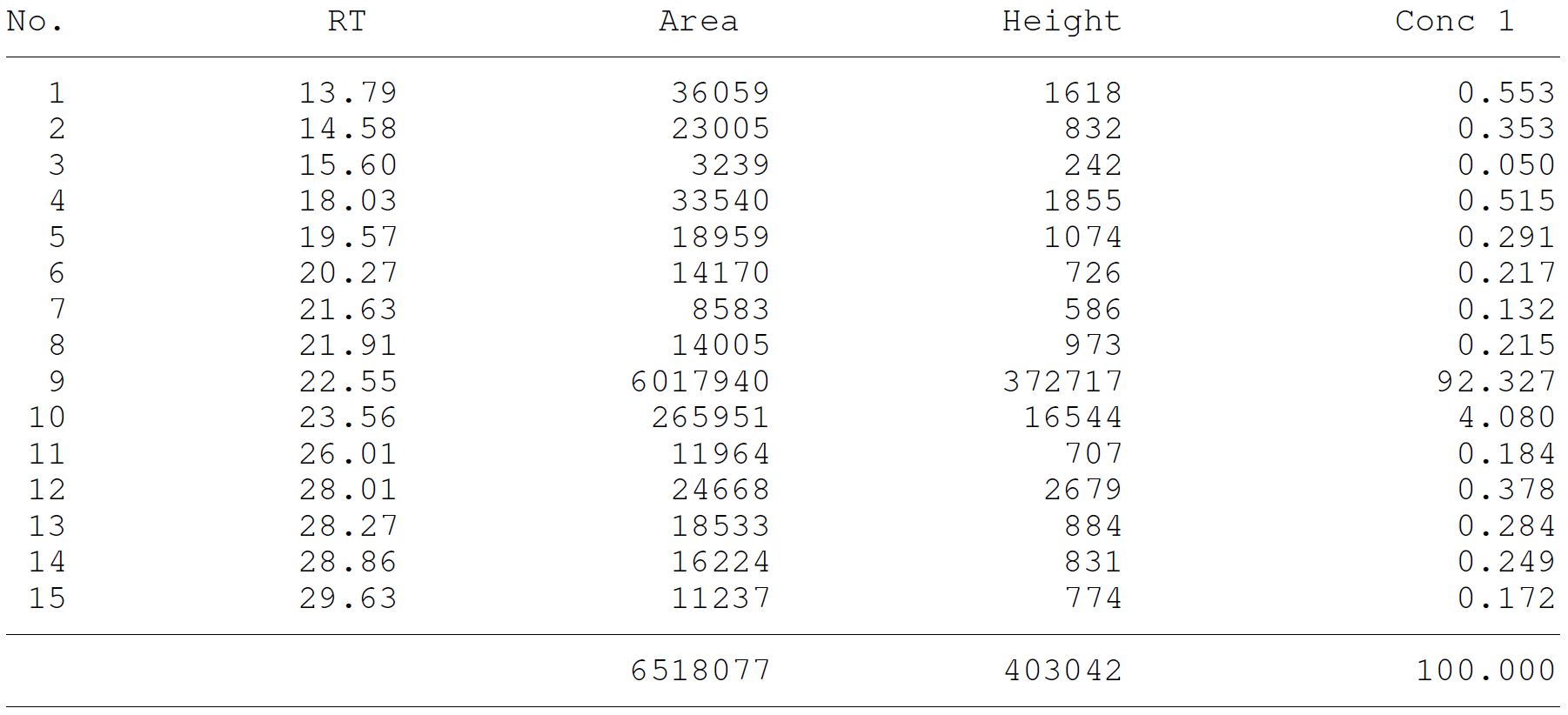


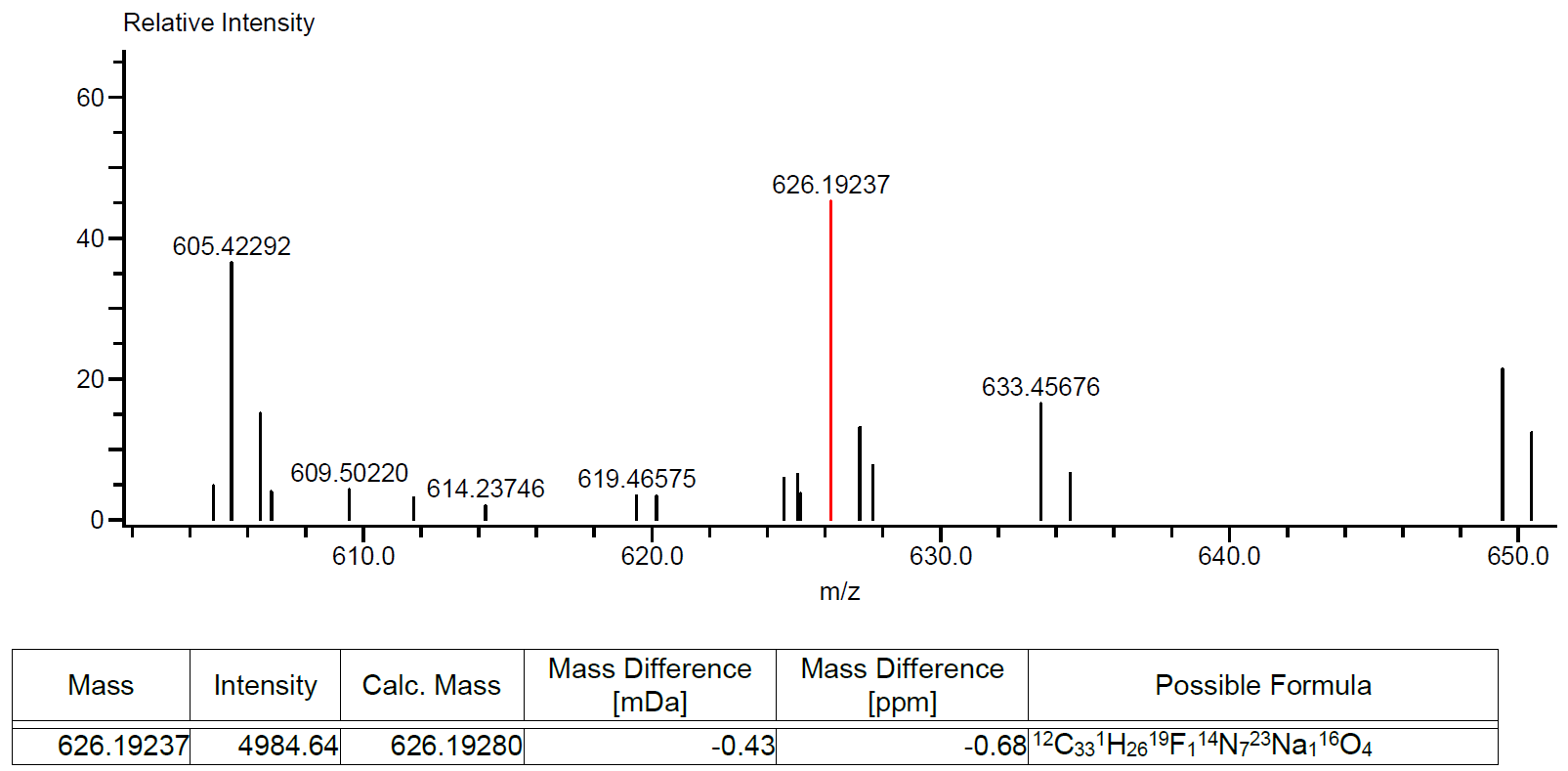


Figure S-7. ^1^H and ^13^C NMR spectra in DMSO-*d*_6_, HPLC trace, and HRMS data of compound **8**_._


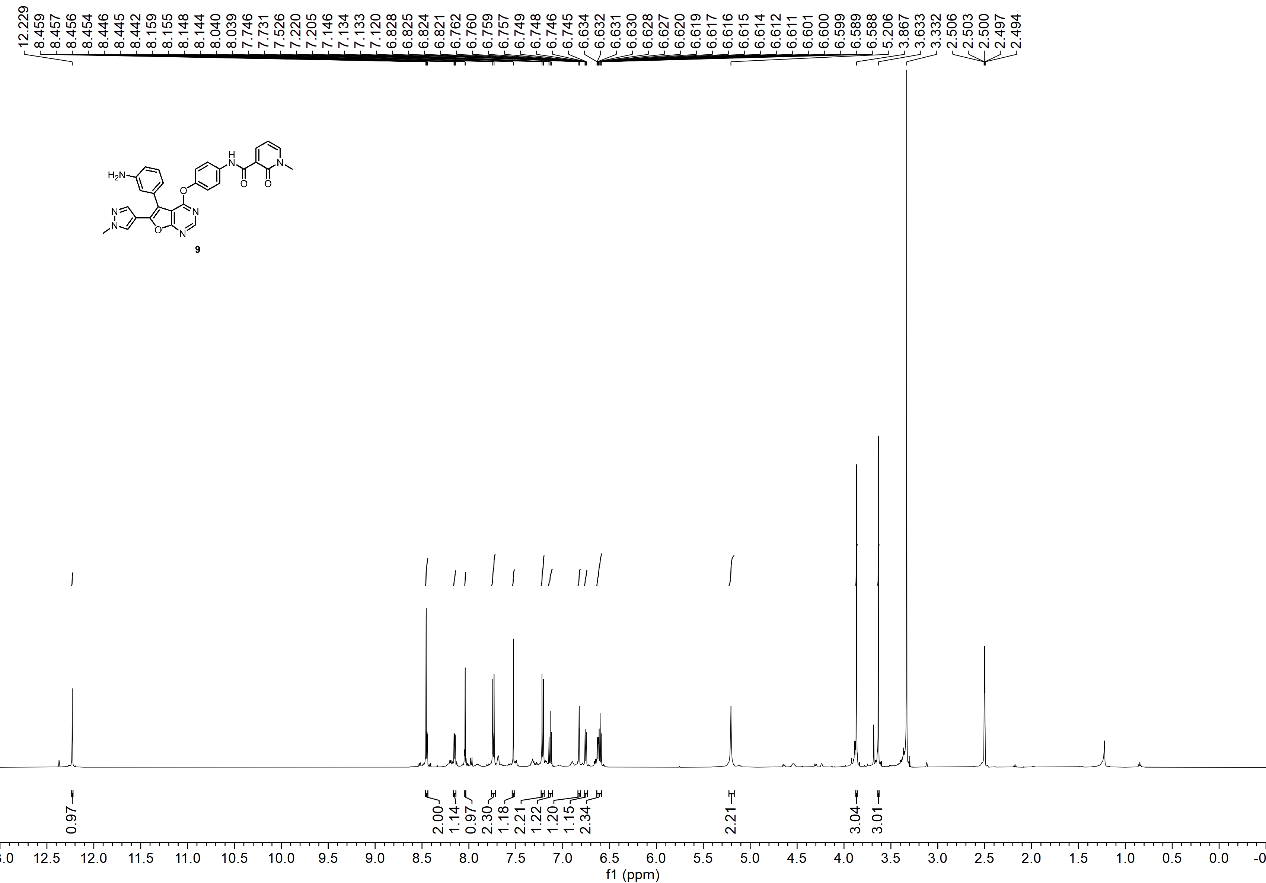


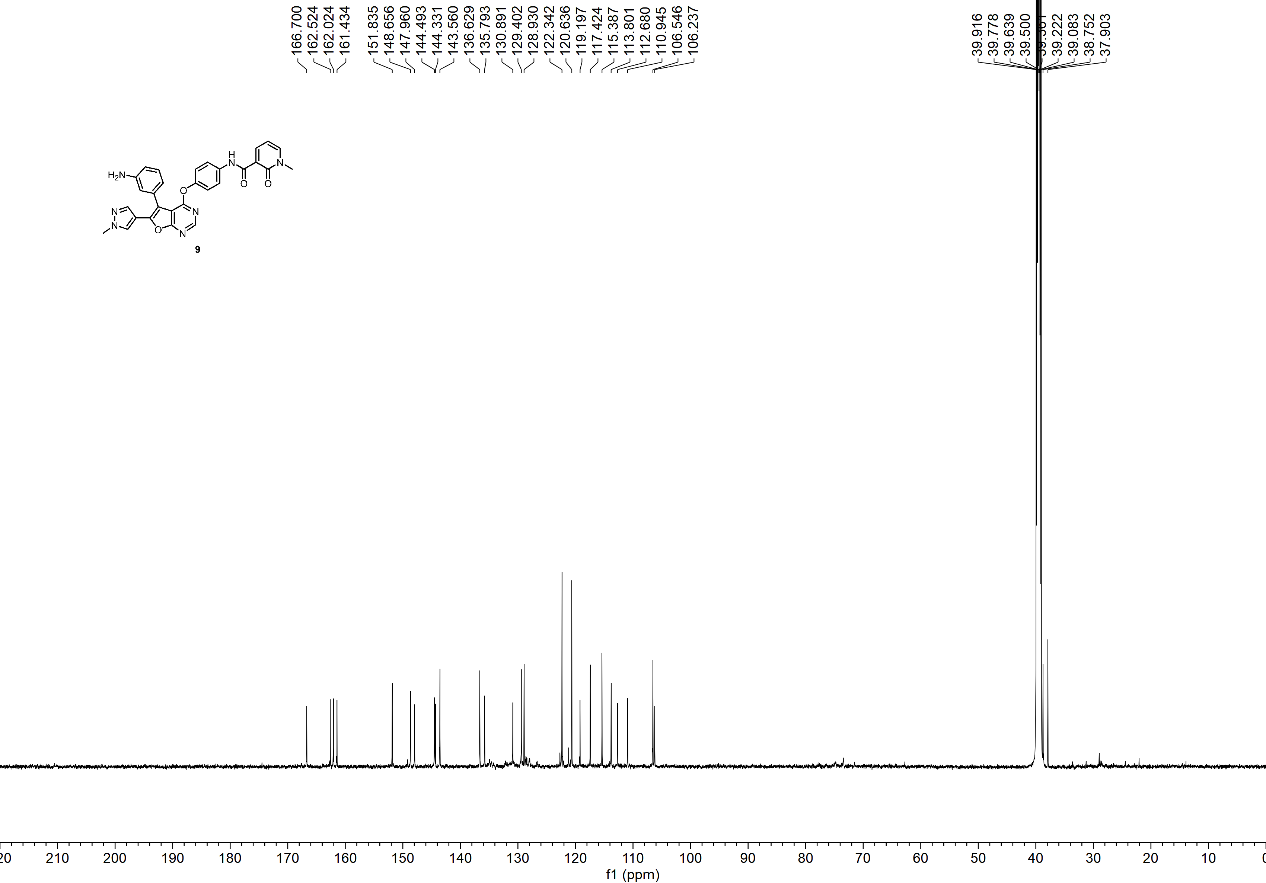


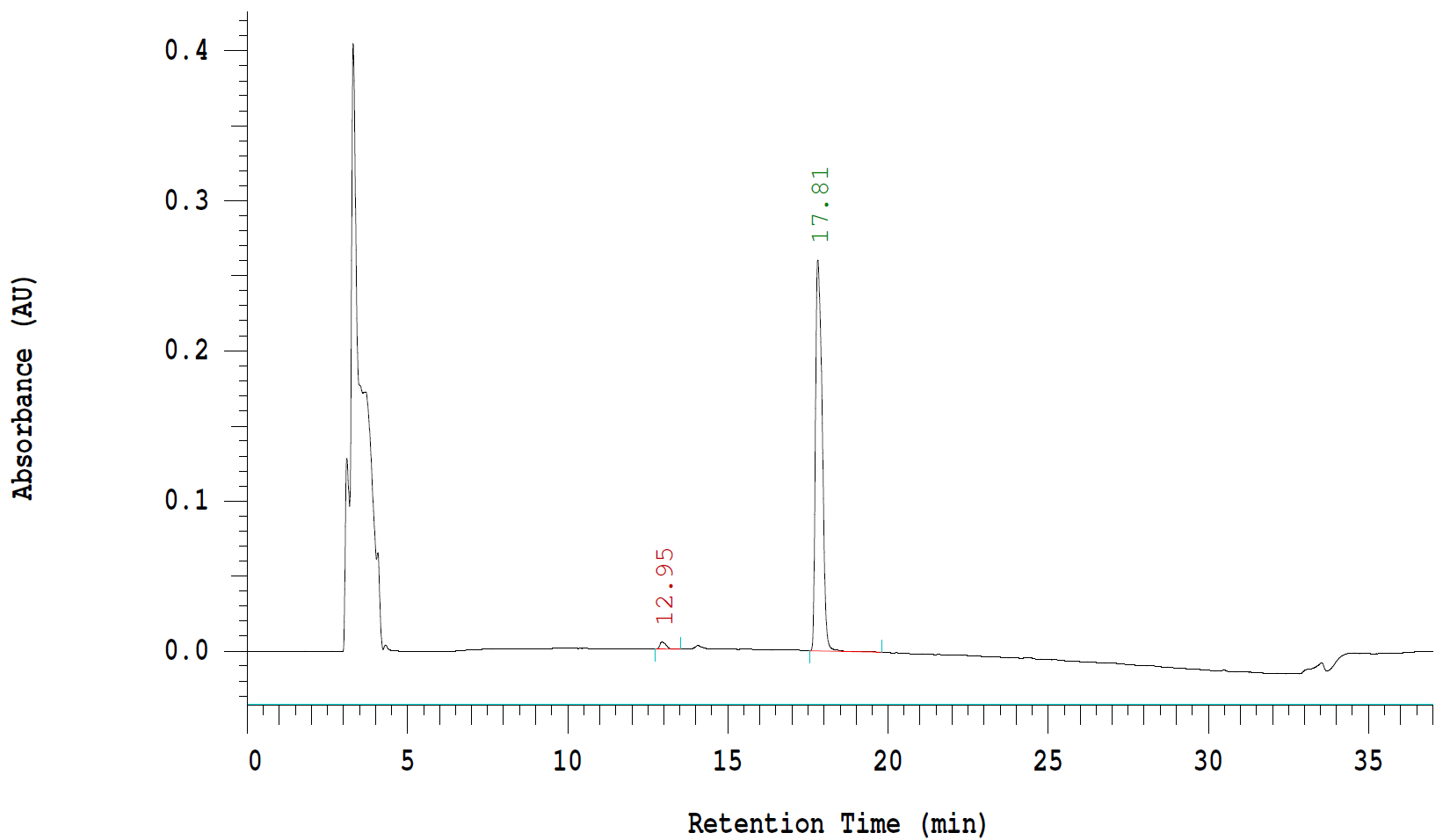


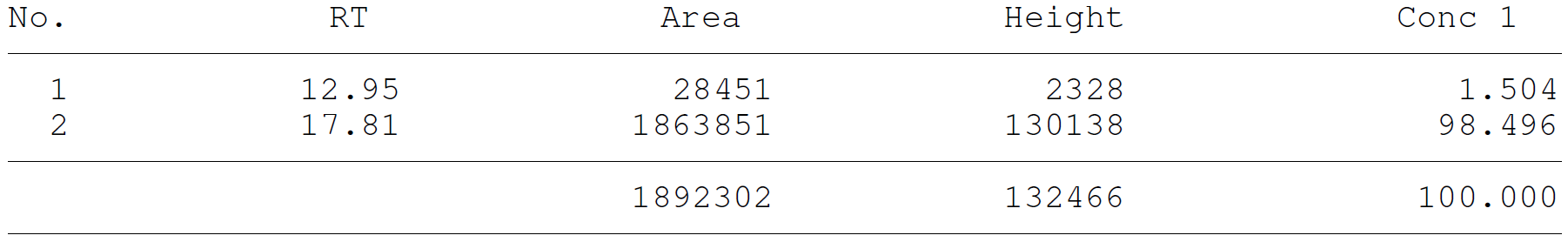


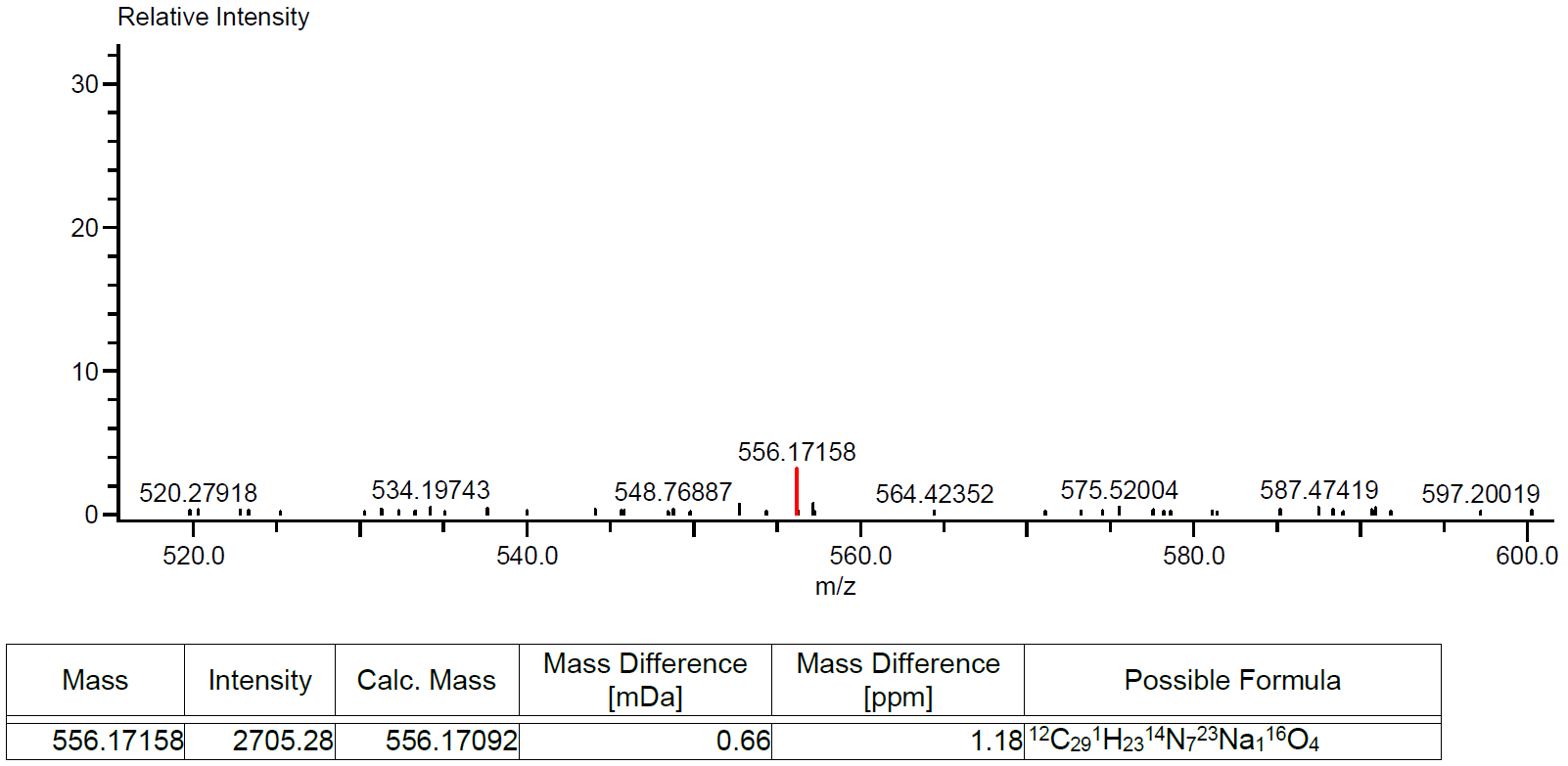


Figure S-8. ^1^H and ^13^C NMR spectra in DMSO-*d*_6_, HPLC trace, and HRMS data of compound **9**_._


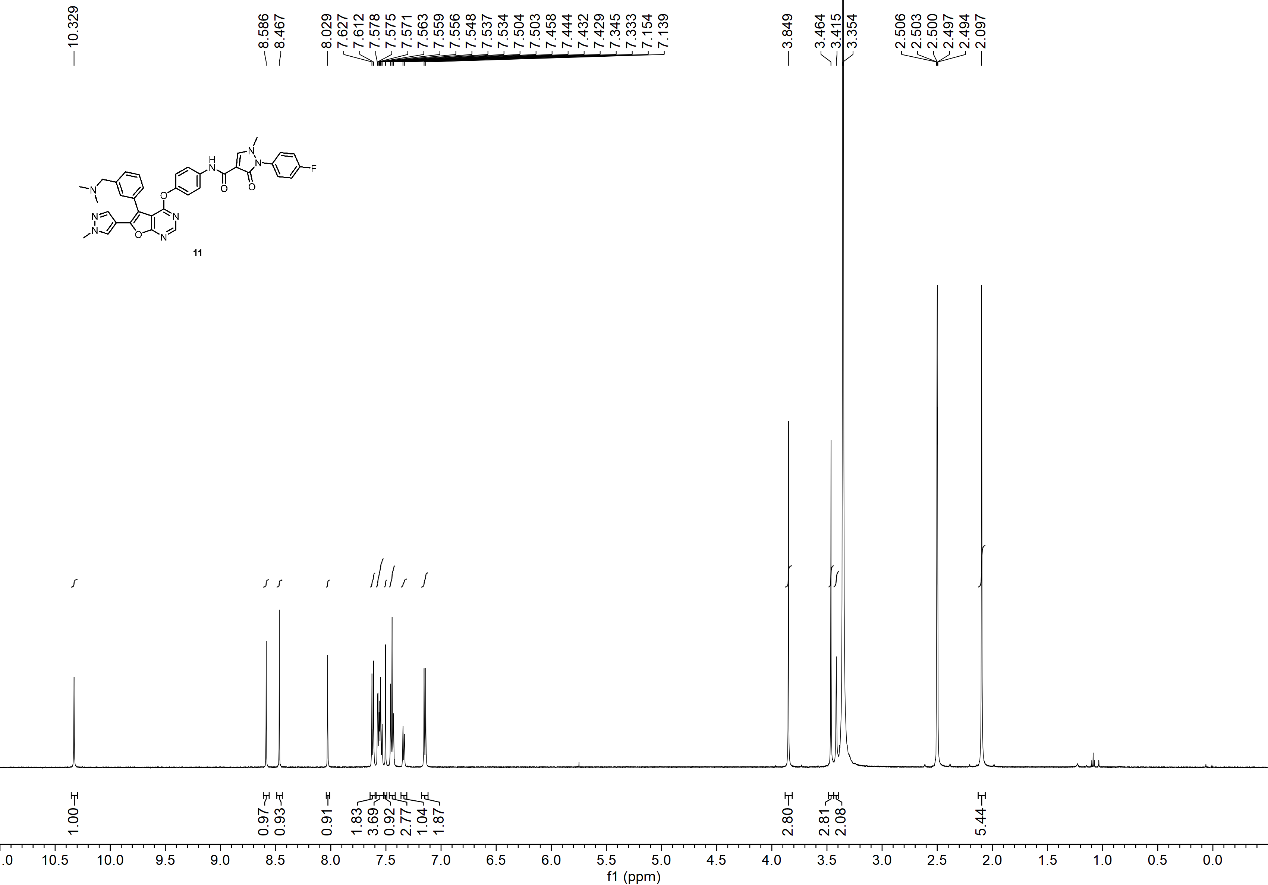

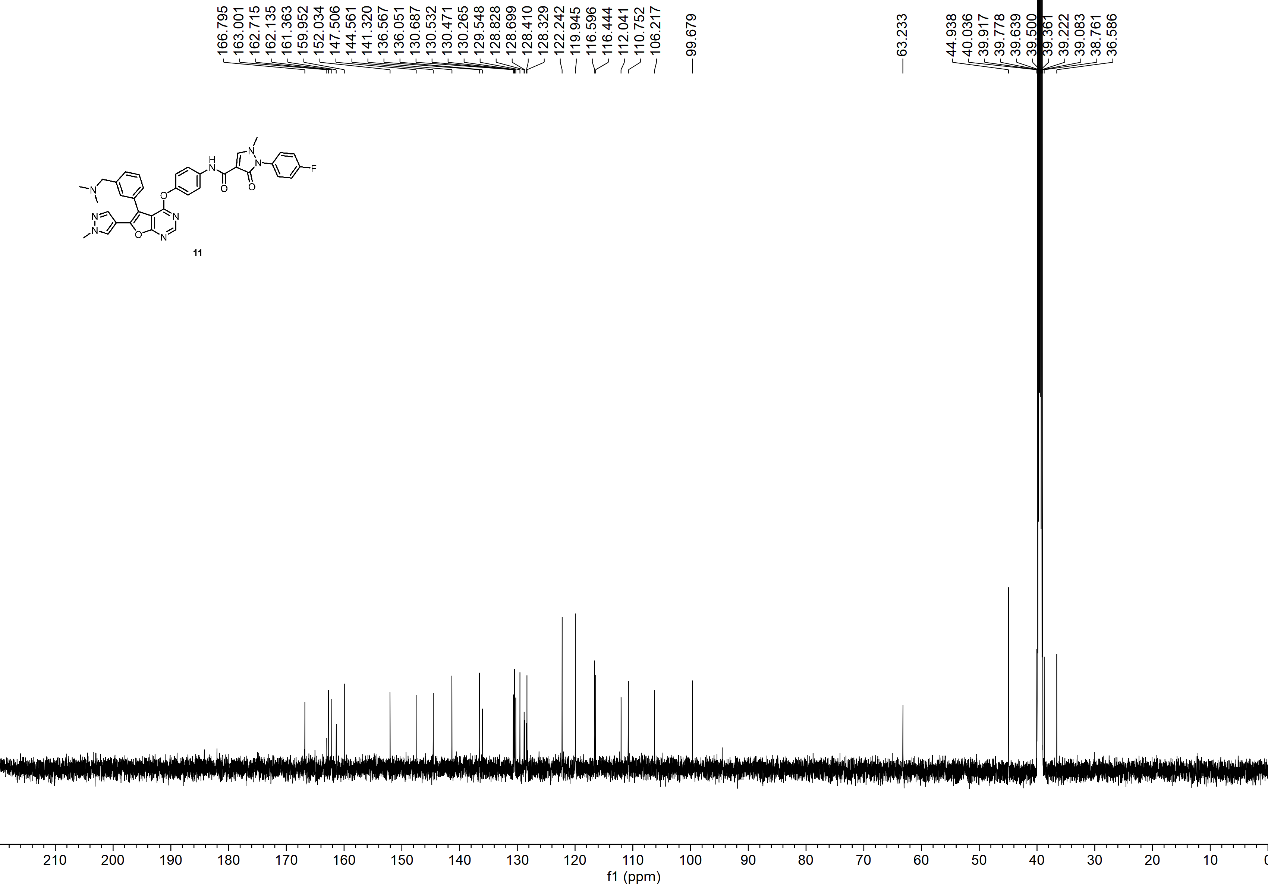


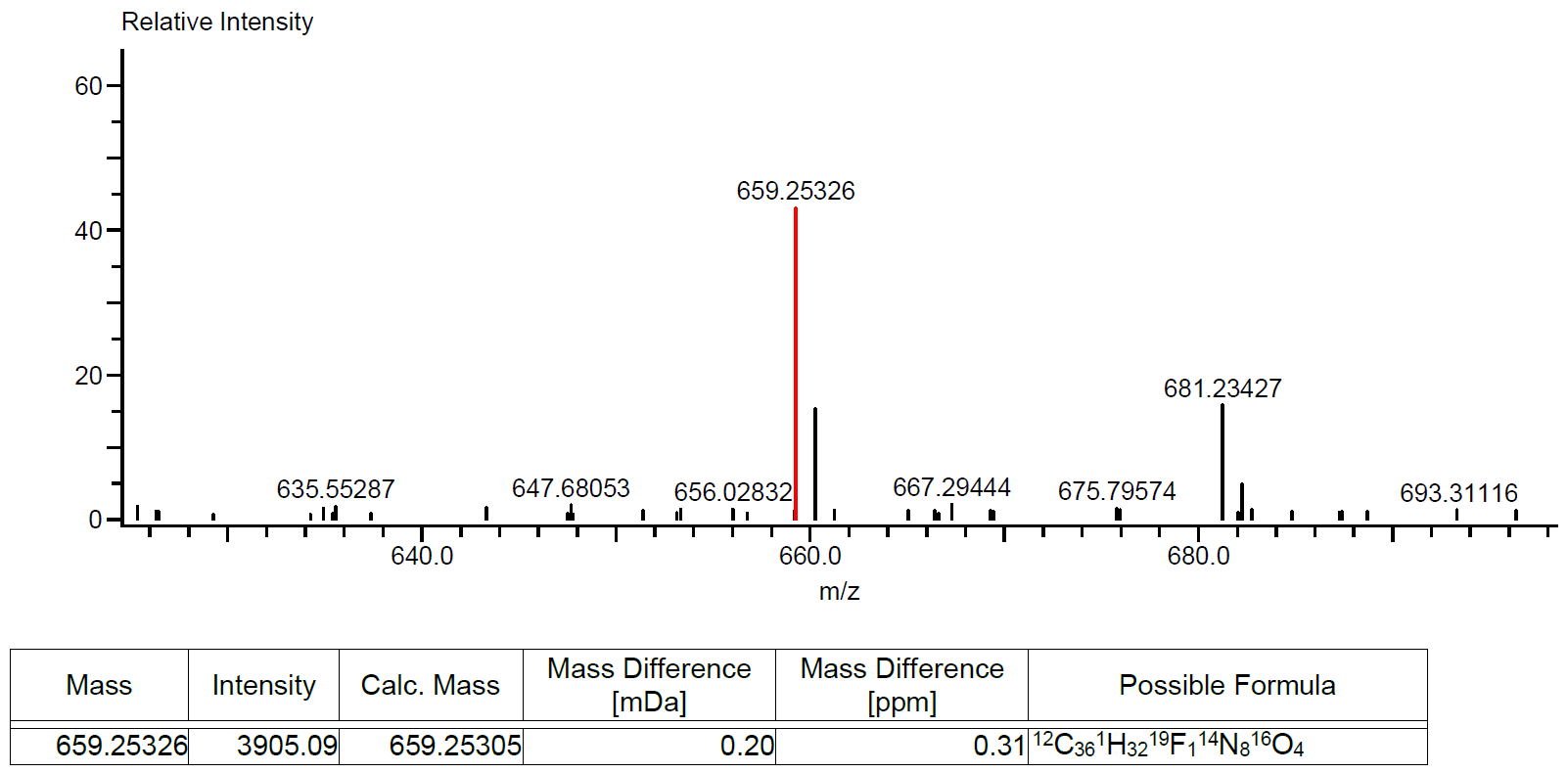


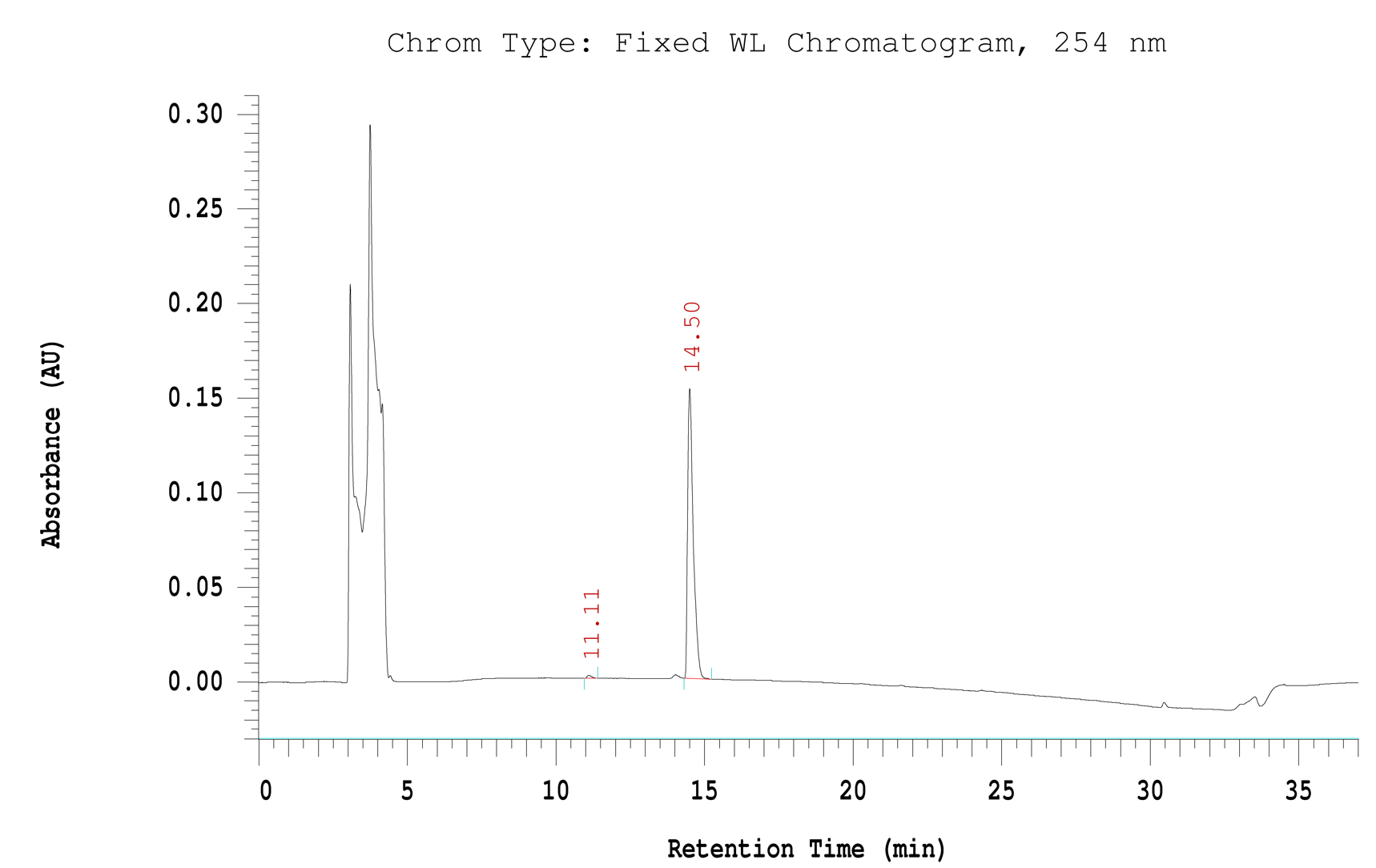

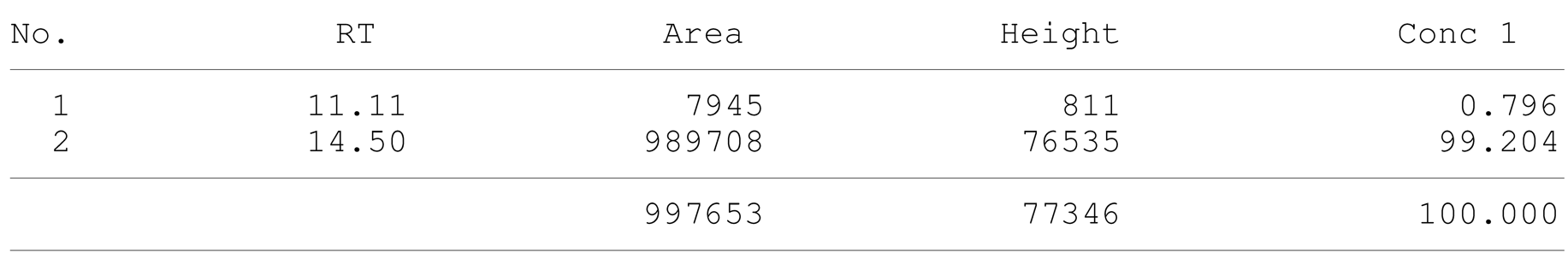

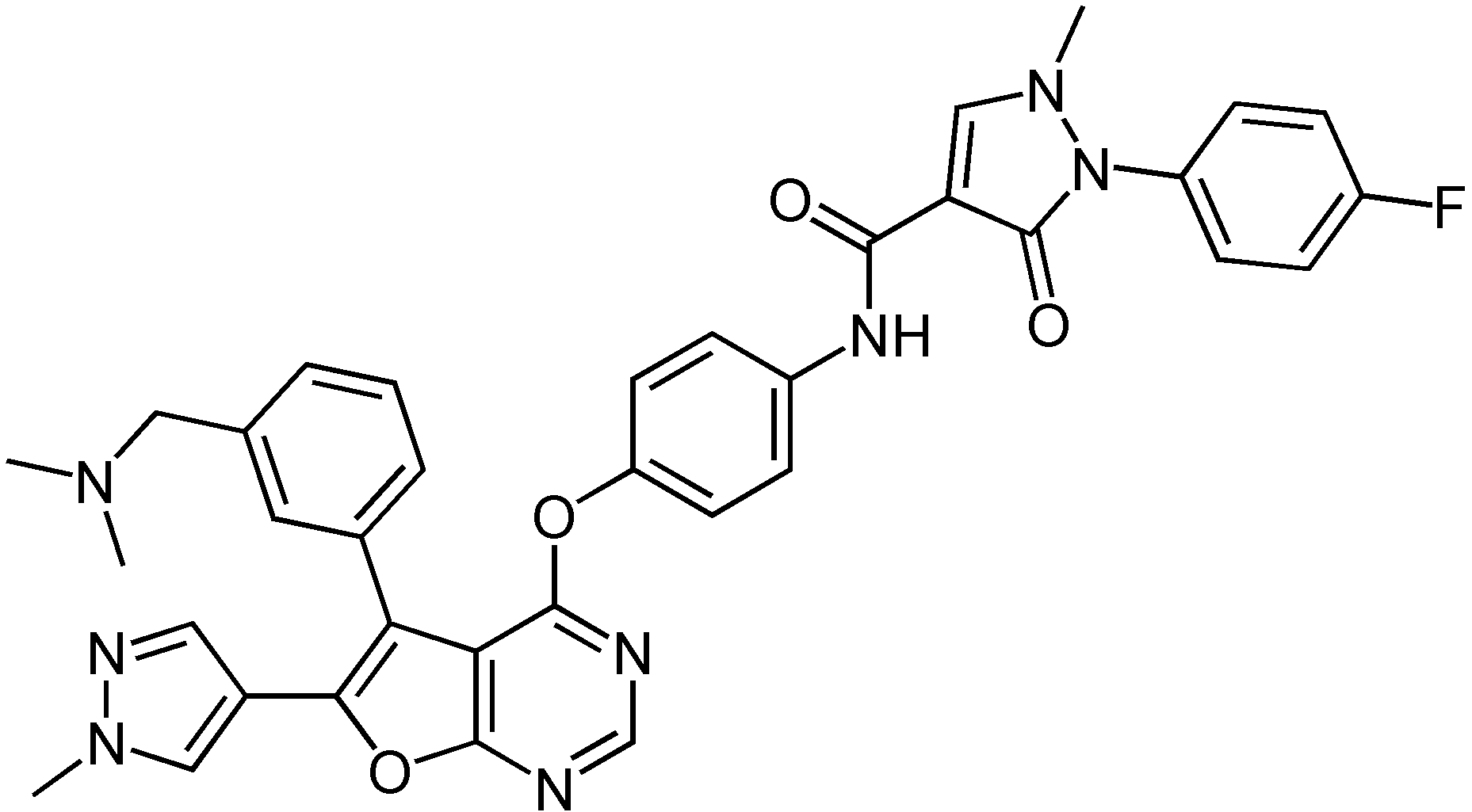


Figure S-9. ^1^H and ^13^C NMR spectra in DMSO-*d*_6_, HPLC trace, and HRMS data of compound **11**_._


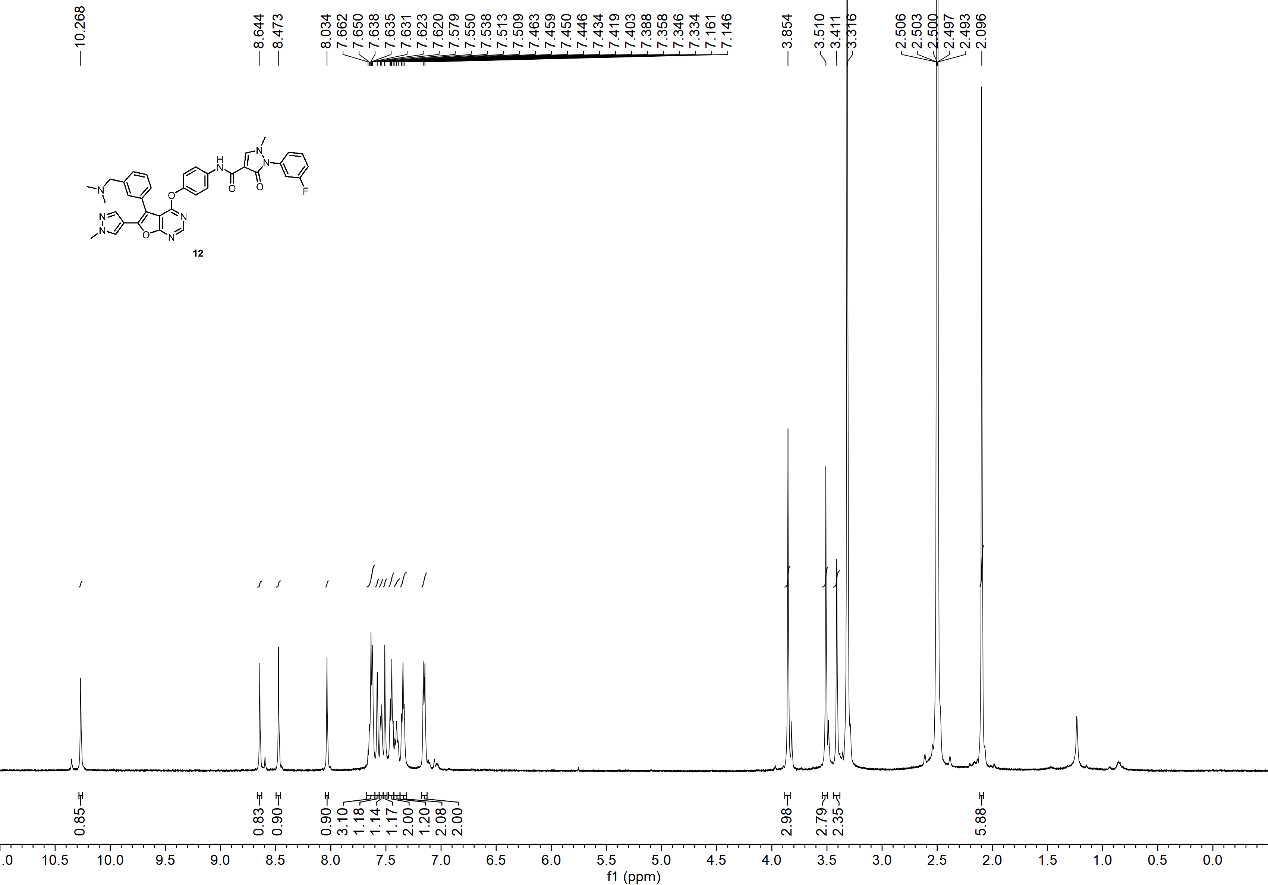


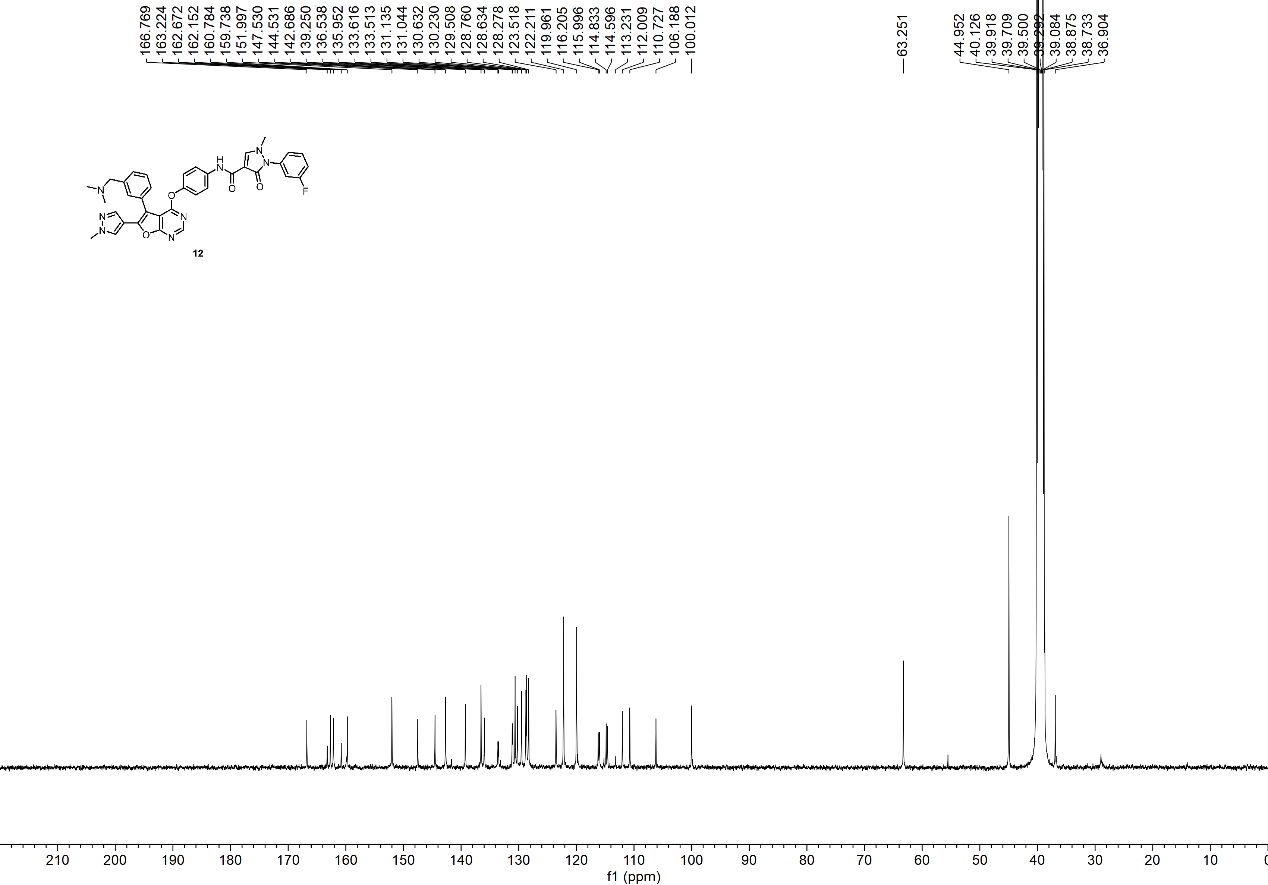


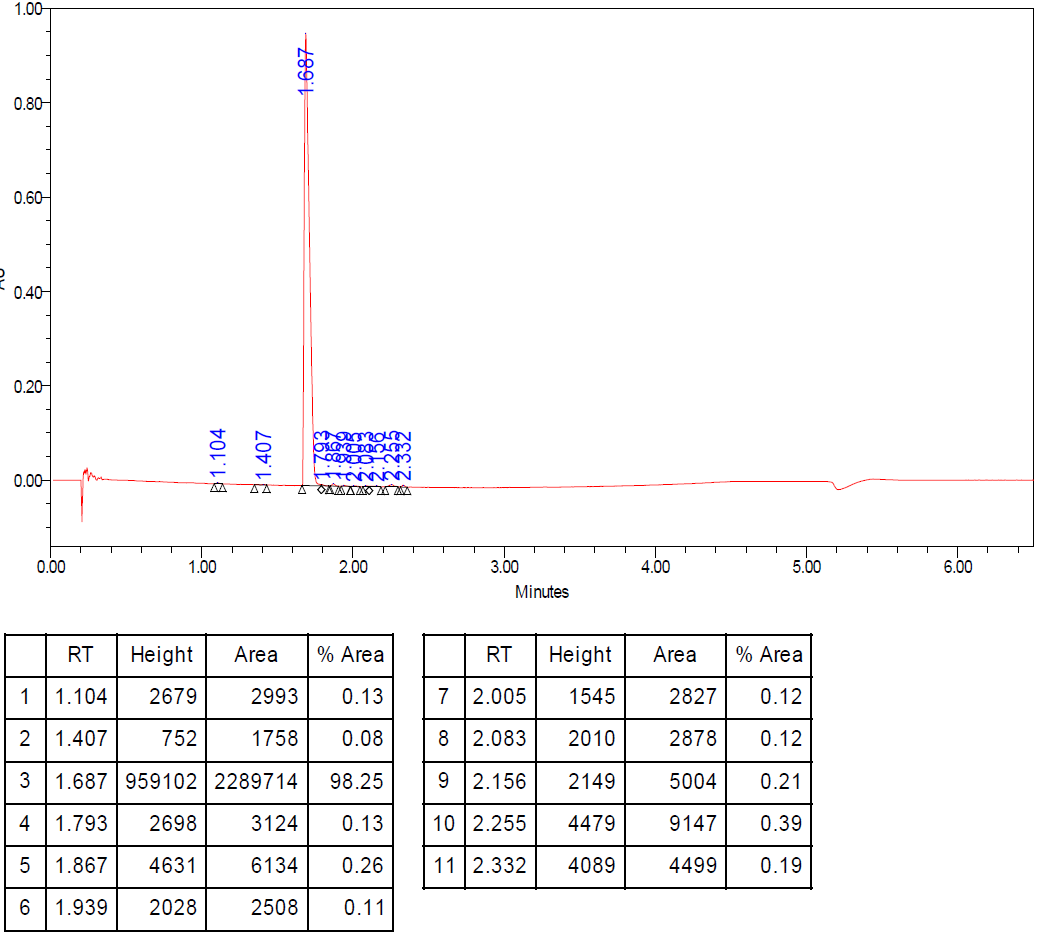


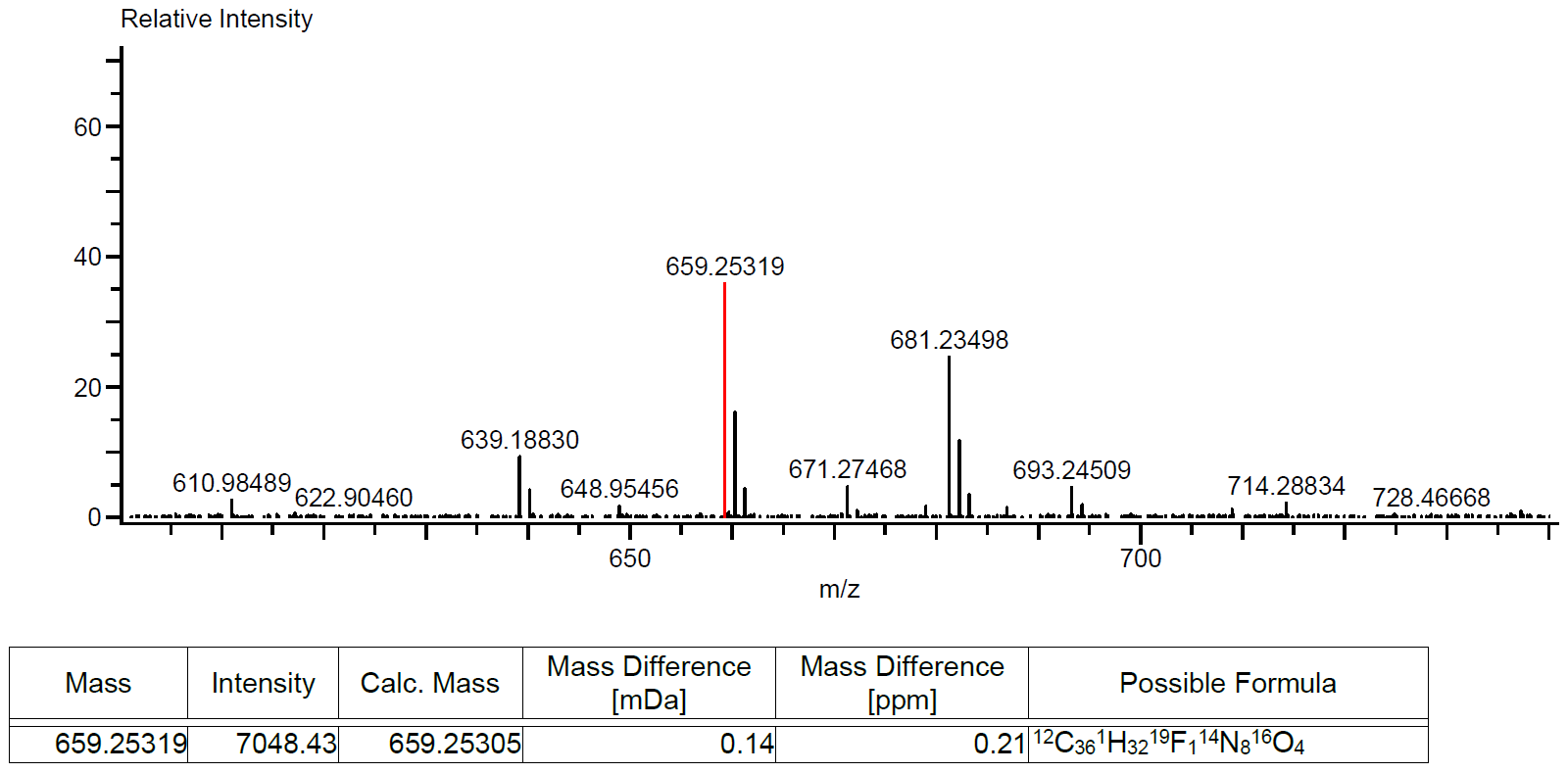


Figure S-10. ^1^H and ^13^C NMR spectra in DMSO-*d*_6_, HPLC trace, and HRMS data of compound **12**_._


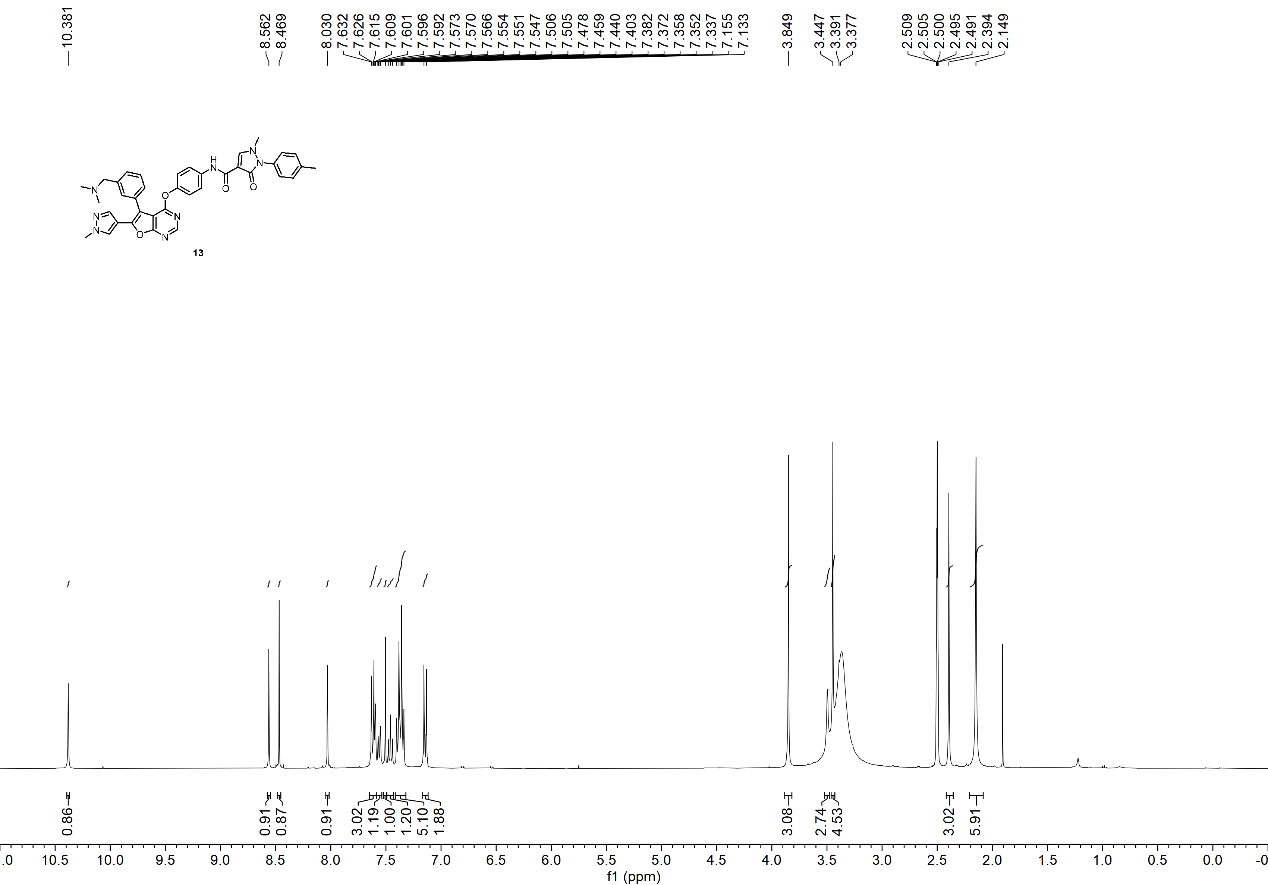


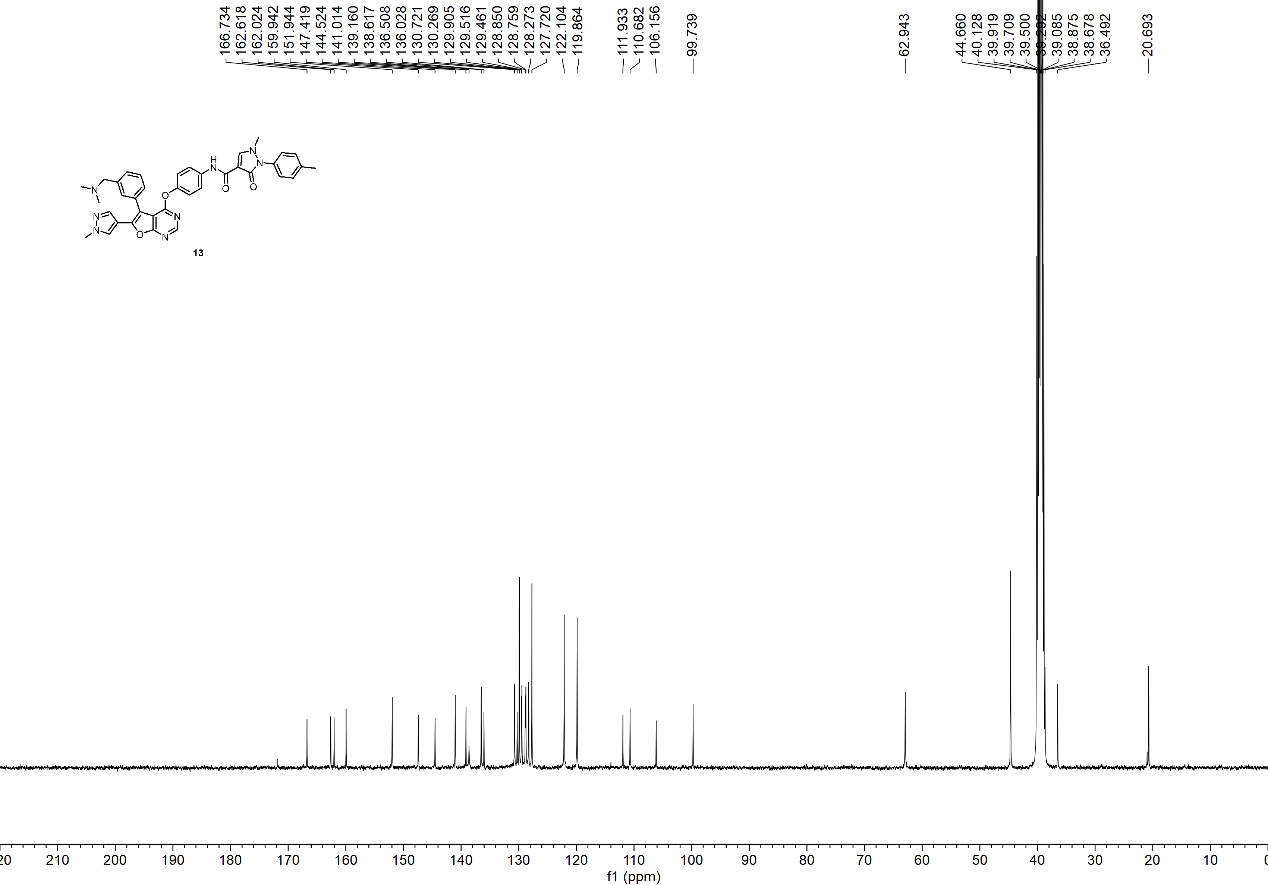


**
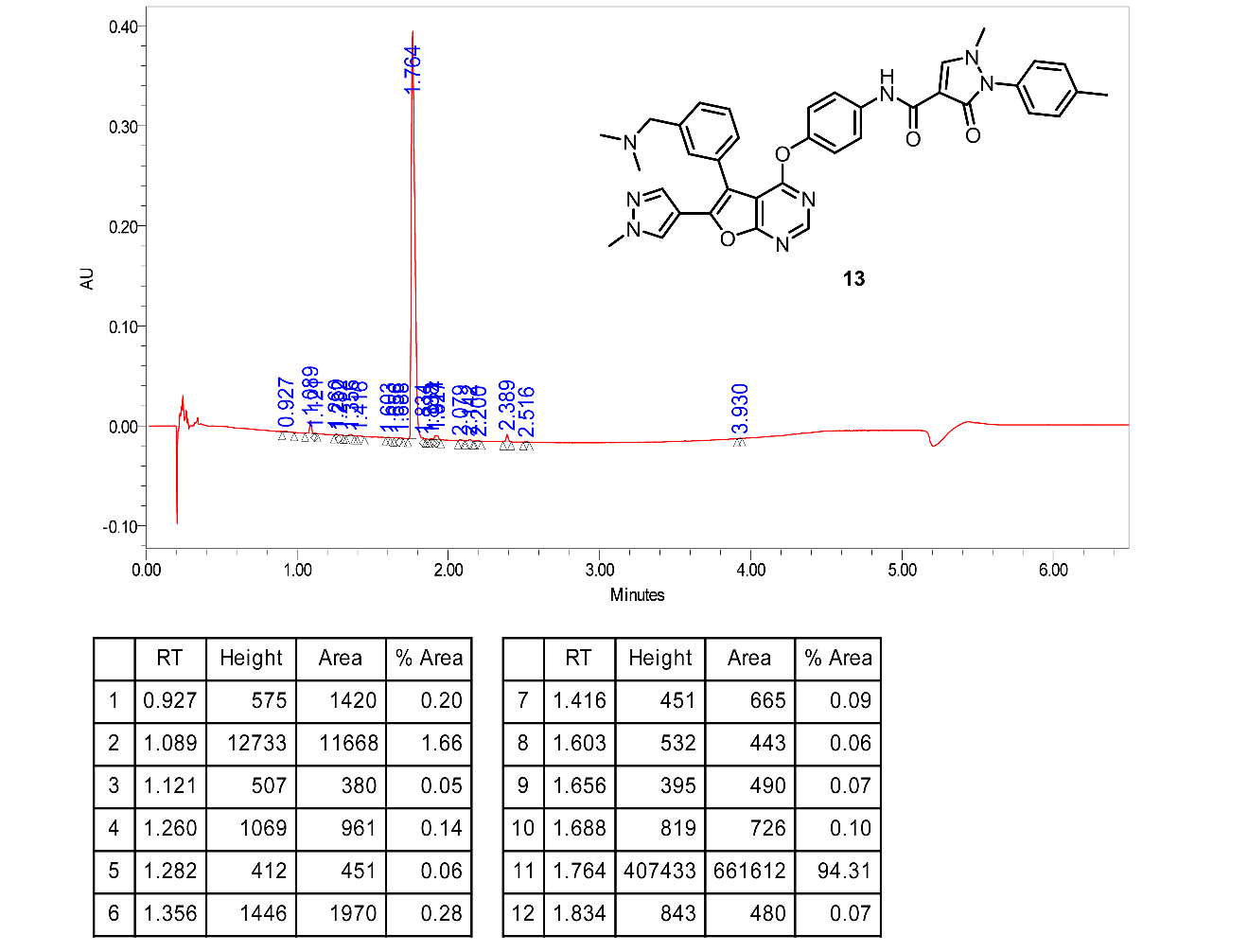
**


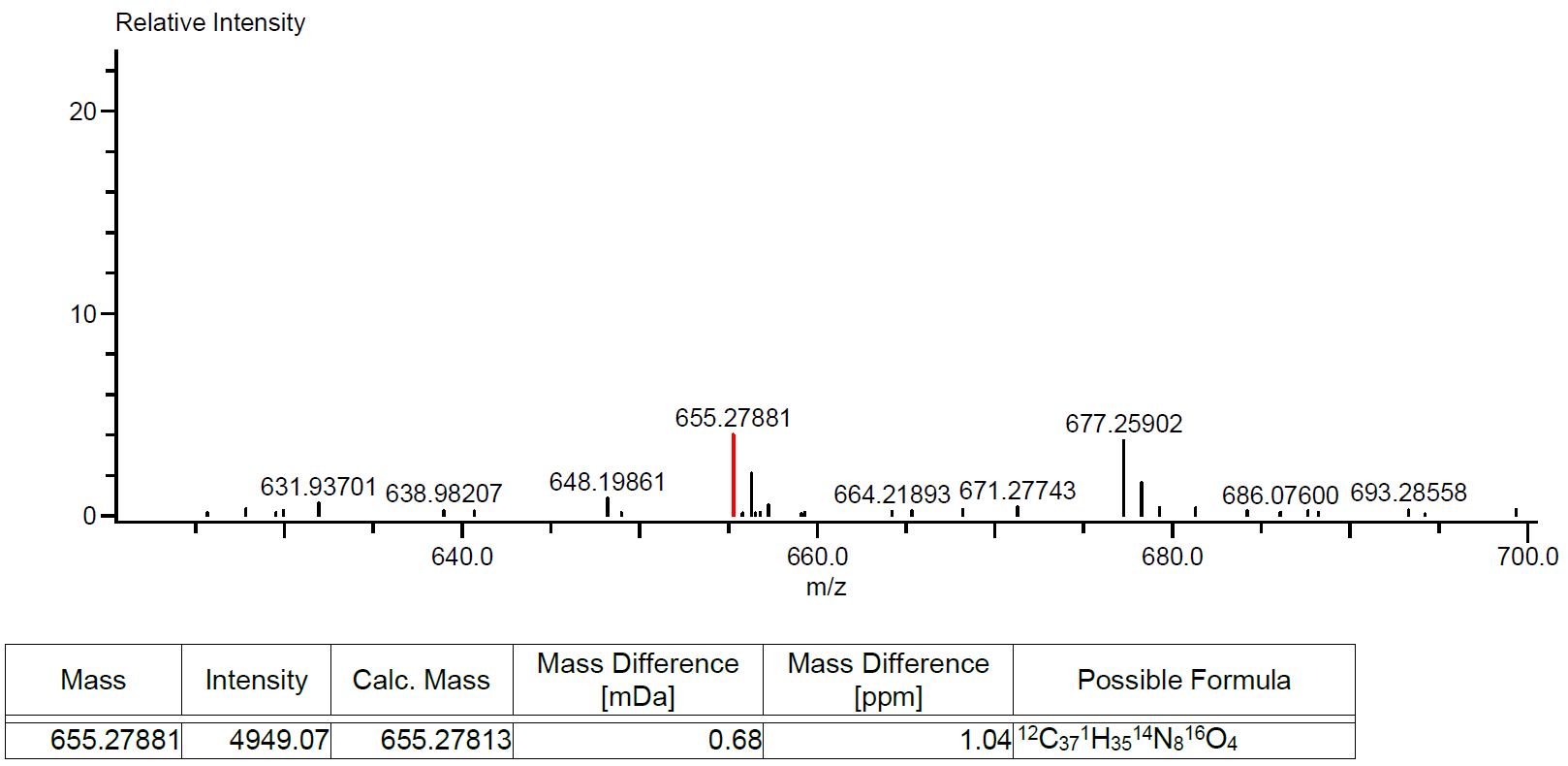


Figure S-11. ^1^H and ^13^C NMR spectra in DMSO-*d*_6_, HPLC trace, and HRMS data of compound **13**_._


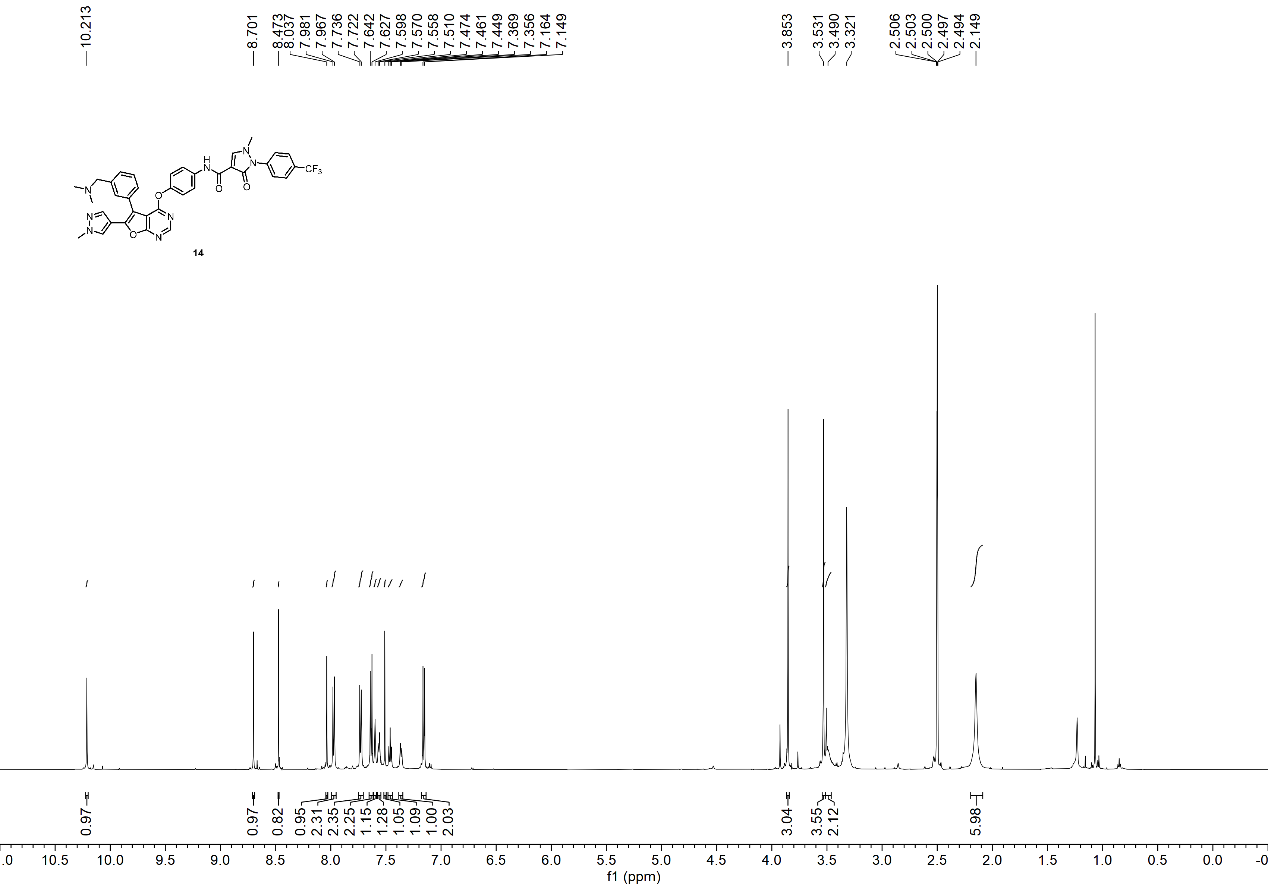


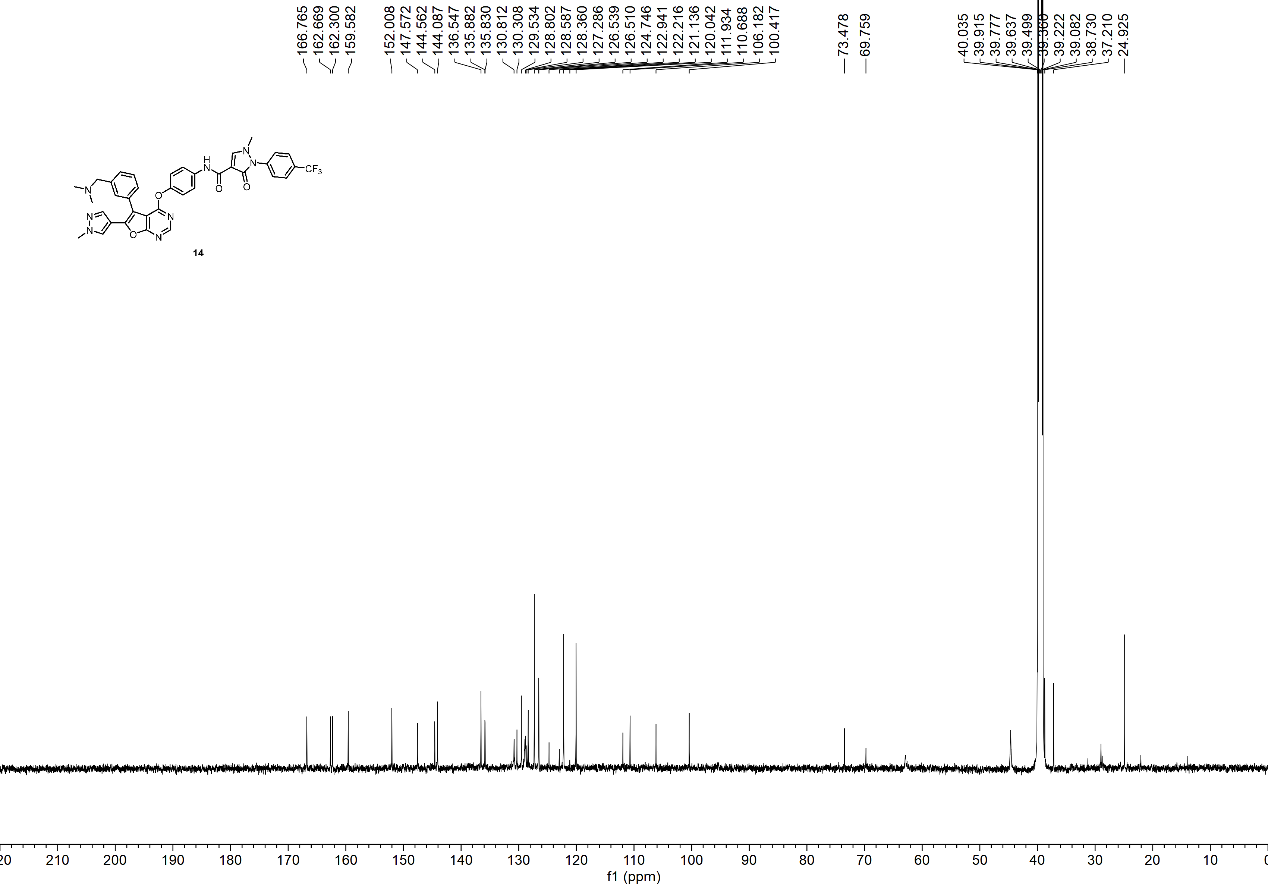


**
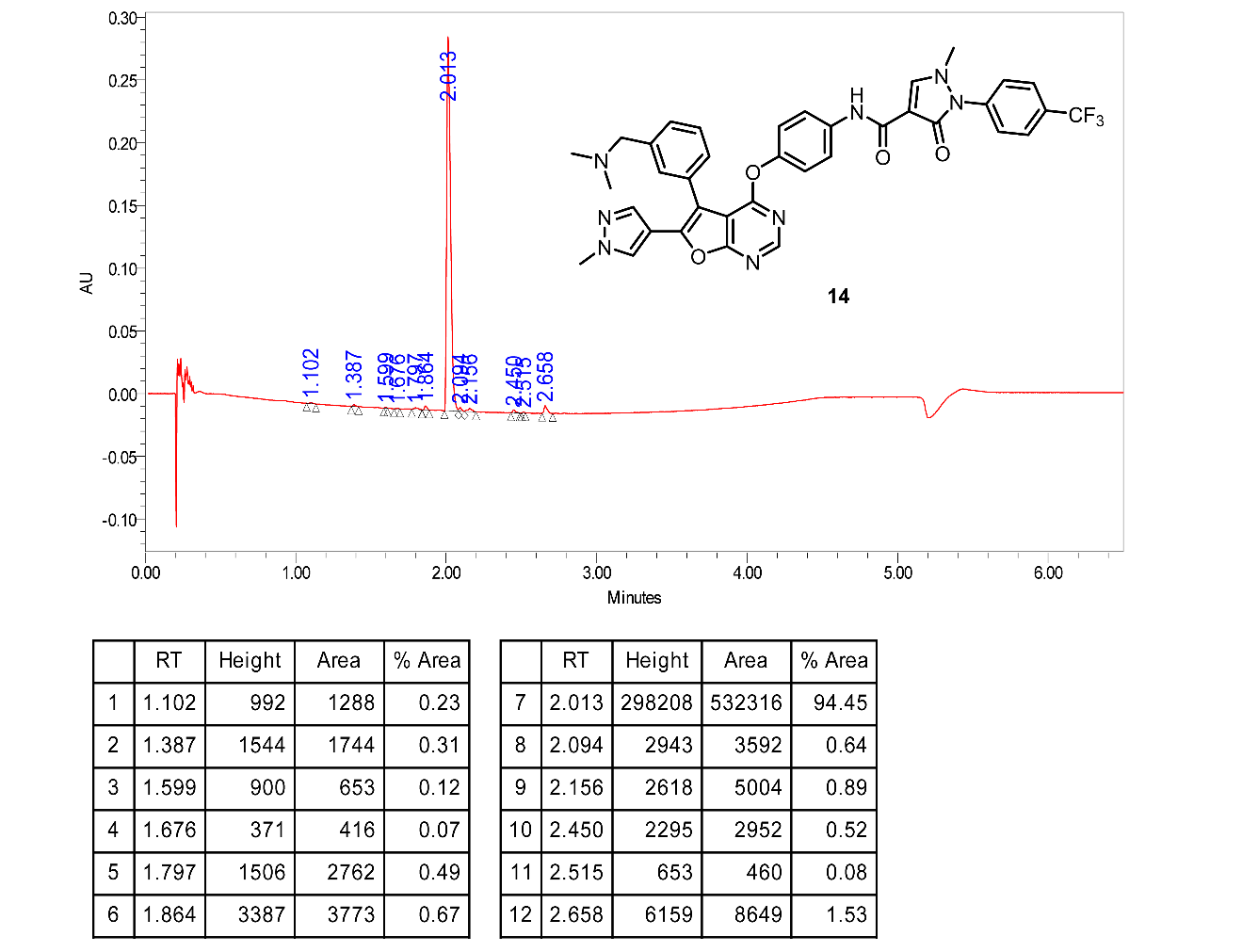
**


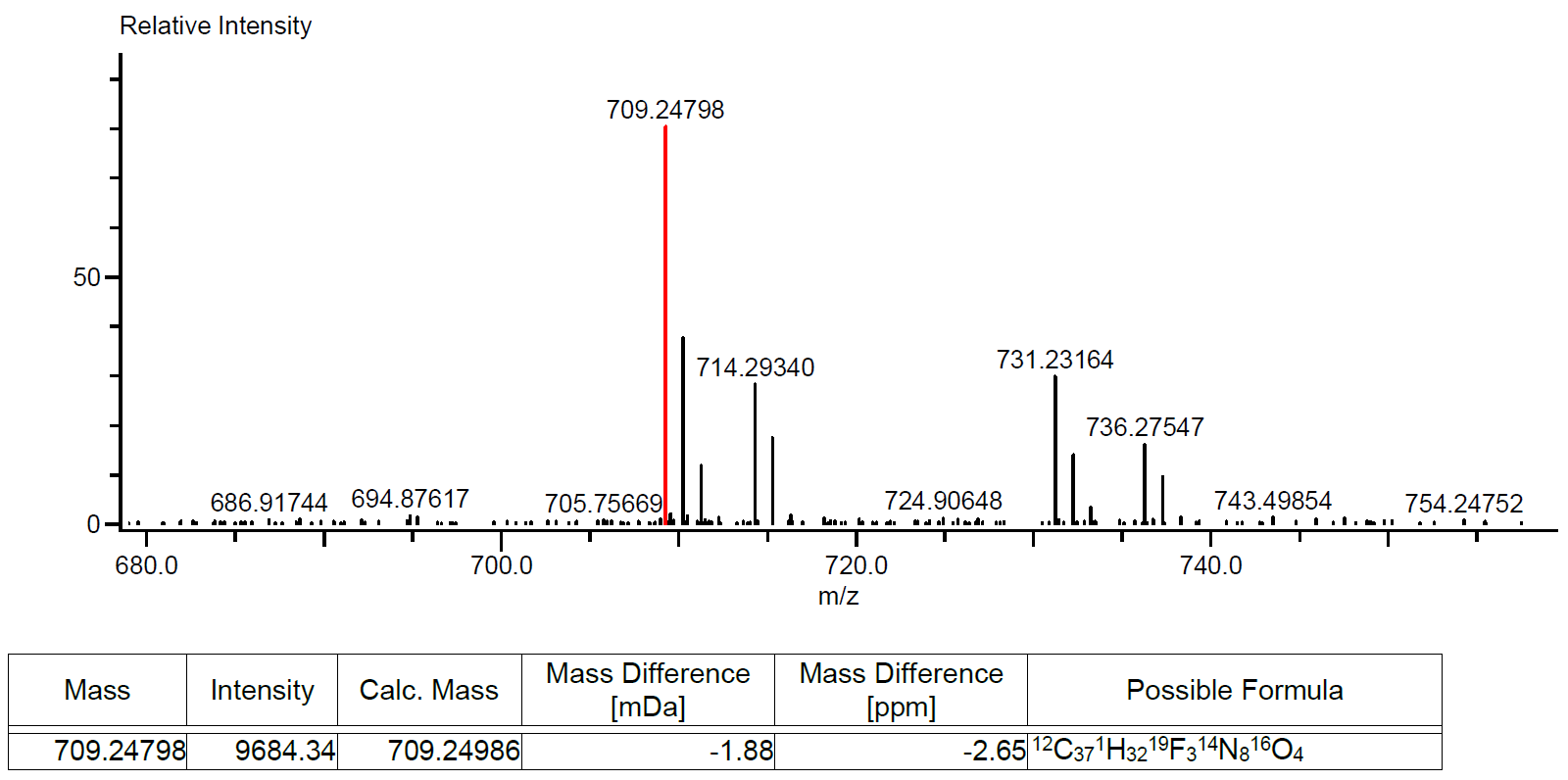


Figure S-12. ^1^H and ^13^C NMR spectra in DMSO-*d*_6_, HPLC trace, and HRMS data of compound **14**_._


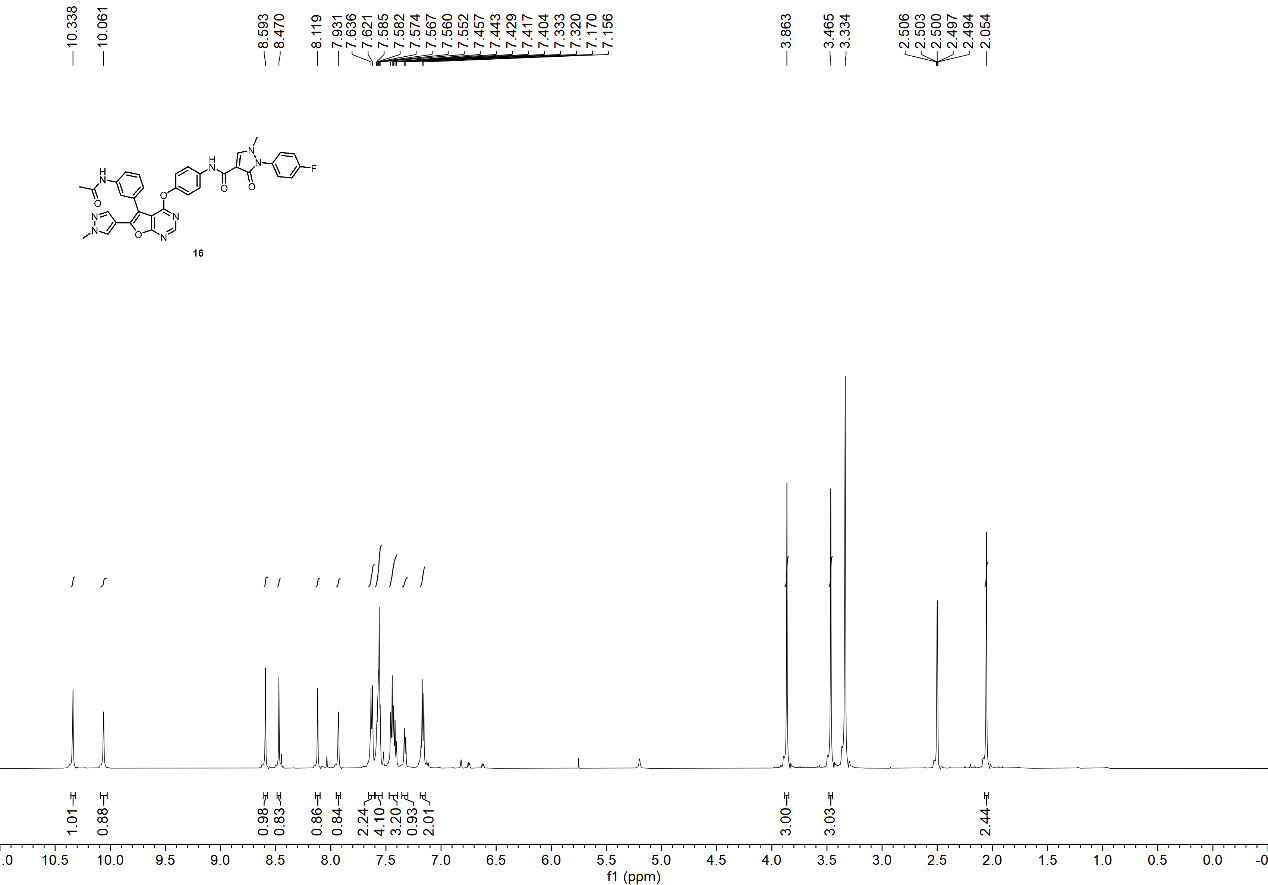


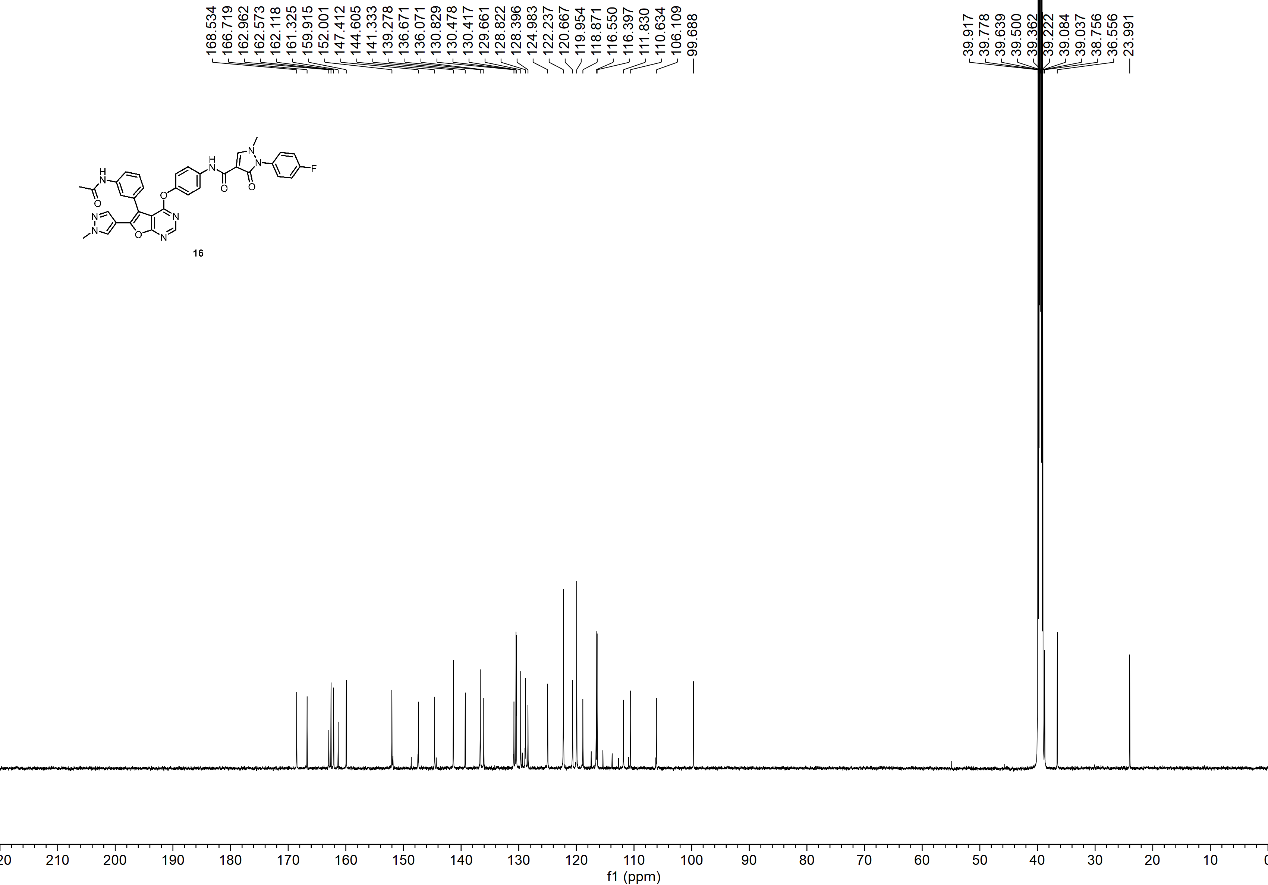


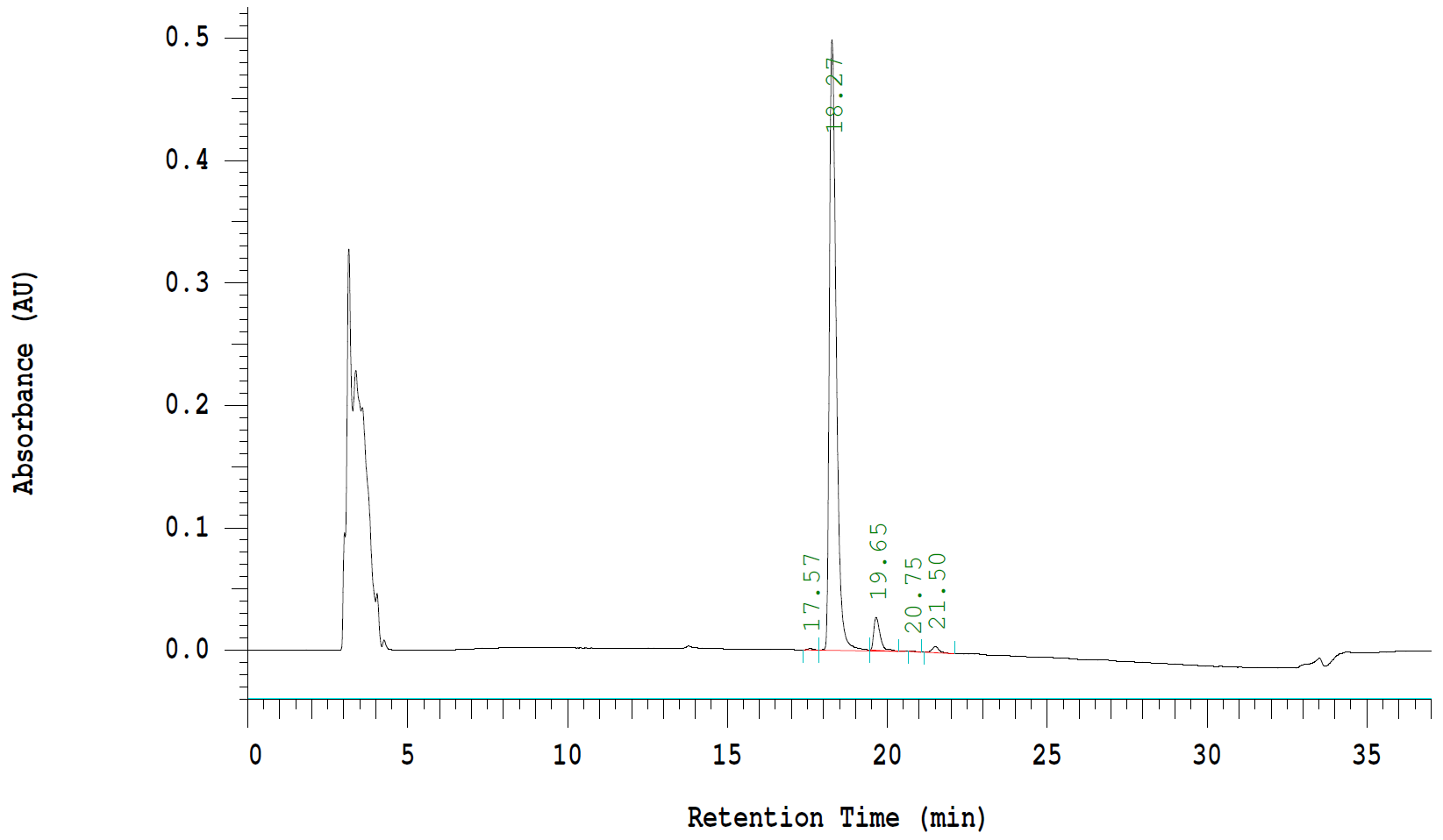


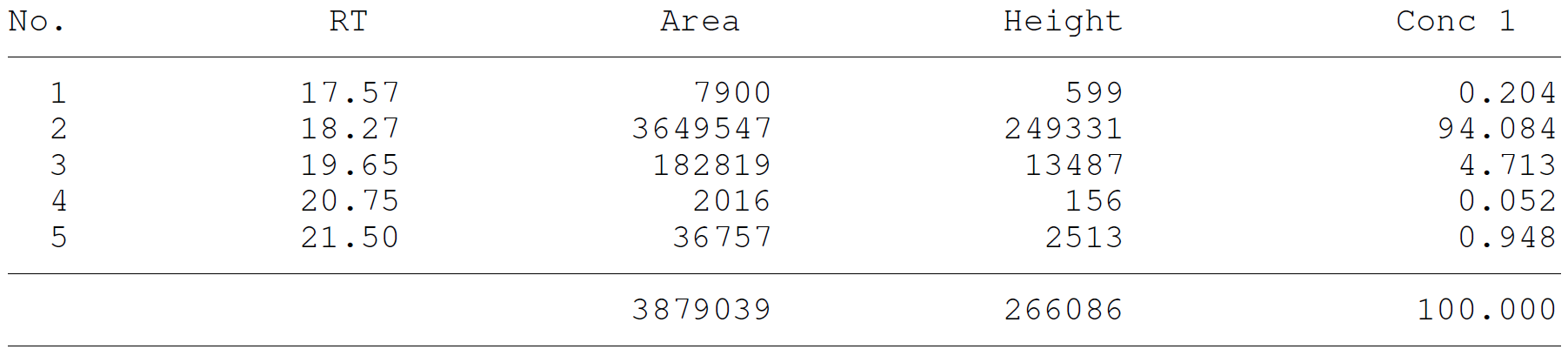


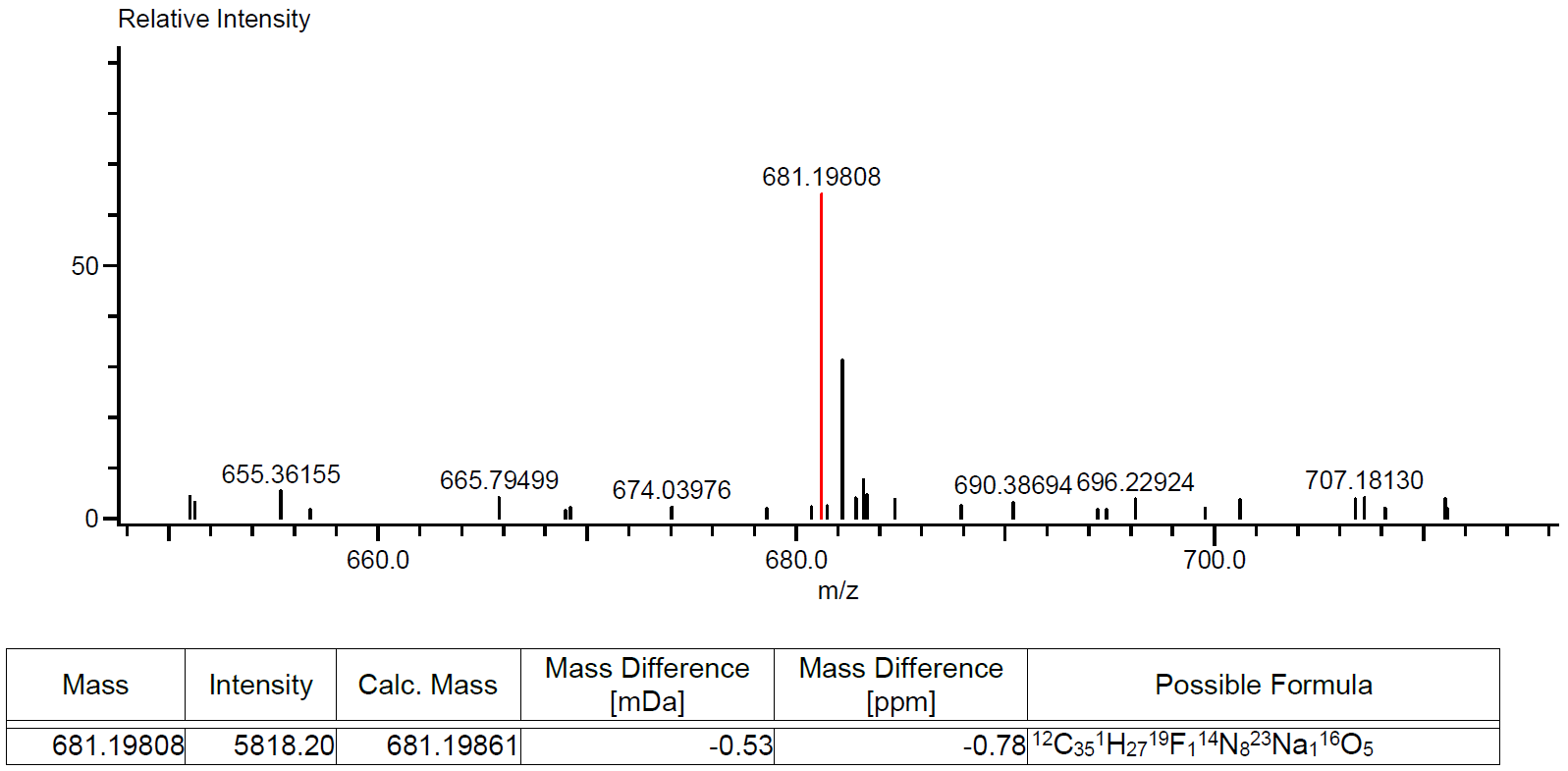


Figure S-13. ^1^H and ^13^C NMR spectra in DMSO-*d*_6_, HPLC trace, and HRMS data of compound **16**_._


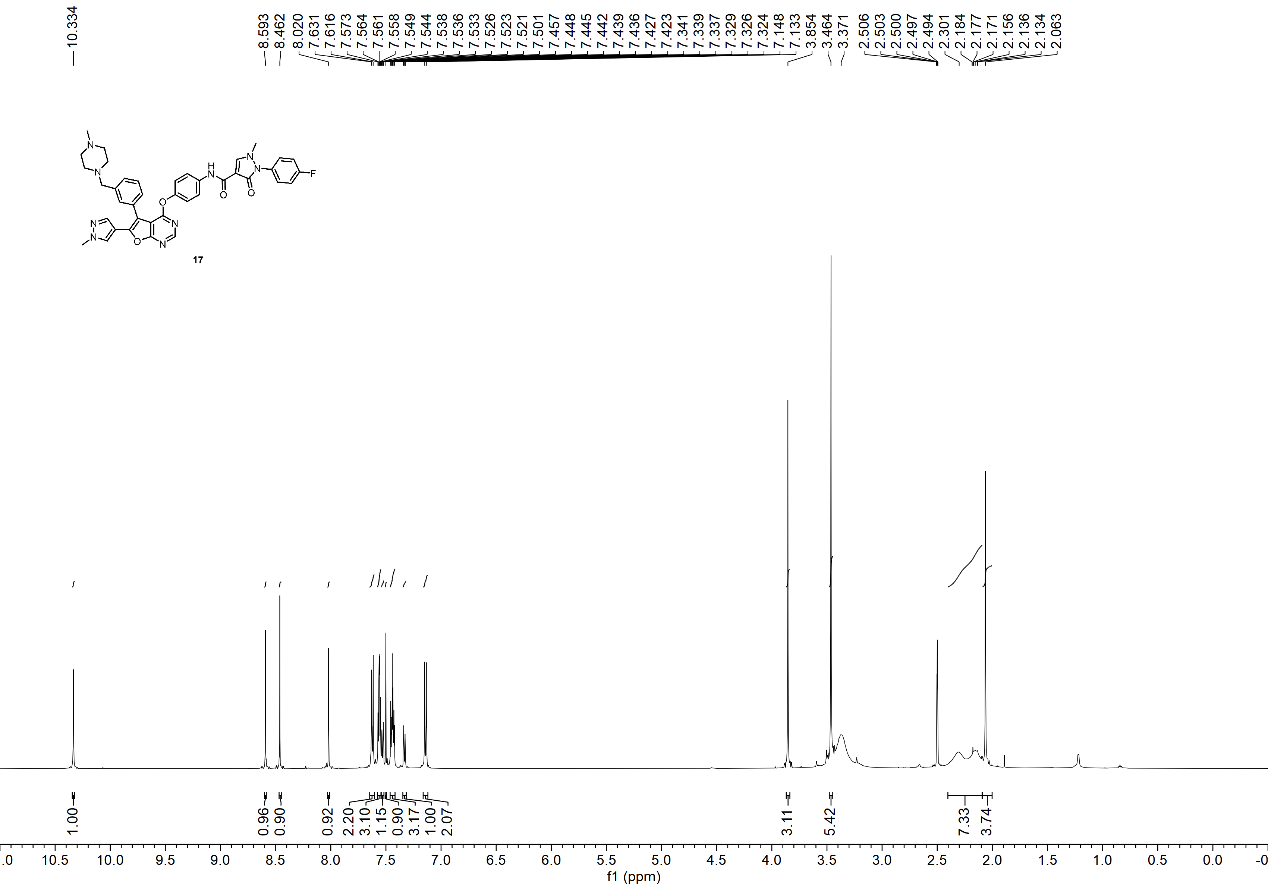


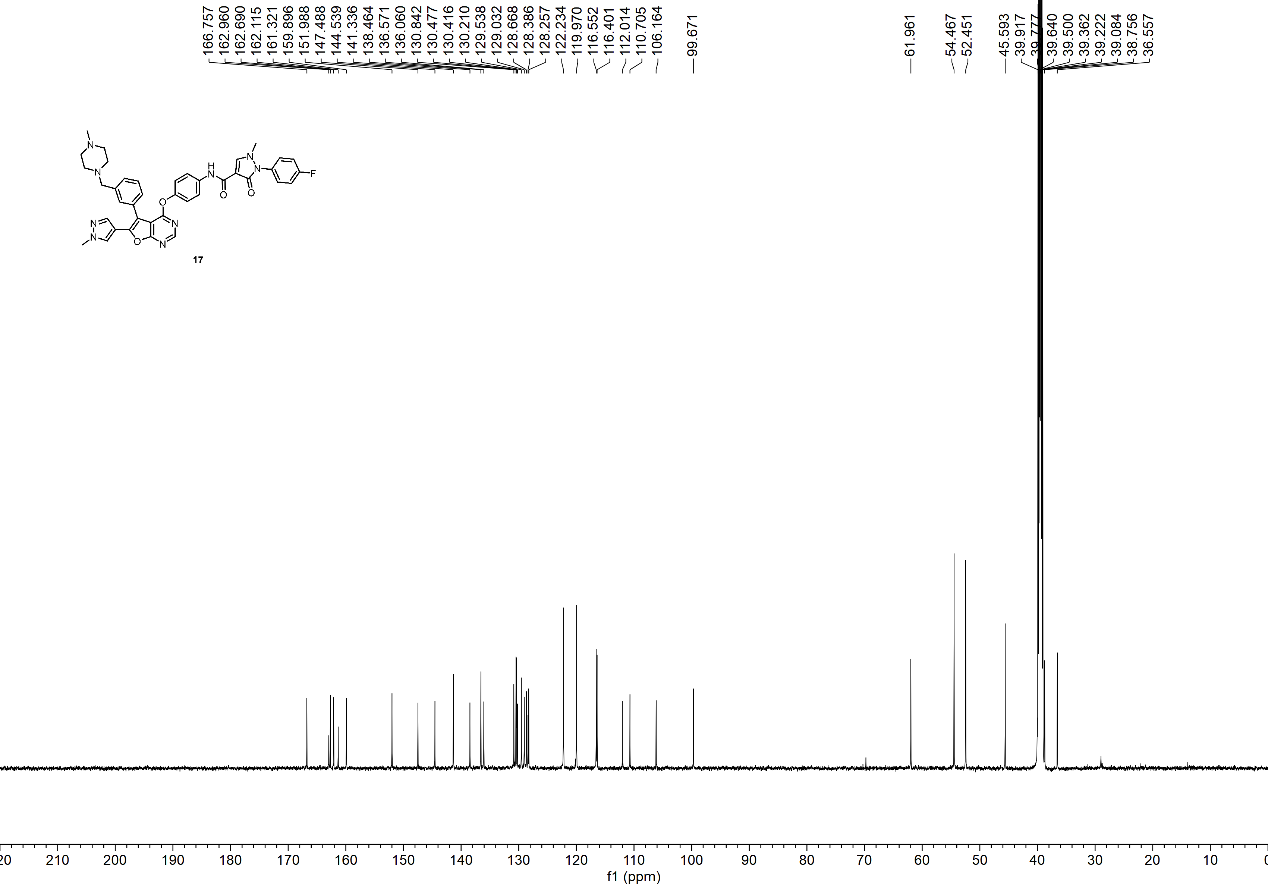


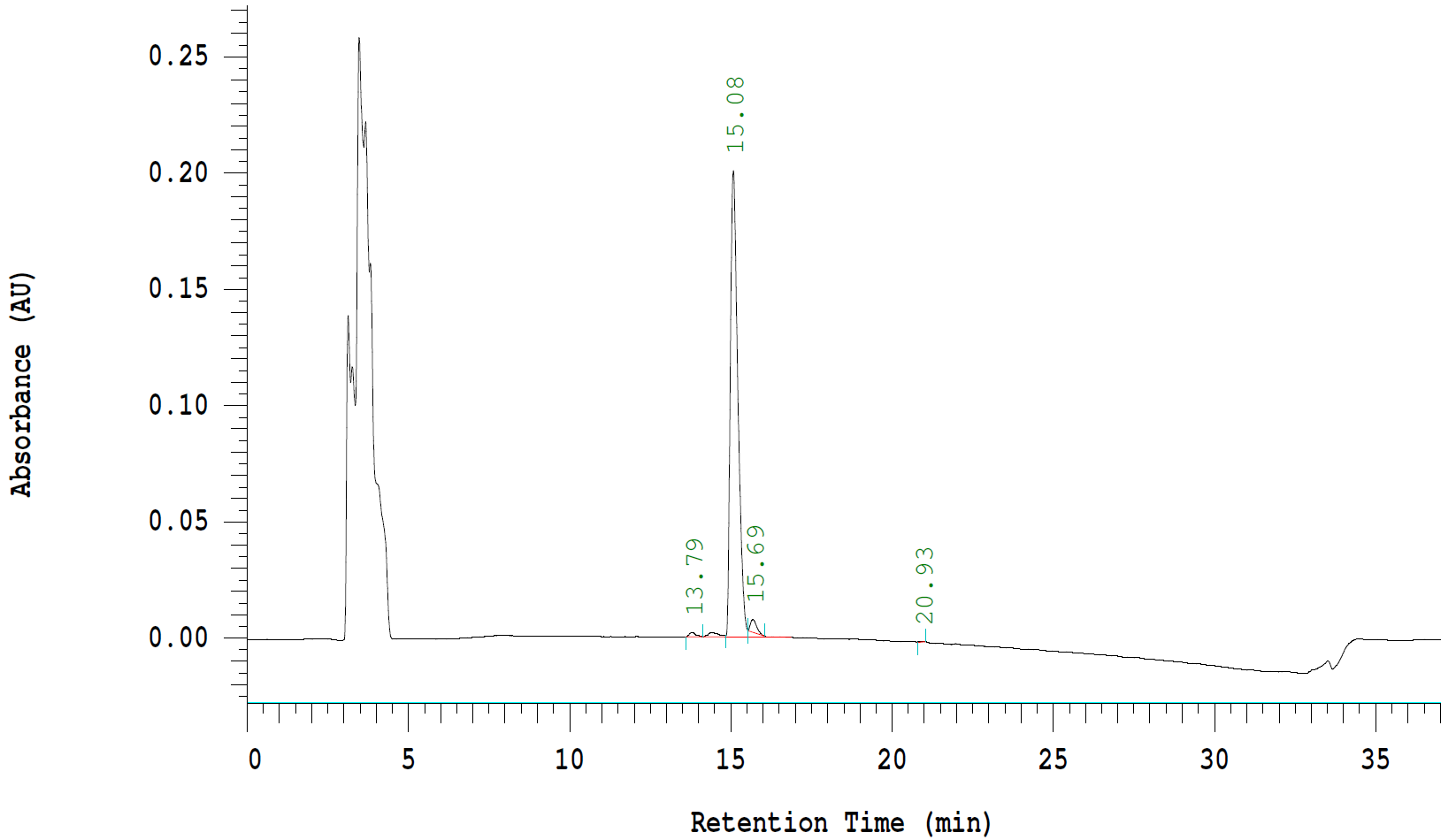


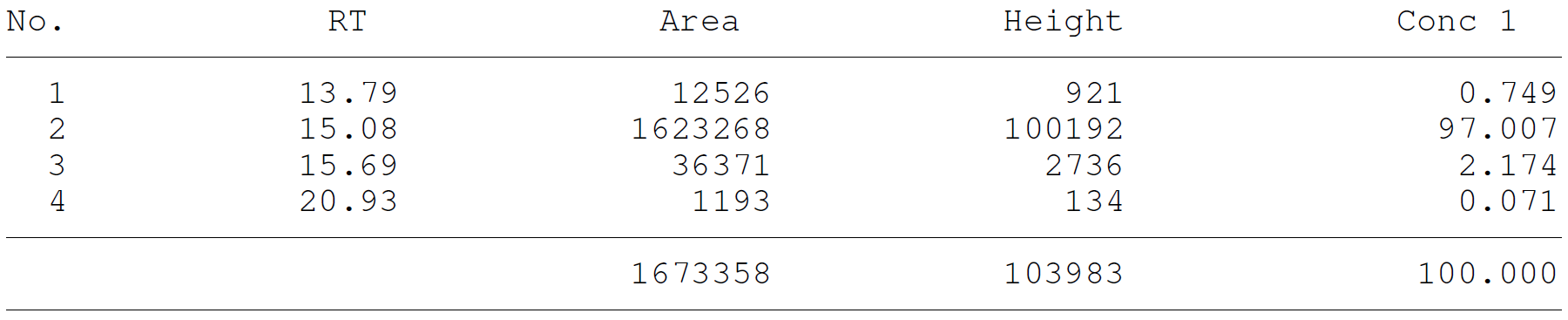


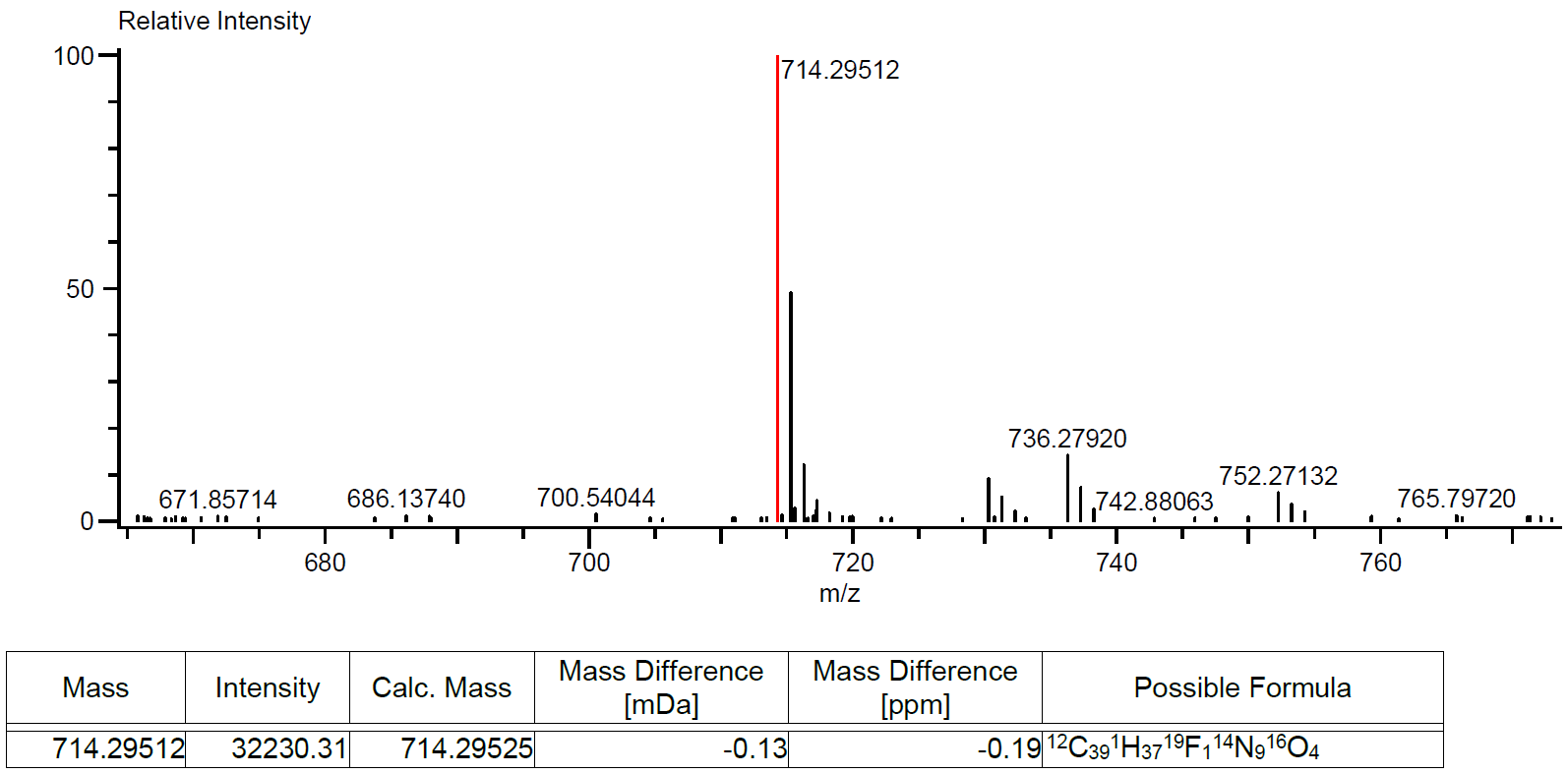


Figure S-14. ^1^H and ^13^C NMR spectra in DMSO-*d*_6_, HPLC trace, and HRMS data of compound **17**_._


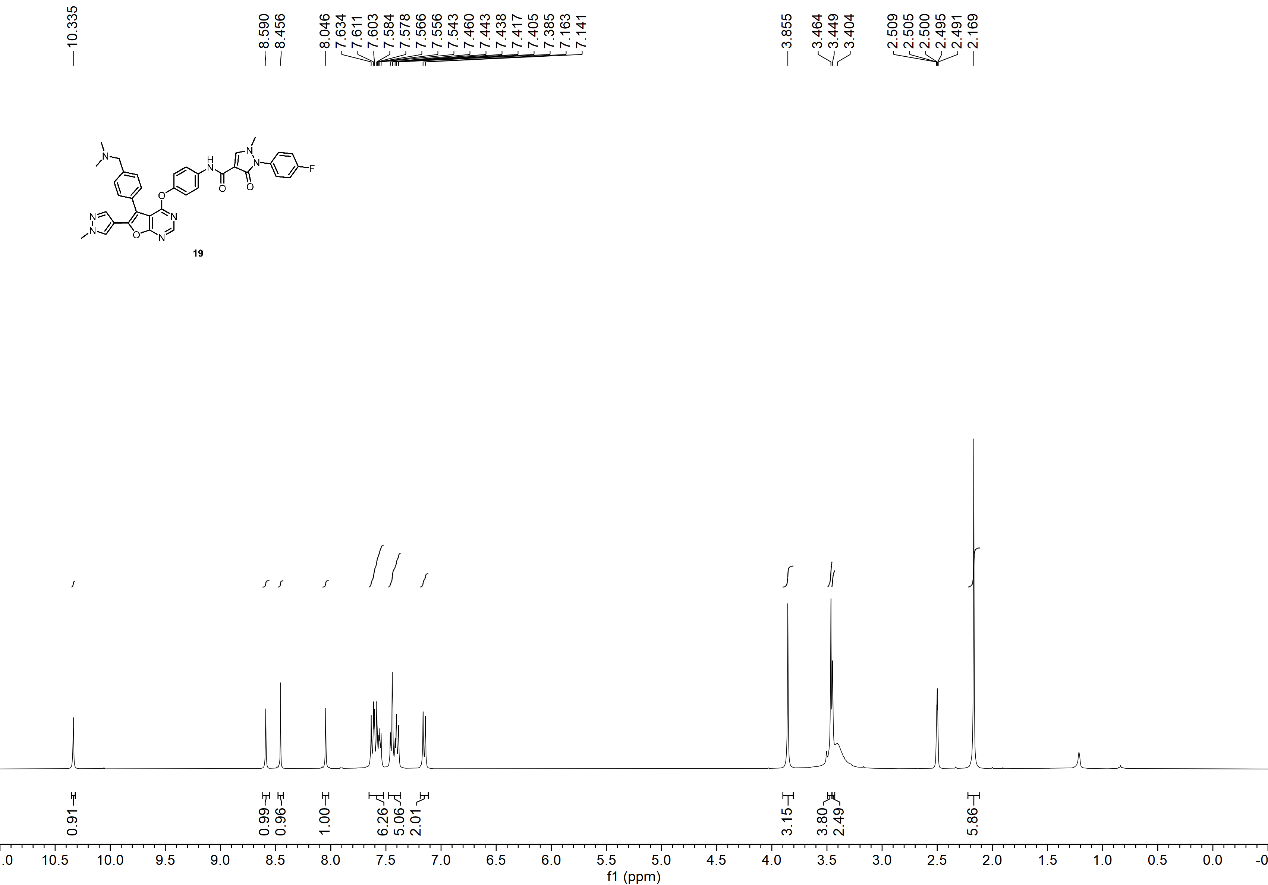

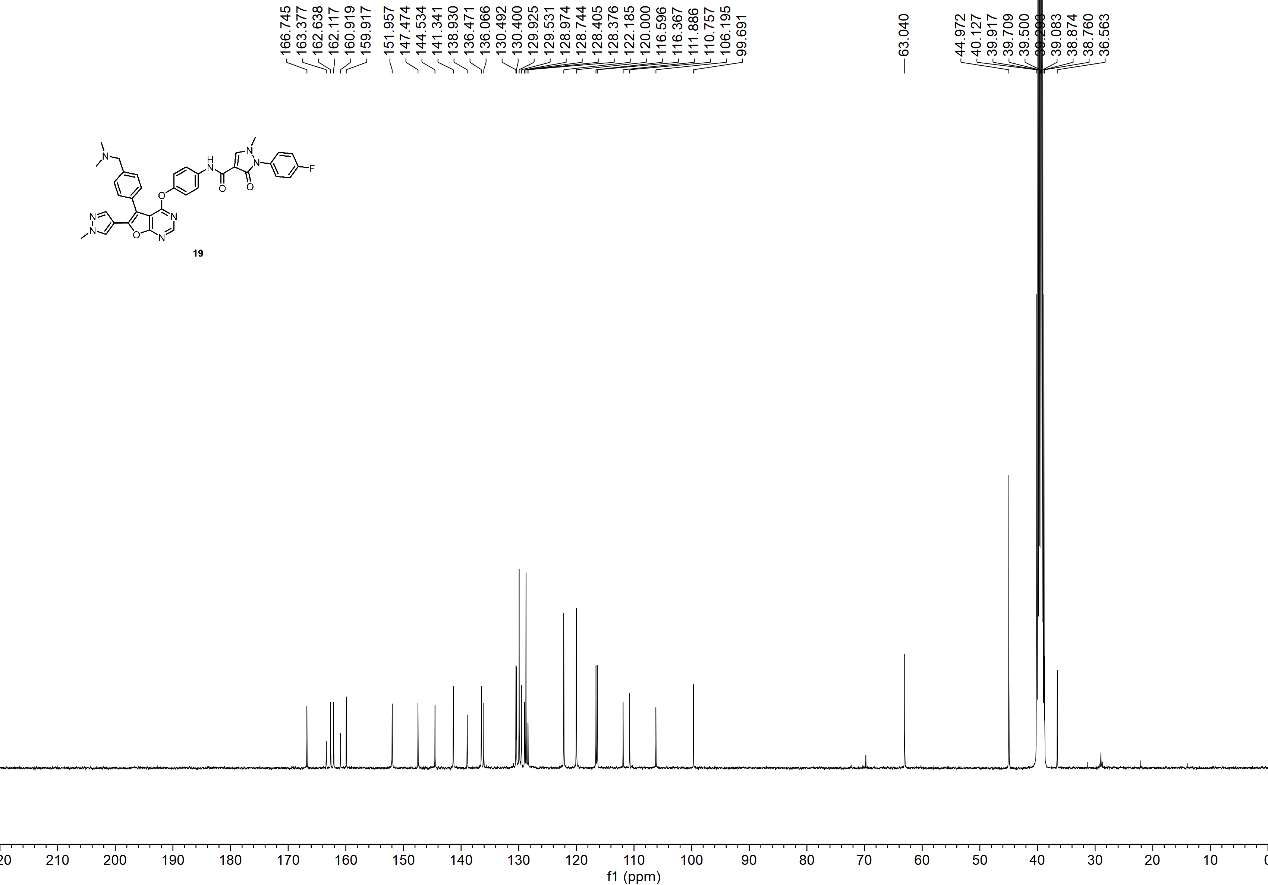


**
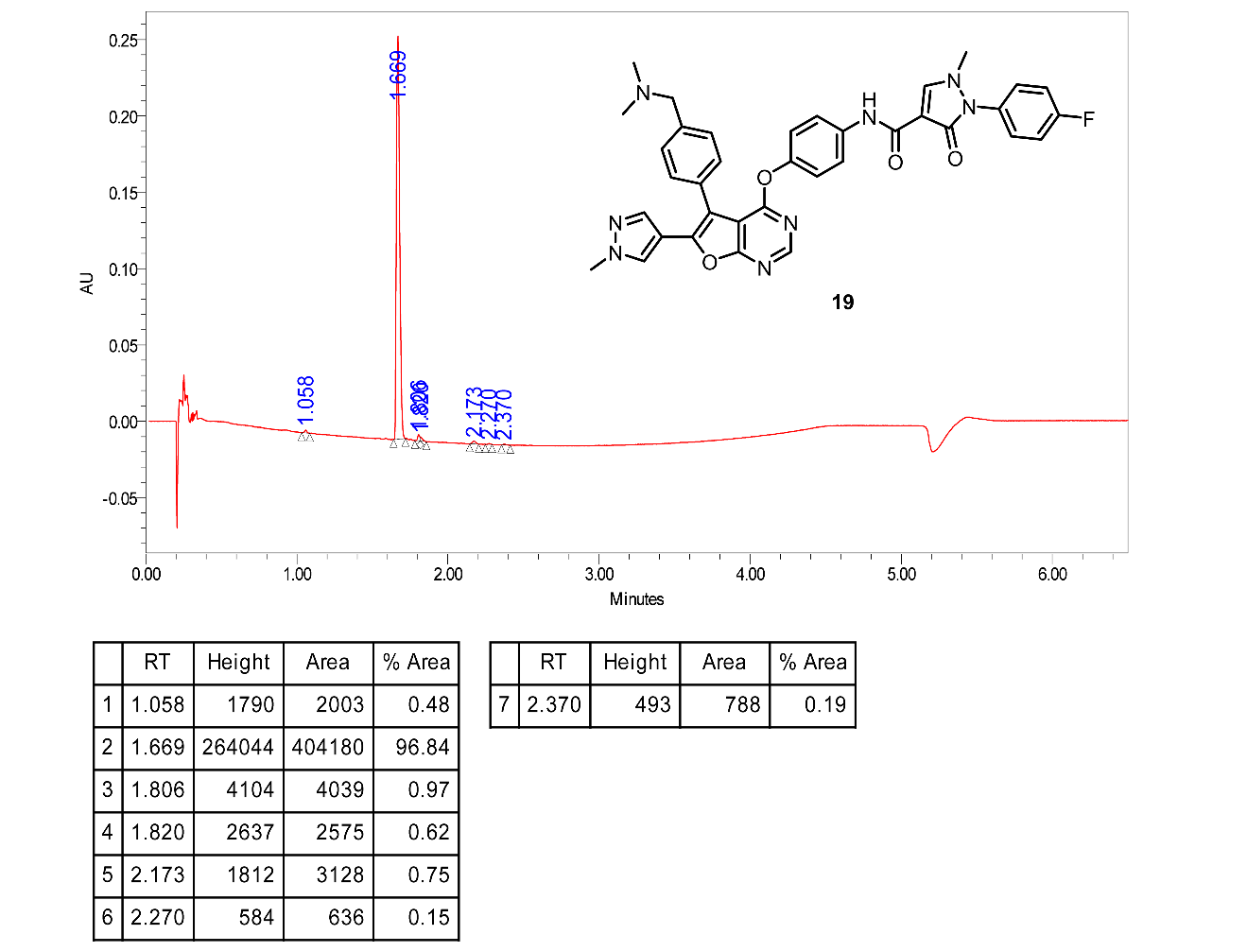
**


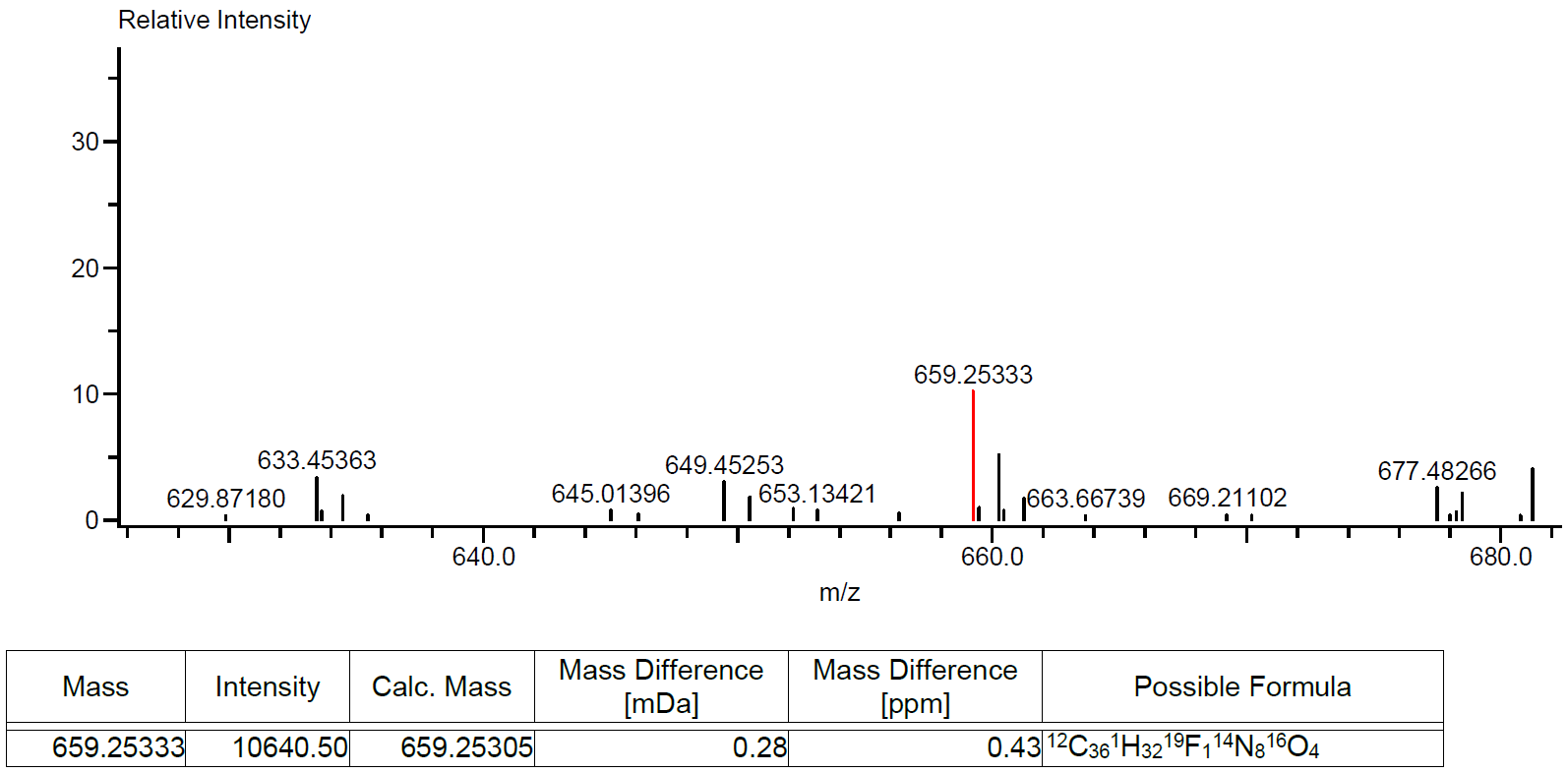


Figure S-15. ^1^H and ^13^C NMR spectra in DMSO-*d*_6_, HPLC trace, and HRMS data of compound **19**_._


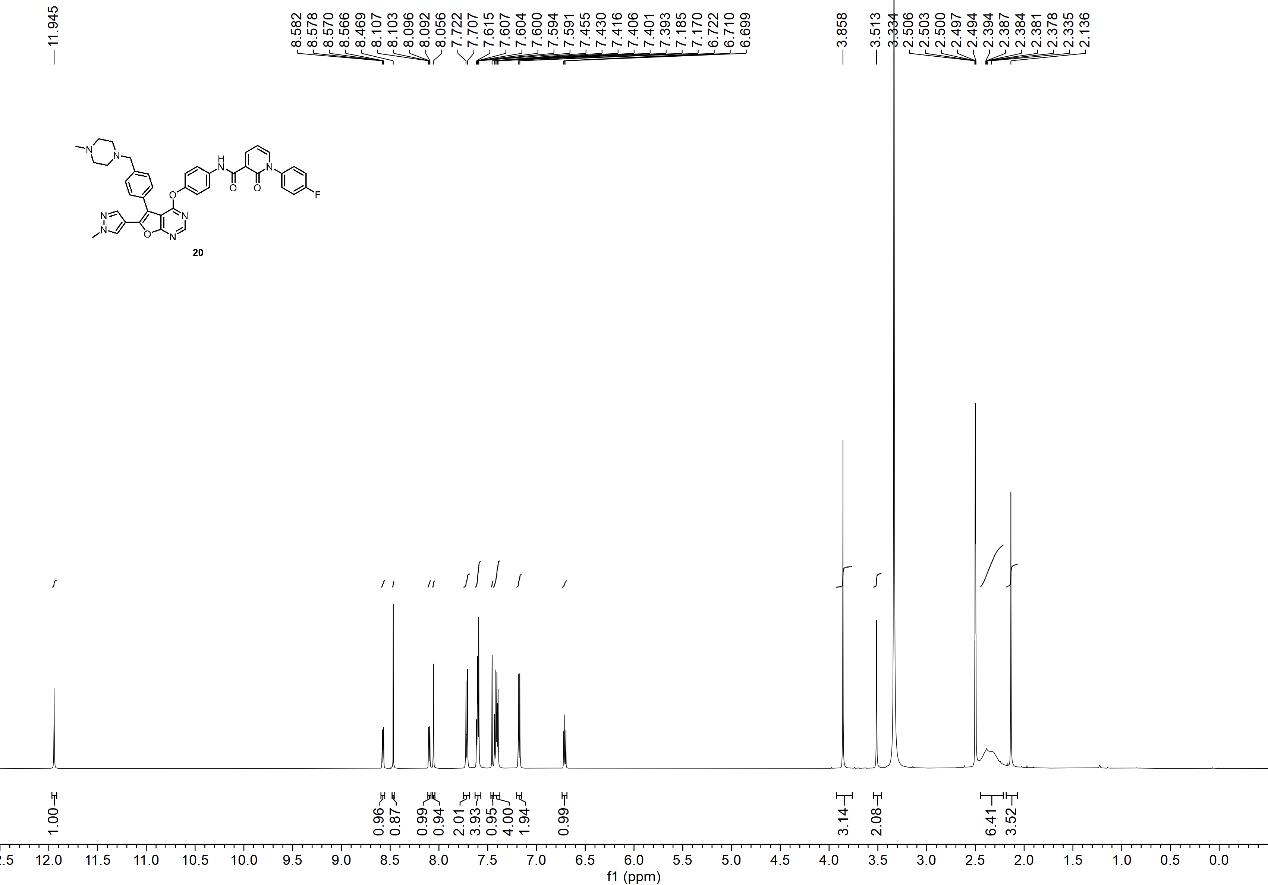


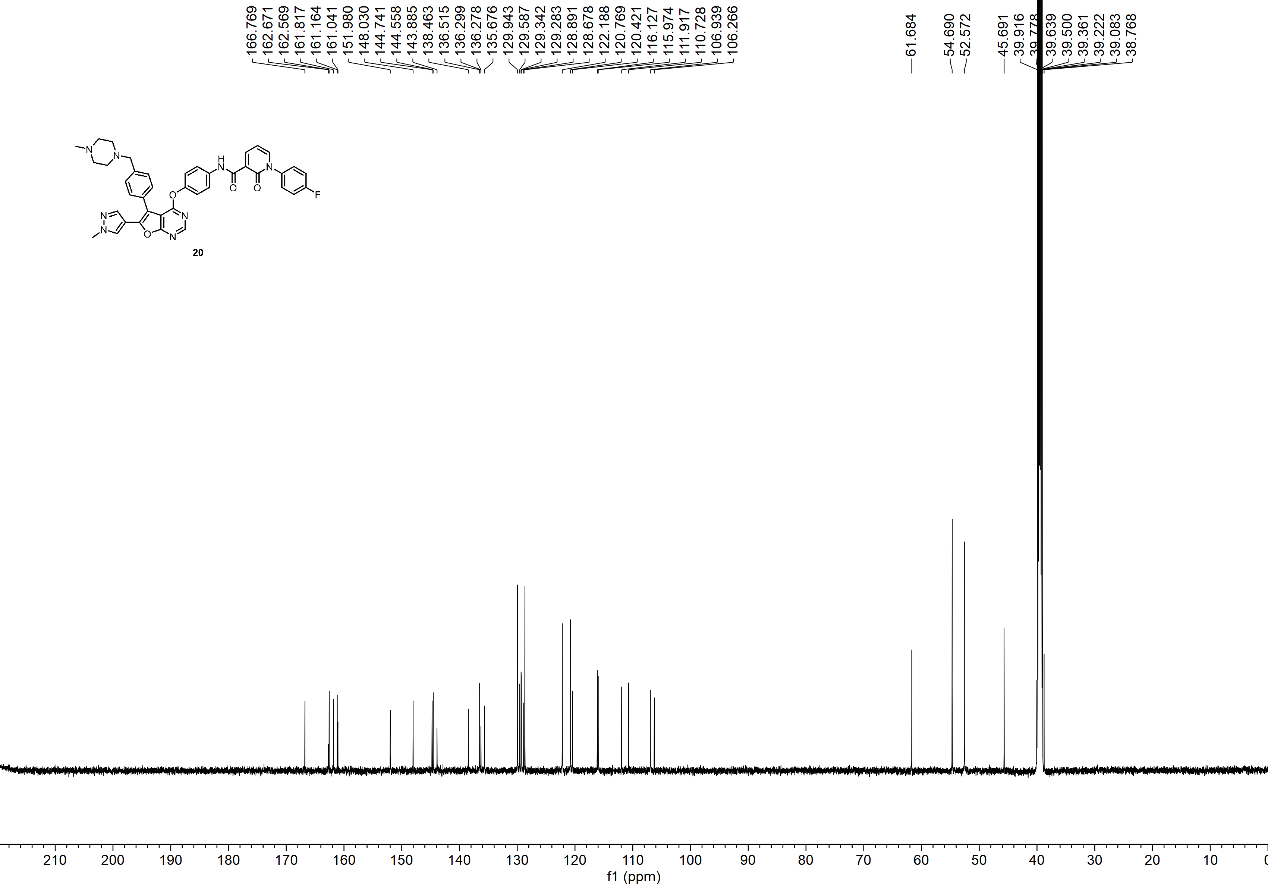


**
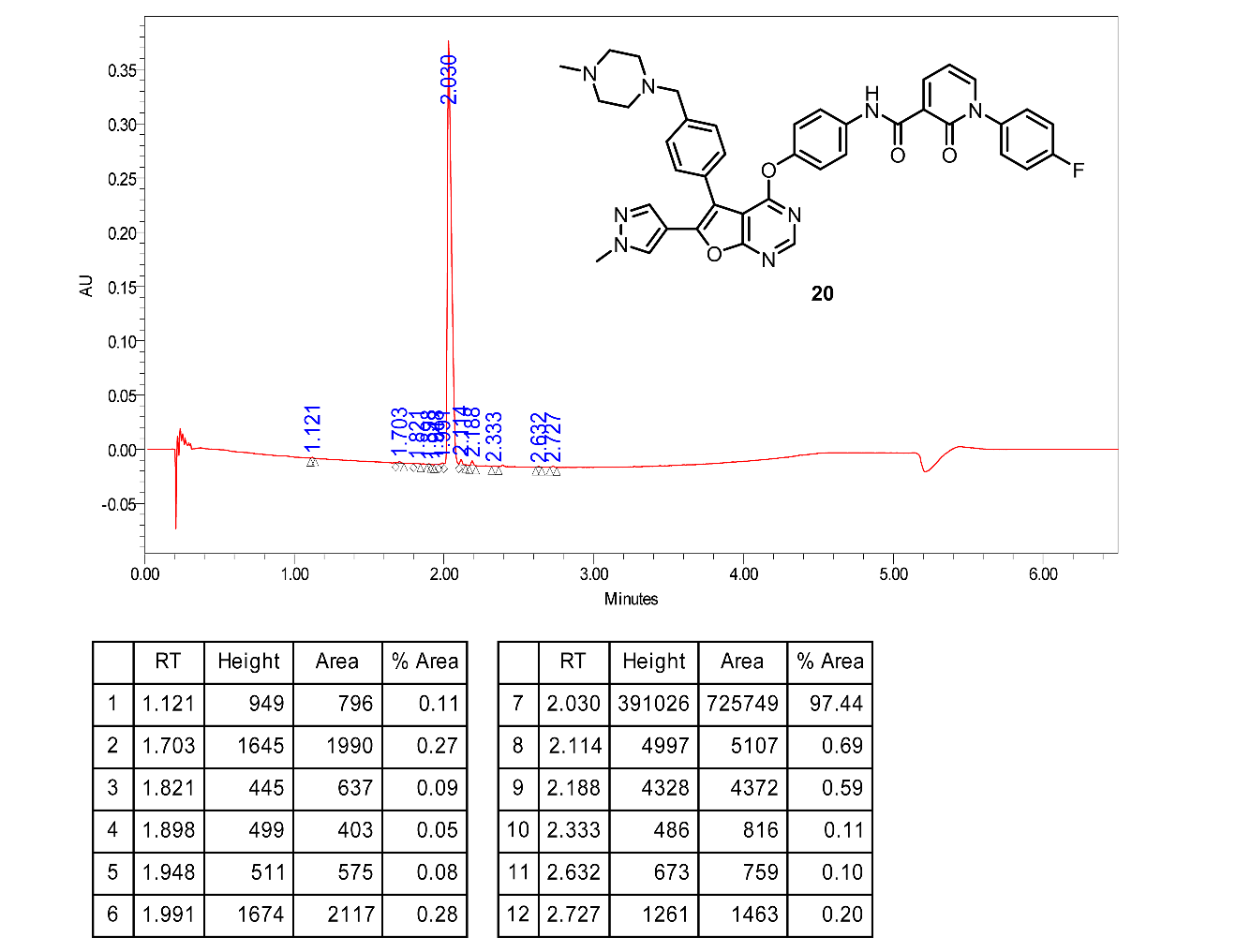
**


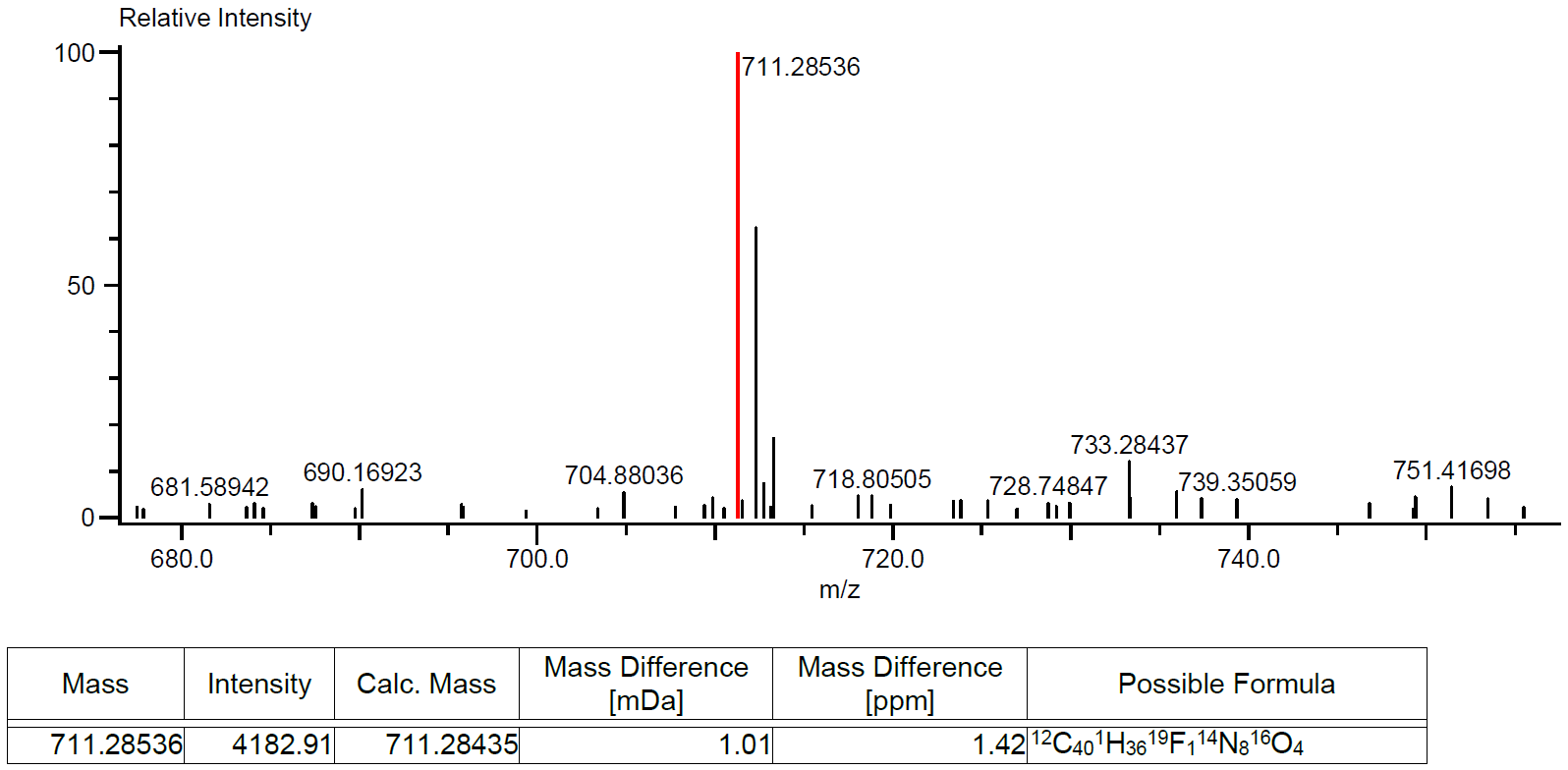


Figure S-16. ^1^H and ^13^C NMR spectra in DMSO-*d*_6_, HPLC trace, and HRMS data of compound **20**_._


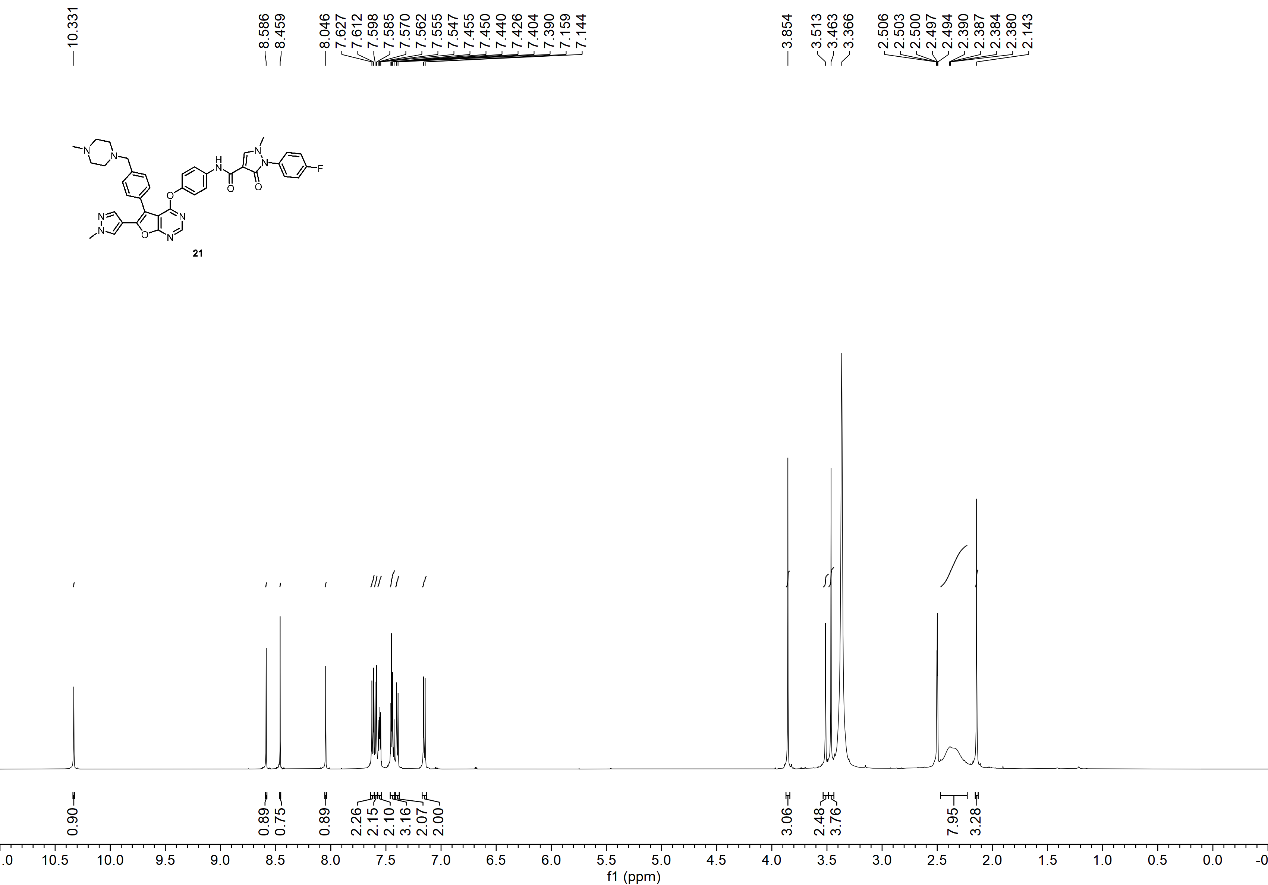


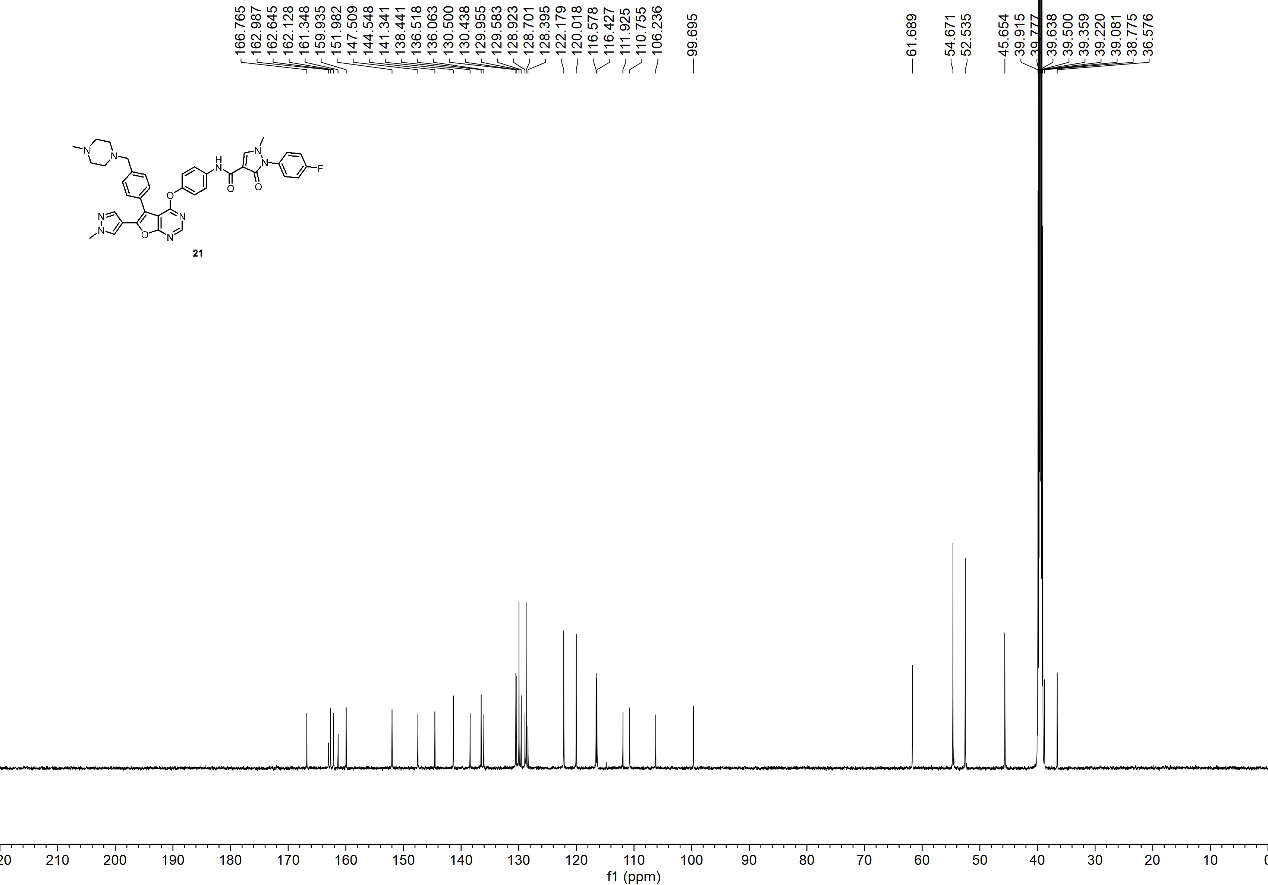


**
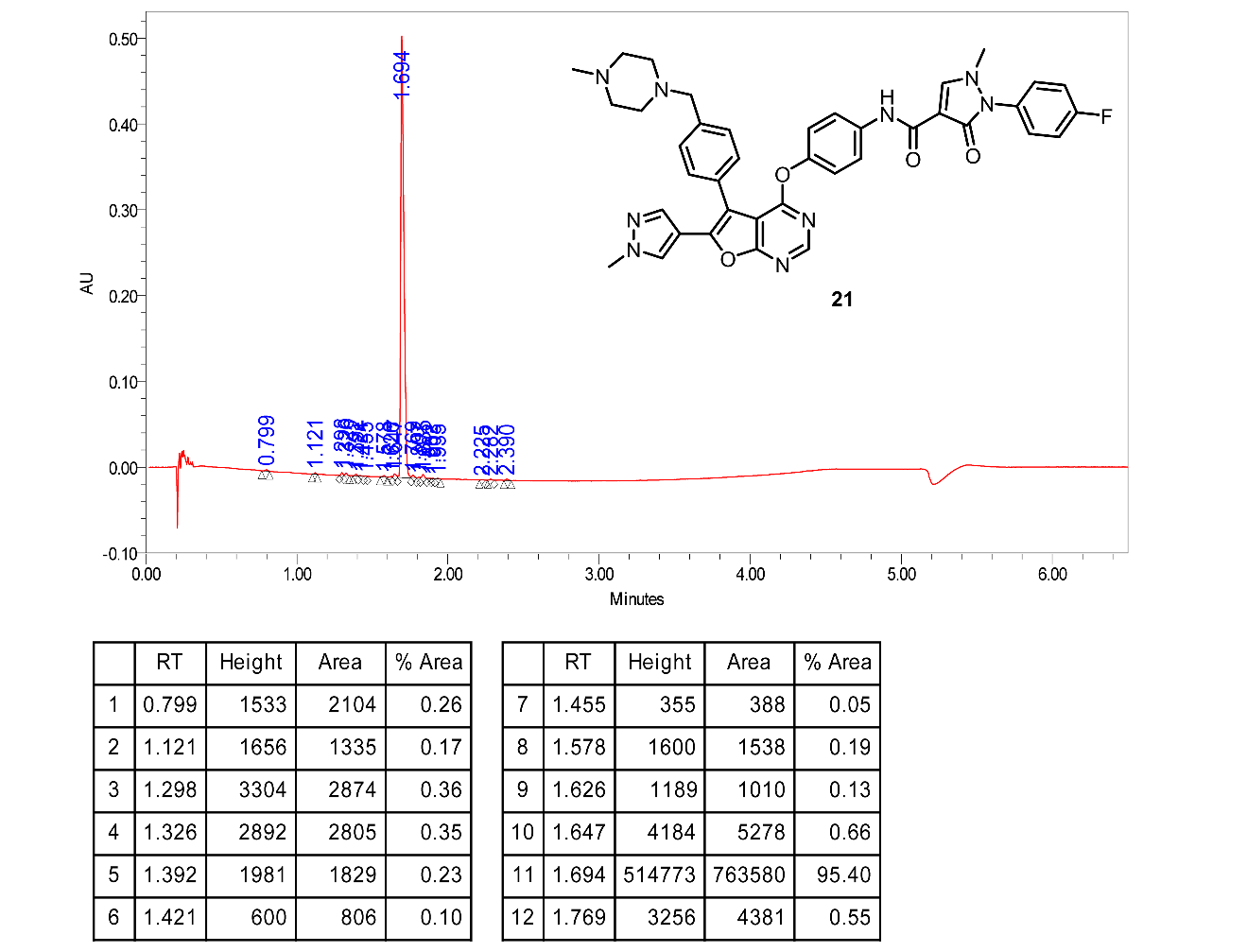
**


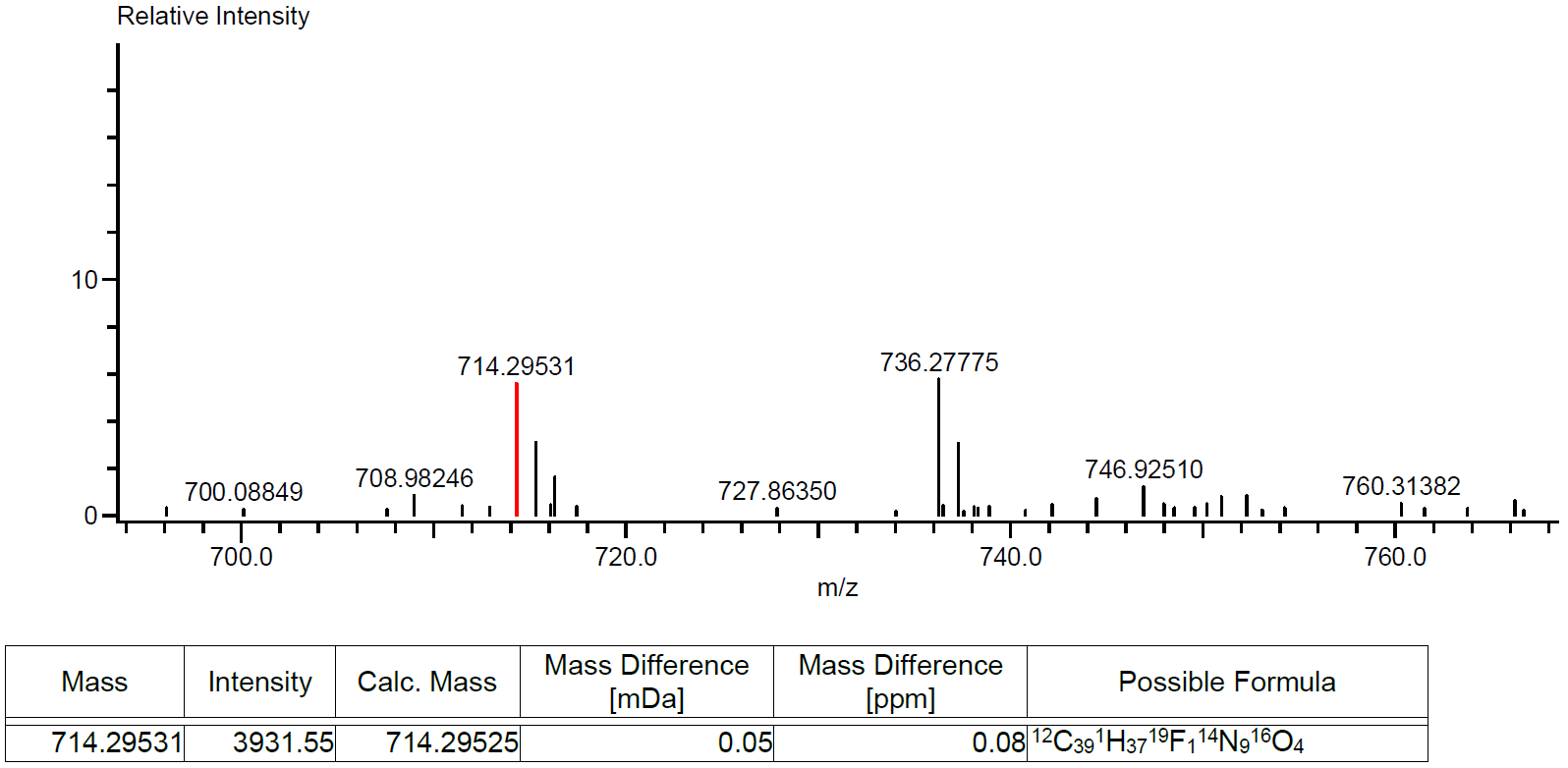


Figure S-17. ^1^H and ^13^C NMR spectra in DMSO-*d*_6_, HPLC trace, and HRMS data ^1^ of compound **21**_._
